# Functionalized screen-printed electrodes for non-invasive detection of vascular-endothelial cadherin in extracellular vesicles†

**DOI:** 10.1039/d4ra08926j

**Published:** 2025-04-22

**Authors:** William Meza-Morales, Sahimy Ayus-Martinez, Jesus Jimenez-Osorio, Maria Buendia-Otero, Luis López, David Suleiman, Edu Suarez, Donald O. Freytes, Lisandro Cunci, Camilo Mora

**Affiliations:** a Department of Chemical Engineering, University of Puerto Rico-Mayaguez Route 108 Mayaguez Puerto Rico USA camilo.mora@upr.edu; b Department of Chemistry, University of Puerto Rico-Rio Piedras 601 Av. Universidad San Juan Puerto Rico USA; c Department of Biology, University of Puerto Rico-Ponce Av. Santiago de los Caballeros Ponce Puerto Rico USA; d Lampe Joint Department of Biomedical Engineering, The University of North Carolina at Chapel Hill/North Carolina State University 4130 Engineering Building III, Campus Box 7115 Raleigh NC 27695 USA

## Abstract

In this study, we developed a biosensor using a gold screen-printed electrode (Au-SPE) functionalized with mercaptoundecanoic acid (MUA) and an antibody for detecting the vascular-endothelial cadherin (CD144) as a endothelial biomarker protein on extracellular vesicles (EVs) isolated from saliva. The MUA functionalization provides a stable platform for immobilizing the CD144 antibody, ensuring the detection of the target protein. This biosensor combines Au-SPE technology with an immunoassay, offering a rapid, sensitive, and non-invasive method for detection of CD144 carried by EVs. Characterization of saliva-derived EVs using transmission electron microscopy (TEM) and nanoparticle tracking analysis (NTA) confirmed their morphology and size, which fell within the expected range of 80–180 nm. NTA indicated a lower concentration of particles in saliva-EVs than in serum-EVs (controls), highlighting the need for sensitive detection of EV cargos in this type of EV. Immunodetection confirmed the presence of CD144 in both saliva and serum-derived EVs, with higher concentrations in serum. Functionalization of Au-SPEs with MUA and CD144 antibodies was confirmed by significant resistance changes, and atomic force microscopy (AFM) was used to verify the preservation of EV morphology and their capturing post-immune adsorption. A calibration curve demonstrated the high sensitivity of the biosensor prototype for detecting CD144-positive EVs, with a limit of detection (LOD) of 0.111 ng mL^−1^ and a limit of quantification (LOQ) of 0.37 ng mL^−1^, requiring only 3 μL of EV-sample. This biosensor shows potential as a novel method for detecting and studying endothelial biomarkers associated with cardiovascular disease in EVs isolated from saliva, a capability not currently available with existing tools. Furthermore, it provides a key platform for expanding research to other biomarkers and diseases by monitoring protein cargos in the EVs, enhancing its utility across diverse clinical applications.

## Abstract 2

Detection of endothelial biomarker vascular-endothelial cadherin (CD144) on saliva-derived extracellular vesicles using a gold screen-printed electrode biosensor functionalized with mercaptoundecanoic acid and anti-CD144 antibody.

## Graphical abstract

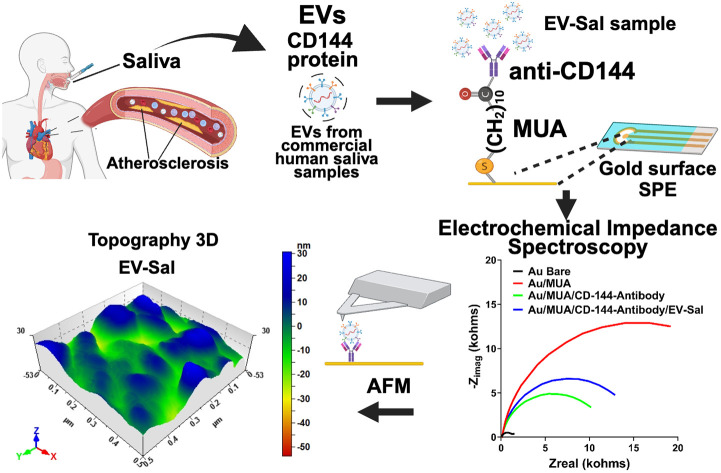

## Introduction

Early detection and monitoring of cardiovascular diseases (CVD) are vital for improving patient outcomes and reducing healthcare costs.^1,2^ One potential strategy involves identifying and measuring endothelial biomarkers indicative of vascular health and disease states, such as CD144. Vascular-endothelial cadherin (CD144), also known as VE-cadherin, is a transmembrane protein critical for maintaining endothelial cell-to-cell adhesion and vascular integrity.^3,4^ CD144 has been studied under the context of respiratory diseases, and it plays a pivotal role in the pathogenesis of various cardiovascular diseases, including atherosclerosis, hypertension, ischemia-reperfusion injury, and heart failure.^5,6^ In atherosclerosis, abnormal expression or function of CD144 disrupts endothelial cell junctions, compromises the endothelial barrier, and promotes plaque formation through increasing permeability, inflammation, and reducing plaque stability.^7^

CD144 dysregulation contributes to endothelial dysfunction in hypertension, exacerbates tissue damage during ischemia-reperfusion injury, and impairs vascular function in heart failure.^8,9^ As a biomarker, CD144 offers significant potential for the early detection and monitoring of CVD. Alterations in CD144 concentration in body fluids can serve as an early indicator of endothelial dysfunction, preceding clinical manifestations of CVD, and enabling the identification of individuals with high risk of developing CVD.^10,11^ Moreover, monitoring CD144 levels in patients with established cardiovascular conditions can provide insights into disease progression and therapeutic response.^12,13^ CD144 measurement could significantly enhance the management and outcomes of patients with CVD by providing valuable information on endothelial health and disease states. CD144 may act as an essential tool with the potential to enhance patient care and prevent adverse cardiovascular events.^11,14,15^

Current methods for detecting CD144, such as enzyme-linked immunosorbent assays (ELISA), flow cytometry, and western blotting, are well-established and widely used in clinical and research settings.^16,17^ However, these techniques come with limitations. They often require large sample volumes, extensive processing steps, and sophisticated laboratory infrastructure, making them unsuitable for rapid diagnostics and continuous monitoring. Furthermore, these methods aren't ideally suited for real-time analysis, which is becoming increasingly essential for timely clinical interventions.^18,19^

Another critical factor is that serum is derived from blood plasma, which circulates throughout the body *via* the cardiovascular system. Blood plasma constantly exchanges substances with the interstitial fluid surrounding tissues, ensuring a continuous supply of proteins and other molecules to various organs and tissues. As a result, the serum contains a rich and diverse array of proteins derived from systemic circulation.^20,21^ Saliva, on the other hand, is produced by salivary glands located within the oral cavity. While salivary glands receive blood supply from arteries, saliva's composition is more locally regulated and may work as a barrier to the serum's systemic protein profile.^22,23^ This fact means that biosensors that use biomarkers from saliva samples are limited by their concentration, so they have to generate detection limits low enough to capture these analytes in saliva samples.

We studied extracellular vesicles (EVs) because they have the potential as biomarkers for several diseases. EVs are lipid bilayer-enclosed particles naturally released from cells and present in various body fluids, including saliva. These vesicles carry surface proteins and other biomolecules reflective of their cell of origin, making them excellent candidates for biomarker discovery and diagnostic applications.^24–26^ EVs from saliva have been reported as cargoes of endothelial-related proteins, such as CD144, becoming novel biomarkers.^27–29^ The isolation and characterization of EVs from saliva offer a non-invasive, easily accessible source of biomarkers, including CD144. Leveraging EVs isolated from saliva presents a promising approach for developing novel biosensing technologies.^30–32^

Recent advancements in biosensor technology have enabled the development of sensitive detection platforms capable of operating in complex biological matrices.^33–35^ Screen-printed electrodes (SPEs) are particularly attractive for biosensor development due to their low cost, ease of fabrication, and potential for miniaturization.^36–38^ SPEs offer a robust platform for electrochemical biosensing, enabling rapid, portable, and cost-effective detection of biomarkers, including those associated with breast cancer and inflammation.^39,40^ Their versatility allows for functionalization with a wide range of chemical and biological recognition elements, significantly enhancing selectivity and sensitivity for detecting low-abundance analytes.^41^ When combined with functionalization techniques, such as applying 11-mercaptoundecanoic acid (MUA) to create a self-assembled monolayer, SPEs can be tailored to achieve detection for target analytes.^42,43^

In this study, we propose the construction of a novel biosensor utilizing an Au-SPE functionalized with MUA and an antibody for detecting CD144 protein carried by EVs isolated from saliva. The functionalization with MUA provides a stable and reactive platform for immobilizing the CD144 antibody, ensuring the binding and detection of the target protein. This biosensor aims to combine the benefits of Au-SPE technology with immunoassays, offering a rapid, sensitive, and non-invasive method for CD144 detection.

## Methodology

### Materials

We obtained all EVs commercially for this study. EVs: exosomes from pooled human saliva (Healthy Donors) (EV-Sal) (System Biosciences, EXOP-510A-1); exosomes from pooled human serum (Healthy Donors) (EV-Serum) (System Biosciences, EXOP-500A-1); exosome standards, fluorescent, recombinant, expressed in human cells (EV-GFP) (MilliporeSigma, SAE0193-1VL) (see ESI† for the isolation procedure performed by the supplier).

Dulbecco's phosphate buffered saline (DPBS) without calcium, magnesium and phenol red (Genesee Scientific, 25-508); bovine serum albumin (BSA) fraction V, protease free (Sigma-Aldrich, 3117332001); human VE cadherin ELISA kit (Abcam, ab210968); mouse anti-human CD144 antibody (Bio-Rad, MCA6119); goat anti-rabbit IgG H&L (HRP) (Abcam, ab205718); 1× Tris buffered saline (TBS) with 1% casein (Bio-Rad, 1610782EDU); 11-mercaptoundecanoic acid (MUA) (Sigma-Aldrich, 450561-5G); 10× TBS (Bio-Rad, 1706435); 10% tween 20 solution (Bio-Rad, 1610781); ethanol, anhydrous (histological) (Fisher Chemical, A405P-4); screen printed electrode AC1 (2 mm gold working; silver/silver chloride reference) (BASI, SP-1102); *N*-(3-dimethylaminopropyl)-*N*′-ethylcarbodiimide hydrochloride (EDAC, hydrochloride) (MilliporeSigma, 341006); potassium chloride (KCl), molecular biology grade (MilliporeSigma, 529552-250GM); potassium ferricyanide (K_3_[Fe(CN)_6_]) (Sigma-Aldrich, 244023); potassium ferrocyanide (K_4_[Fe(CN)_6_]) (Sigma-Aldrich, P9387); NanoSight LM100 system (Malvern Panalytical Instruments Ltd); ChemiDoc touch instrument (Bio-Rad); multiplate reader (Spark, TECAN); Multiskan GO with SkanIt software 5.0 (Thermo Scientific); Jeol 1230 TEM; potentiostat interface 1010E (Gamry Instruments) with Gamry DigiElch 8 software (Gamry Instruments); NT-MDT instrument (SMENA head) operating in tapping mode with an NSG-10 tip (240 kHz resonant frequency, 11.8 N m^−1^ force constant) at room temperature.

### Methods

#### Nanoparticle tracking analysis (NTA)

EVs were analyzed by a NanoSight LM100 system (Malvern Panalytical Instruments Ltd). Nano tracking analysis videos were recorded and analyzed using the NTA software (version 3.1) with the camera level set to 11 and the detection threshold at 3. All analyses were conducted at room temperature. The samples were diluted in 0.22 μm filtered DPBS (Genesee Scientific). In the field of view, there were approximately 20–40 particles. The trajectory movement of particles in sight was monitored and averaged from 3 videos of 60 seconds each to obtain the particle number and size distribution. Concentrations and particle size distribution of at least 3 different EV samples were recorded and analyzed.

#### Protein quantification

The presence of proteins in the samples was quantified using a standard protein quantification assay (BCA Protein Assay Kit, Millipore Sigma) with Bovine Serum Albumin (BSA) as the standard protein, following the manufacturer's instructions. Briefly, 25 μL of each sample and BSA standard was pipetted into a transparent 96-well microplate, and 200 μL of the BCA working reagent was prepared by mixing Reagent A (BCA solution) and Reagent B (cupric sulfate solution) in a 50 : 1 ratio, was added to each well. The plate was incubated at 60 °C for 15 minutes (mins) to allow for color development. A calibration curve was constructed using BSA standards at concentrations of 5, 10, 50, 125, and 250 μg mL^−1^ (see Fig. S1†), and sample protein concentrations were determined from this curve. All measurements were performed in triplicate to ensure accuracy and reproducibility, and blank wells with DPBS were included to correct for background absorbance. Absorbance was measured at 562 nm using a Multiskan GO with SkanIt software 5.0 (Thermo Scientific).

#### Transmission electron microscopy (TEM)

Copper carbon formvar 400 mesh grids were glow discharged before loading the sample. The grid was floated on the drop on the sample for 10 min and washed two times by floating on a drop of filtered deionized water (DI water), followed by a negative stain using 2% uranyl acetate. The excess of the stain was blotted with Whatman paper, and the grid was air-dried. The sample was imaged using Jeol 1230 TEM.

#### Immunoblotting (dot blot) analysis (DBA)

All EVs were immobilized in nitrocellulose membrane for 10 min at room temperature and blocked with 1% bovine serum albumin in DPBS for 16 hours. Then, EVs were incubated for 2.5 hours with anti-CD144, CD81, and CD9 antibodies (BioRad and SBI) at 1 : 400 dilution, washed with TBST, and incubated for 40 min at room temperature with secondary antibody (Abcam) at 1 : 800 dilution. Samples were washed with TBST and counter-stained with luminol. Images were obtained with a ChemiDot touch instrument (Bio-Rad) and processed using Image Lab.

#### Functionalization of Au screen-printed electrodes (Au-SPEs)

The electrodes were first cleaned with DPBS and ethanol. Then, the gold working electrode in the Au-SPEs was immersed in 10 mM 11-mercaptoundecanoic acid (MUA) for 3 hours at 4 °C to create a thiol-terminated self-assembled monolayer (SAM). Afterward, the surface was rinsed with ethanol and DPBS. The electrodes were placed in a solution of EDAC (5 μM) and CD144 antibody (1 : 400) for 36 hours at 4 °C. Finally, the Au-SPE surface was washed with DPBS to remove any remaining solution before use.

#### Electrochemical measurements

Electrochemical measurements were conducted using a three-electrode cell, with gold electrodes as the working electrodes, a platinum wire as the counter electrode, and an Ag|AgCl reference electrode filled with 3 M KCl. A ref. 600+ Gamry potentiostat was employed, and data were recorded using Gamry Instruments Framework software. The DC potential of 0.2 V was referenced against Ag|AgCl. For EIS, an amplitude of 10 mV was applied, with frequencies ranging from 1 MHz to 10 Hz. A solution of 5 mM K_3_[Fe(CN)_6_]/K_4_[Fe(CN)_6_] in 0.1 M KCl was used for all EIS experiments.

#### Atomic force microscopy (AFM)

AFM measurements were performed in an Agilent 5500 atomic force microscope in tapping mode. The scanned area size was 0.5 × 0.5 μm at a resolution of 512 pixels per line with a total of 512 lines per image and a scanning speed of 1 line per s. The software Nanoscope8 was used for data analysis.

#### Enzyme-linked immunosorbent assay (ELISA)

An ELISA kit for CD-144 (VE-cadherin) Ab-based detection of EVs was purchased from Abcam. Samples were handled according to the manufacturer's protocol. Optical density was measured at 450 nm using a multiwell plate reader (Spark, TECAN).

#### Calibration curve

To establish a calibration curve for CD144-positive extracellular vesicles (EV-Sal), CD144 protein standards of EV-Sal were prepared at concentrations ranging from 0.181 ng mL^−1^ to 0.809 ng mL^−1^. The assay utilized a biosensor based on an Au-SPE functionalized with an anti-CD144 antibody. Measurements were conducted using a Gamry potentiostat (Interface 1010E). For each standard (the concentrations used were 0.181, 0.3458, 0.36, 0.5615, and 0.809 ng CD144 per mL, corresponding to total protein concentrations of 69.036, 131.893, 137.309, 214.164, and 308.564 μg mL^−1^, respectively), 3 μL of EV-Sal was applied to the electrode surface. The biosensor's response to CD144 was assessed by measuring impedance changes at a set potential. The resulting calibration curve plotted as the signal *versus* known CD144 concentrations demonstrated linearity within the tested range. To ensure reproducibility, all assays were performed in triplicate. Background signals were corrected by including blank samples (DPBS), ensuring the method's accuracy and reliability for detecting and quantifying CD144-positive EVs.

## Results

**Fig. 1:**
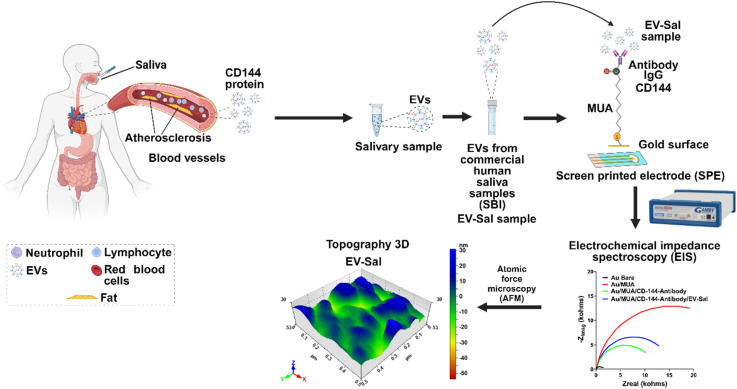
Overview.

In this investigation, we utilized a method for detecting CD144 protein on extracellular vesicles from human saliva, which is linked to atherosclerosis in blood vessels (Fig. 1). These EVs were obtained and isolated from commercial human saliva samples (Healthy Donors) by System Biosciences (SBI), human serum-EVs (Healthy Donors), and EV-GFP+ (negative control for endothelial-related CD144 marker). The isolated EVs are then immobilized on a gold surface of a Au screen-printed electrode using an antibody specific to CD144 (IgG CD144) and a linker molecule (MUA). The Au-SPE was analyzed using EIS, a technique that measures the impedance at the electrode–electrolyte interface to provide critical insights into surface modifications and molecular interactions. Specifically, EIS monitors changes in charge-transfer resistance (*R*_ct_) during each functionalization step: the gold surface (baseline measurement), the formation of an MUA layer, antibody immobilization, and EV-Sal capture. The increase or decrease in *R*_ct_ at each stage reflects the successful stepwise assembly of the biosensor and the specific binding of CD144-positive EVs. This quantitative data confirms the presence of CD144 on the captured EVs, as the vesicles further hinder electron transfer at the electrode surface. The EV-Sal sample captured by the immune biosensor confirmed through EIS, was further analyzed using AFM to validate the successful capture and preservation of CD144-positive EV-Sal, providing orthogonal characterization for enhanced reliability.

### Characterization of extracellular vesicles

**Fig. 2:**
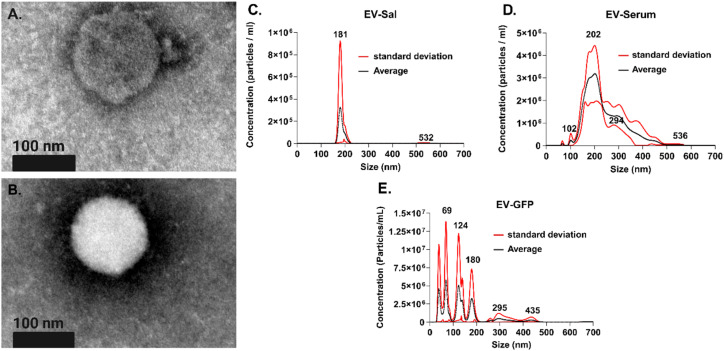
(A and B) Transmission electron microscopy image of EV-Sal. The scale bar represents 100 nm. (C–E) Nanoparticle tracking analysis of EV-Sal, EV-Serum and EV-GFP.

Transmission electron microscopy (TEM) was employed to scrutinize the morphological characteristics of the EVs previously isolated from human saliva. As depicted in Fig. 2A and B, the TEM images revealed the presence of particles with characteristic morphology of lipid membrane layers in these EVs, with diameters ranging from 121 to 141 nm, consistent with reported EV sizes. The suppliers characterized the isolated EVs in size and concentration (see Fig. S2†). Furthermore, nanoparticle tracking analysis (NTA) was conducted to validate the size distribution of the purchased EVs, as illustrated in Fig. 2C–E. The NTA showed a concentration of particles per mL of 7.69 × 10^6^ ± 5.05 × 10^6^, 4.45 × 10^8^ ± 2.00 × 10^7^, and 7.96 × 10^8^ ± 3.05 × 10^7^ particles per mL for EV-Sal, EV-Serum, and EV-GFP, respectively.

### Immunodetection

**Fig. 3:**
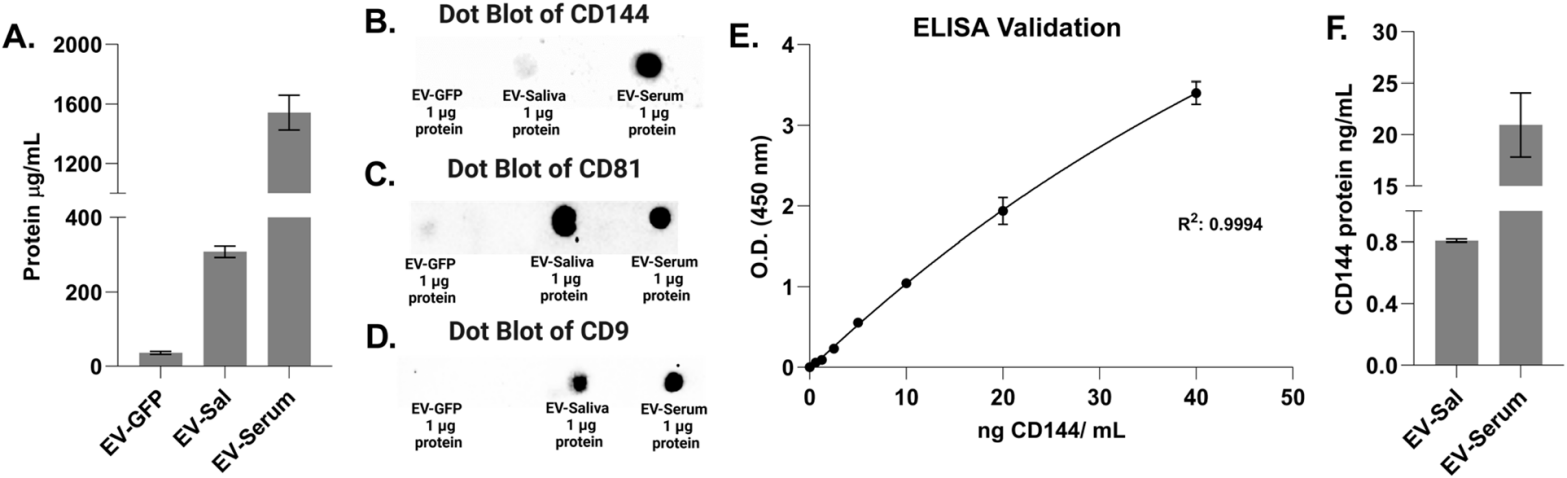
(A) Protein quantification by BSA for EV-GFP, EV-Sal, and EV-Serum, *n* = 3. (B–D) Dot Blot tests of CD144, CD81, and CD9 proteins for EV-GFP, EV-Sal, and EV-Serum. (E) Calibration curve of ELISA test for CD144, and (F) Quantification of CD144 for EV-Sal and EV-Serum, *n* = 3.

The protein concentration of EV-GFP was determined to be 36.120 ± 3.992 μg mL^−1^. This concentration falls within the expected range for many biological samples. EV-Sal exhibited a significantly higher protein concentration of 308.564 ± 15.405 μg mL^−1^, indicating a notably higher protein content than EV-GFP. On the other hand, EV-Serum showed the highest protein concentration among the three samples, measuring at 1.542 817 ± 116.674 μg mL^−1^. This remarkably high concentration suggests a substantial amount of protein present in EV-Serum (see Fig. 3A).

Upon Dot Blot analysis, both EV-Sal and EV-Serum exhibited positive signals on the nitrocellulose membrane, indicating the presence of the CD144 protein. These positive results were observed as individual black spots at the corresponding locations where the samples were put on the membrane (An additional Dot Blot was performed by decreasing the protein concentration of EV-Serum to better visualize the EV-Sal signal, see Fig. S3†). In contrast, EV-GFP showed no signal, suggesting a low target amount or the absence of protein in this sample. Similarly, the Dot Blot assays aimed at detecting the CD81 and CD9 proteins yielded positive results, affirming the presence of these proteins within EV samples. Moreover, the Dot Blot assays targeting the CD81 and CD9 proteins demonstrated consistent positive signals across EV-Sal and EV-Serum. CD9 biomarker was not detected in the immune blot for the EV-GFP. This observation confirms the presence of this essential biomarker, which is characteristic of extracellular vesicles (see Fig. 3B–D).

The ELISA analysis revealed distinct concentrations of human CD144 protein in the extracellular vesicles derived from saliva and serum. The concentration of CD144 in extracellular vesicles from saliva was relatively low, measuring at 0.809 ± 0.0010 ng mL^−1^. In contrast, extracellular vesicles from serum exhibited a significantly higher concentration of CD144, with a value of 20.956 ± 3.119 ng mL^−1^ (see Fig. 3E and F).

Table 1 shows the characterization of EVs derived from different sources, including GFP-tagged EVs, saliva-derived EVs, and serum-derived EVs. The analysis focused on total protein content, CD144 protein concentration, and the number of particles per milliliter. The total protein content varied significantly among the different types of EVs.

**Table 1:** Amount of total protein, target protein (CD144 protein), and number of particles per mL concentration

EVs	Total protein μg mL^−1^	CD144 protein ng mL^−1^	# Particles per mL
EV-GFP	36.120 ± 3.992	—	7.96 × 10^8^ ± 3.05 × 10^7^
EV-saliva	308.564 ± 15.405	0.809 ± 0.0010	7.69 × 10^6^ ± 5.05 × 10^6^
EV-serum	1542.817 ± 116.674	20.956 ± 3.119	4.45 × 10^8^ ± 2.00 × 10^7^

EV-GFP exhibited a total protein content of 36.120 ± 3.992 mg mL^−1^. In contrast, EV-Saliva showed a considerably higher protein content of 308.564 ± 15.405 mg mL^−1^, highlighting the complex protein composition of saliva-derived vesicles. The highest protein concentration was found in EV-Serum at 1542.817 ± 116.674 mg mL^−1^, significantly higher than in EV-GFP, reflecting the protein-rich serum environment. We found distinct differences when examining the CD144 protein concentration, a known endothelial biomarker, *via* ELISA. CD144 was not detected in EV-GFP, while EV-Saliva contained 0.809 ± 0.0010 ng mL^−1^ of CD144. Although this level is lower than that found in EV-Serum (20.956 ± 3.119 ng mL^−1^), it is still significant, suggesting that saliva-derived EVs can serve as valuable non-invasive biomarkers for endothelial health. The concentration of EVs (particles) per milliliter also showed notable variation. EV-GFP had a concentration of 7.96 × 10^8^ ± 3.05 × 10^7^ particles per mL, whereas EV-Saliva had a considerably lower concentration of 7.69 × 10^6^ ± 5.05 × 10^6^ particles per mL. EV-Serum, with 4.45 × 10^8^ ± 2.00 × 10^7^ particles per mL, again exhibited higher vesicle concentrations, indicative of the dense vesicular environment of serum. The NTA also shows that EV-Sal has a one-peak size distribution compared to EV-GFP and EV-Serum.

### Functionalization and detection of extracellular vesicles

**Fig. 4:**
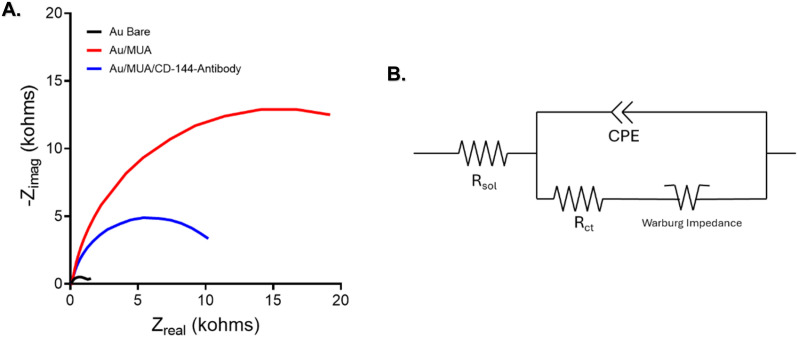
(A) EIS Nyquist plot of functionalization of the Au-SPE with MUA and Cd-144 antibody. (B) A model of CPE with diffusion was used in the simulation to calculate the electron-transfer resistance.

EIS was used to investigate the chemical changes on the surface of the antibody-modified gold (Au) working electrode. Fig. 4A presents EIS data for the bare Au electrode, Au modified with mercaptoundecanoic acid (Au/MUA), and Au after crosslinking with the CD144-targeted antibody (Au/MUA/CD144 antibody). The charge-transfer resistance (*R*_ct_) increased significantly from 3.768 kΩ to 33.95 kΩ following MUA modification, indicating a higher resistance to electron transfer due to the presence of MUA. This nearly 10-fold increase in *R*_ct_ reflects the impedance introduced by MUA on the electrode surface. Conversely, introducing the CD144 antibody reduced *R*_ct_ from 33.95 kΩ to 10.42 kΩ. This decrease suggests that the antibody facilitates electron transfer between the electrode surface and the redox probe solution (K_3_Fe(CN)_6_/K_4_Fe(CN)_6_), likely due to favorable interactions between the antibody and the Au electrode.

The electrode–electrolyte interface resembles an electrical circuit composed of a resistor and capacitor.^44,45^Fig. 4B illustrates the circuit model used to analyze the EIS of Au electrodes, specifically chosen for the redox couple K_3_Fe(CN)_6_/K_4_Fe(CN)_6_.

This study utilized AFM to analyze gold screen-printed electrodes functionalized with the CD144 antibody (see Fig. S4†) to detect EVs isolated from saliva. The AFM analysis provided detailed measurements of the size and height of the immune adsorbed and detected vesicles, which are crucial for validating the vesicle nature of the adsorbed sample while understanding their morphology and functionality.

**Fig. 5:**
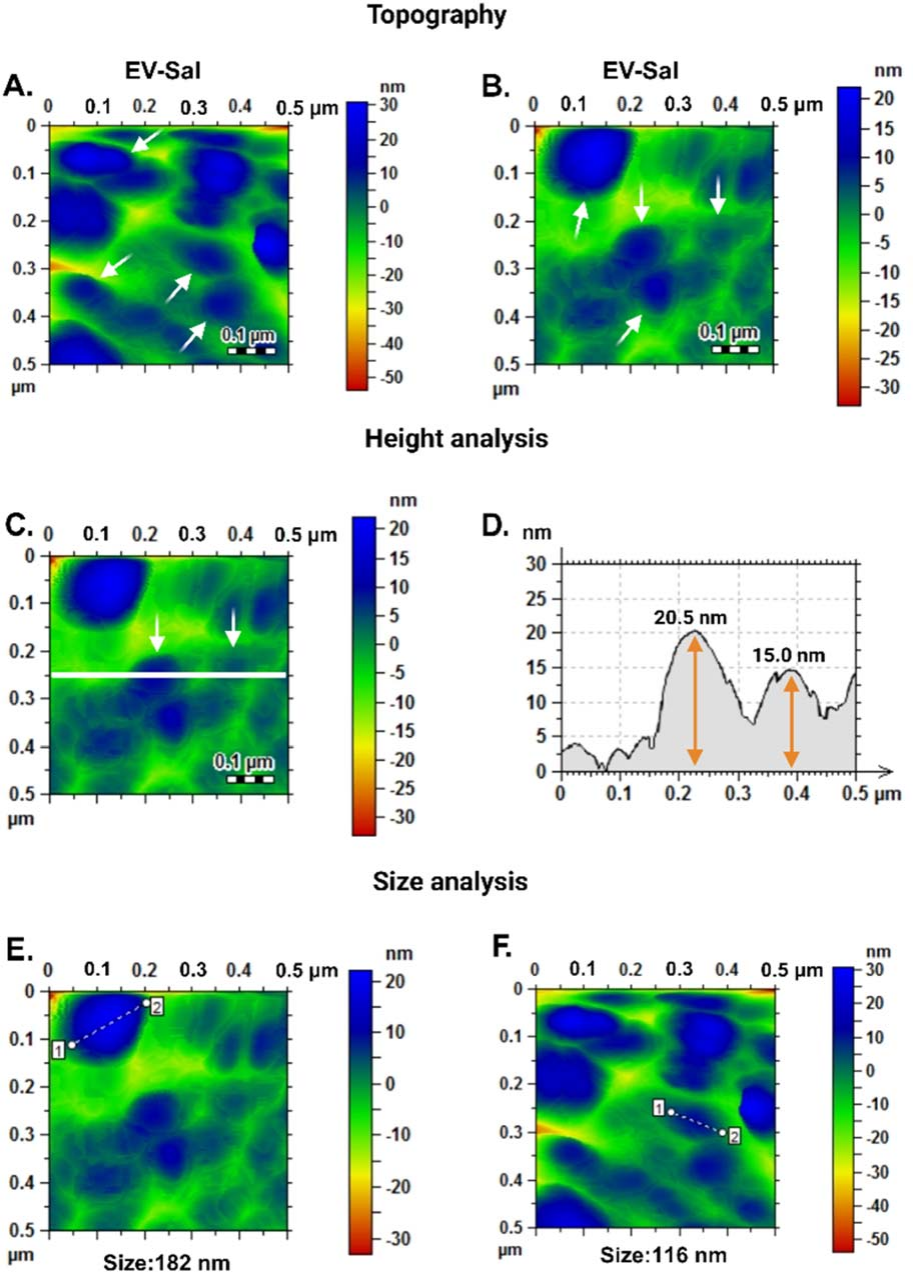
AFM topography images: (A and B) purified EVs from human saliva. (C and D) Height measurement analysis of purified EVs from human saliva on AFM topography image. (E and F) Distance measurement analysis of purified EVs from human saliva in AFM topography image.

Fig. 5A and B present topography images of EV-Sal (+CD144) on gold screen-printed electrodes functionalized with the CD144 antibody. The images show the surface morphology with EVs, as the white arrows indicate. These topographical maps visualize the vesicles, highlighting their distribution and surface interaction on the electrodes. Fig. 5C and D display height analysis of the vesicles conducted at 250 nm, a significant aspect of our research. The height measurements revealed two populations of vesicles with heights of 20.5 nm and 15 nm. This height data is essential for confirming EVs' successful capture and intact nature on the functionalized electrodes. Fig. 5E and F show the size analysis of the EVs, a crucial step in our research. The AFM measurements determined that the diameter of the vesicles ranges from 182 nm to 116 nm in alignment with TEM results, validating our approach. The size analysis confirms the presence of various EV populations (size-wise) that are consistent with typical extracellular vesicle dimensions.^46,47^

**Fig. 6:**
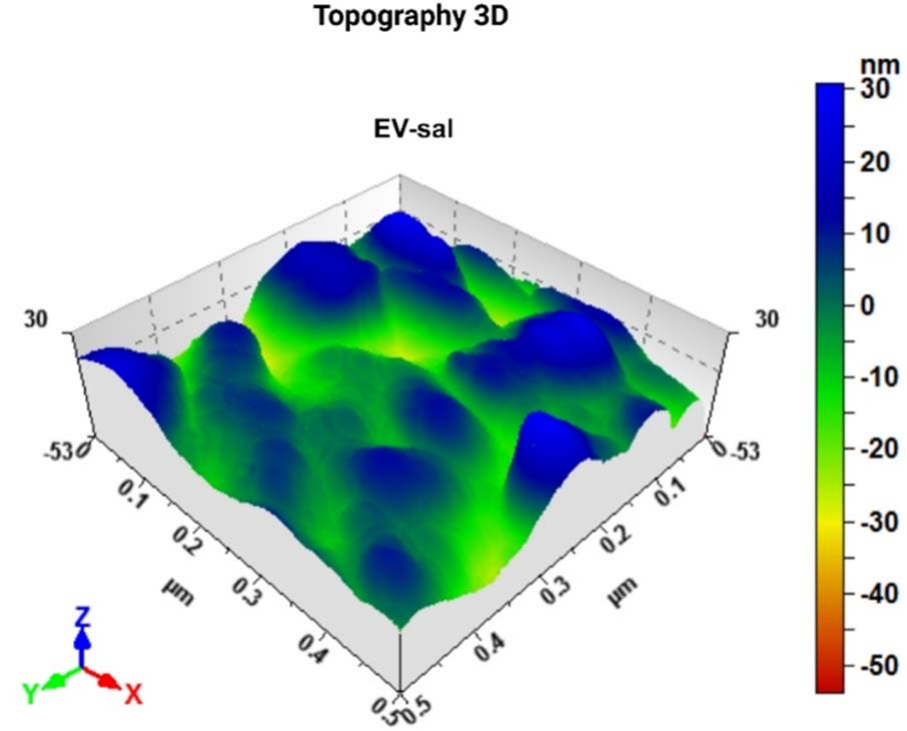
Image of 3D atomic force microscopy (AFM) topography of the biosensor surface after the capturing of EV-Sal.

The 3D topography images offered a detailed, three-dimensional view of the EV-Sal immune adsorbed on the electrode surface, with identifiable EV populations (see Fig. 6). The enhanced resolution provided by the 3D images allowed for precise measurement of both the lateral dimensions and the height of the EVs. The 3D topography images confirmed these size distributions, providing visual evidence of the vesicle sizes and shapes. The EV-Sal appeared spherical and uniformly distributed across the electrode surface, indicating successful capture by the functionalized antibodies. This detailed height information is crucial for understanding the spatial characteristics of the EVs and their interaction with the electrode surface.

### Calibration curve of extracellular vesicles from human saliva

**Fig. 7:**
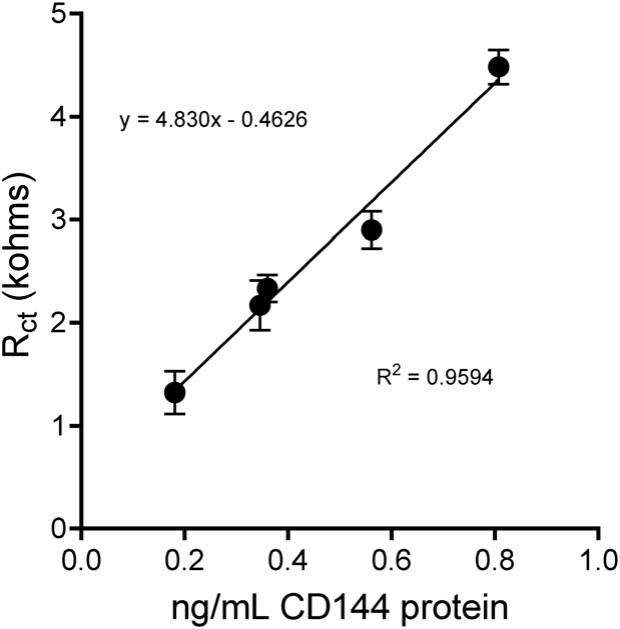
The calibration curve of the human CD144 biosensor with purified EVs from saliva, *n* = 3.

This study developed a calibration curve for EV-Sal targeting the CD144 protein, an endothelial biomarker (see Fig. S5–64†). Based on five data points, the curve covers a concentration range of 0.181 to 0.809 ng mL^−1^. The method demonstrated high sensitivity, with a quantification limit (LOQ) (LOQ = 10*σ*/*S*, *σ*: is the standard deviation of the response (impedance) of the blank samples, and *S*: is the slope of the calibration curve obtained from the linear regression of the impedance response *versus* the analyte concentration) of 0.37 ng mL^−1^ and a detection limit (LOD) (LOD = 3*σ*/*S*) of 0.111 ng mL^−1^, as shown in Fig. 7. The calibration curve exhibited linearity within this range, enabling precise quantification of CD144-positive EV-Sal. The method's maximum quantifiable amount was 1.11 pg, highlighting its capability to detect and measure min amounts of EVs. The analysis required only 3 μL of the sample, showcasing the method's efficiency (see Fig. 7).

## Discussion

This study presents a biosensor design using an Au-SPE functionalized with mercaptoundecanoic acid (MUA) and an antibody to detect CD144 protein on extracellular EVs isolated from saliva. MUA serves as a stable cross-linker, enabling effective immobilization of the CD144 antibody for accurate protein detection. This biosensor integrates the advantages of Au-SPE technology with immunoassays, offering a fast, sensitive, and non-invasive method for CD144 detection. The use of saliva-derived EVs presents significant challenges due to their lower biomarker concentrations compared to the biomarker-rich and extensively studied serum or plasma samples, which also require invasive collection methods. This limitation emphasizes the need for highly sensitive biosensors capable of detecting and quantifying biomarkers at low concentrations in saliva. Targeting CD144, a key endothelial biomarker associated with cardiovascular diseases, this approach can enhance early diagnosis and monitoring of cardiovascular conditions, ultimately improving patient outcomes and reducing healthcare costs.

### Characterization of extracellular vesicles

TEM provides detailed visualization of EVs, allowing for precise measurement of their size and morphology. This study's TEM images of EV-Sal revealed vesicles with diameters of 121 nm and 141 nm. These measurements fall within the typical size range of exosomes (30–150 nm), suggesting that the EVs isolated from saliva predominantly consist of small-size-like vesicles.^48,49^ The consistency in size observed in the TEM images indicates a relatively uniform population of vesicles, which is beneficial for their potential use as biomarkers.

This study explicitly details the TEM analysis of EV-Sal for their relevance as non-invasive biomarkers. Still, the uniformity in EV-Sal size provides a strong foundation for their characterization. Understanding EVs' morphology and size distribution is crucial, as these characteristics can influence their biodistribution, cellular uptake, and overall functionality as biomarkers. NTA results indicate a markedly lower concentration of EVs in saliva compared to serum and GFP-labeled samples. The high concentration of EV-Serum reflects the rich and complex nature of serum, which comprises many vesicles secreted from various cellular sources.^50,51^ Similarly, the EV-GFP also showed a high concentration, suggesting efficient labeling and isolation processes.

Moreover, In the analysis of EV samples using NTA, the homogeneity of the size distribution is crucial, especially for applications requiring standardization, such as biosensor calibration. Fig. 2C, which represents EV-Sal, exhibits a single, sharp peak around 181 nm, indicating a relatively homogeneous sample. This profile shown in the NTA is similar to that observed by the SBI supplier (see Fig. S2†). This uniformity in size distribution makes the EV-Sal sample particularly suitable as a standard for biosensors, ensuring consistent and reliable sensor performance. In contrast, Fig. 2D (EV-Serum) and 2E (EV-GFP) display multiple peaks, suggesting a heterogeneous size distribution with particles of varying sizes. Several distinct peaks in these plots indicate significant variability within the samples, which can complicate their use as standards due to potential inconsistencies in sensor responses. Therefore, while the heterogeneous EV samples in Fig. 2D and E are valuable for various analytical purposes, the homogeneity of the EV-Sal sample in Fig. 2C makes it a more appropriate choice for use as a standard in biosensor applications.

Despite the lower concentration of EV-Sal, the NTA data is valuable for understanding the potential yield and required sensitivity for downstream analyses. The significant standard deviation in the EV-Sal concentration highlights the variability in saliva samples, potentially due to differences in collection, handling, or individual biological variation. Standardized protocols must address this variability to ensure reproducibility and reliability when using saliva-derived EVs as biomarkers.

The TEM and NTA analyses comprehensively characterize the EVs from different sources. The TEM results indicate that some of the saliva-derived EVs are of a size within the exosomal range. Still, biomarkers and orthogonal characterizations are required to classify them as exosomes. Still, the nanosize is promising for its potential use as biomarkers. However, it remains to consider that the lower concentration observed in NTA might indicate that while saliva is a less invasive medium, it may require more sensitive detection and isolation techniques compared to serum.

### Immunodetection

The differences in protein concentrations among the three samples have several implications. Firstly, the varying protein levels may reflect differences in the nature and origin of the samples. For instance, EV-Serum contains a concentrated protein extract or a higher abundance of proteins than EV-GFP and EV-Sal. This is because serum contains a wide variety of proteins, including albumin, globulins, clotting factors, hormones, and enzymes.^52,53^ In contrast, saliva is a watery fluid secreted by the salivary glands. It is primarily composed of water, electrolytes, enzymes (such as amylase), antimicrobial agents, and mucins.^54,55^ While saliva contains proteins, its composition is less protein-rich than serum.

On the immunoblotting test (Dot Blot test), positive signals in EV-Sal and EV-Serum strongly suggest that they contain the CD144 protein.^56–58^ This finding aligns with our initial hypothesis and indicates that the protein is indeed present in these samples. However, the nondetection or absence of a signal in EV-GFP confirms its negative status for the protein, validating its use as a control in our experiment. Similarly, the Dot Blot assays aimed at detecting the CD81 and CD9 proteins yielded positive results by EV-Sal and EV-Serum, affirming the presence of this protein within these EV samples.^59–61^ EV-GFP is positive for CD81, but CD9 was not detected under these conditions, confirming only a single biomarker. This may be due to the low concentration of proteins in EV-GFP, which produces a low concentration of biomarkers.^62^

The ELISA assay of the CD144 protein showed that the differences in CD144 protein concentrations between the two samples have several implications. These findings suggest CD144 may be more abundant in extracellular vesicles derived from serum than saliva. This observation could be attributed to differences in the composition of extracellular vesicles originating from different biological fluids. Secondly, the differential expression of CD144 in extracellular vesicles may reflect distinct physiological or pathological conditions associated with the sampled tissues or fluids.

As mentioned, the CD144 protein plays a critical role in endothelial cell adhesion and maintenance of vascular integrity. Endothelial cells continuously undergo turnover, where old or damaged cells are shed and replaced by new ones. The CD144 protein present on the surface of endothelial cells may be released into the bloodstream during this process. Therefore, these shed CD144 proteins can then be detected in serum samples.^63–65^ Besides, extracellular vesicles are small membrane-bound particles released by various cell types, including endothelial cells, into the extracellular space. CD144 has been identified as one of the proteins present in extracellular vesicles released by endothelial cells. Therefore, CD144 can be detected in both saliva and serum samples as a biomolecular cargo of extracellular vesicles released by endothelial cells.

Another explanation is that while endothelial cells primarily line blood vessels, they are also present in the microvasculature of various organs, including salivary glands. Endothelial cells within the salivary glands may express CD144; thus, trace amounts of CD144 may be present in saliva due to its secretion from these glands.^66,67^ Moreover, this explains the higher concentration of CD144 protein in serum than in saliva.

### Functionalization and detection of extracellular vesicles

Functionalizing Au-SPEs with MUA and antibodies significantly enhances their performance in electrochemical sensing applications. Initially, the Au-SPEs exhibit a resistance of 3.768 kΩ in their Au bare. After modification with MUA, the resistance increases to 33.95 kΩ, indicating a successful and dense MUA monolayer formation.^42,43^ This tenfold rise in resistance suggests that MUA effectively binds to the electrode surface, creating an insulating barrier that impedes charge transfer. The carboxylic acid groups in MUA serve as anchor points for immobilizing biomolecules, enabling the development of a bioactive surface for molecular recognition. Subsequent functionalization with the CD144 antibody further modifies the electrode. After antibody immobilization, the resistance decreases to 10.42 kΩ. This reduction indicates the successful binding of the antibodies to the MUA-modified surface, forming a bioactive layer capable of recognizing and capturing CD144 molecules.^68,69^

**Scheme 1:**
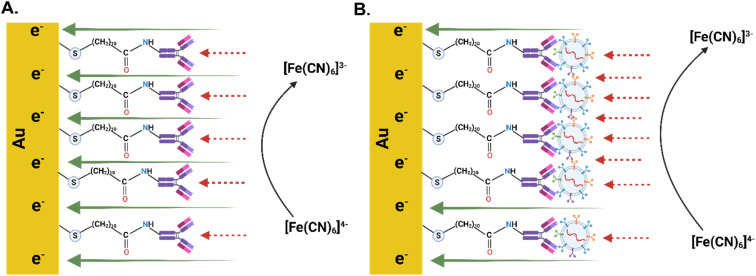
(A) Schematic diagram of the IgG-modified electrode. (B) Schematic diagram of the IgG-modified electrode with CD144-positive EV-Sal.

The decrease in resistance may be attributed to the introduction of new functional groups or changes in surface properties due to the antibodies, which could enhance surface conductivity and improve charge transfer kinetics. The MUA molecules form a self-assembled monolayer (SAM) on the gold electrode surface, a highly ordered molecular structure where molecules are oriented specifically. This SAM acts as an insulating barrier between the electrode and the surrounding electrolyte, further increasing resistance by hindering the flow of charge carriers.^70,71^ Antibodies, being protein molecules with charged and polar regions, may introduce additional functional groups when immobilized onto the MUA-functionalized surface. This can alter the surface properties, potentially lowering resistance by enhancing surface conductivity and facilitating better charge transfer.^72,73^ Functionalizing Au-SPEs with MUA and antibodies increases the electrodes' resistance, indicating successful surface modification, and creates a bioactive surface that effectively captures target molecules. The IgG antibodies are immobilized on the sensor surface and specifically recognize antigens present on the membrane of EVs. Upon introduction, the EVs bind to the Fab region of the IgG through antigen–antibody interactions. This specific binding enables the capture of EVs *via* the CD144 protein for downstream detection. This process enhances the electrodes' sensitivity and specificity in detecting biomarkers like CD144, making it a valuable approach in electrochemical sensing. Scheme 1 shows the mechanism proposed by the authors for the detection of CD-144 positive EV-Sal.

Using AFM to analyze gold screen-printed electrodes functionalized with the CD144 antibody represents an orthogonal approach to detecting and characterizing saliva-derived extracellular vesicles. This method offers several advantages over traditional techniques: (1) high sensitivity: AFM provides high-resolution imaging and precise measurement of nanoscale structures, enabling the detection of min differences in vesicle size and morphology. The functionalization of electrodes with the CD144 antibody enhances specificity, ensuring that only EVs carrying this protein are captured and detected. (2) Non-destructive analysis: unlike other fixation methods that utilize formaldehyde, which can alter the morphology of biological samples, AFM analysis does not require such chemical fixatives. This preserves the native structure of the EVs, providing more accurate and reliable data on their proper morphology and size. (3) New detection platform: the combination of gold screen-printed electrodes and AFM presents a new platform for EV detection and characterization, managing EV heterogeneity, which is a significant need in the EV field. Gold electrodes provide a conductive and biocompatible surface that can be easily functionalized with antibodies, making this method adaptable and scalable for various types of EV analysis.^74,75^

A key finding from this study is the preservation of the EVs' morphological integrity when we immune adsorbed the EVs on the surface without changing the DPBS buffer to another chemical environment.^76–78^ The detected vesicles had sizes of 182 nm and 116 nm and heights of 20.5 nm and 15 nm.^79^ Respectively, it demonstrates that the morphology of the EVs is not compromised during the detection process. This is particularly important as it confirms that the method employed does not introduce artifacts or distortions that could affect the interpretation of the data. The absence of formaldehyde in the preparation process ensures that the vesicles retain their native shape and structural properties. This non-destructive approach is crucial for studies aiming to understand EVs' biological functions and clinical relevance, as any alteration in their morphology could impact their biological activity and the accuracy of downstream analyses.

The successful application of this AFM-based method for detecting saliva-derived EVs functionalized with the CD144 antibody opens new avenues for research and clinical diagnostics. The ability to accurately measure and characterize EVs without compromising their integrity is essential for advancing our understanding of their role in physiological and pathological processes.^18,80^

The application of AFM, particularly with 3D topography imaging, to analyze gold screen-printed electrodes functionalized with the CD144 antibody represents a novel and advanced approach to detecting and characterizing EVs. The 3D topography images provide a comprehensive view of the EVs' morphology, enabling a more detailed analysis of their size and shape. This enhanced visualization is handy for confirming the integrity and uniformity of the captured vesicles. The uniform distribution and well-preserved morphology of the EVs on the functionalized electrodes confirm that the detection process did not introduce any artifacts or distortions. This is particularly important for downstream applications, where the accurate representation of EV morphology can impact their functional analysis and potential use as biomarkers.

### Calibration curve of extracellular vesicles from human saliva

The development of a calibration curve for CD144-positive extracellular vesicles underscores this method's high sensitivity. The CD144 protein is a well-established endothelial biomarker. Its presence on extracellular vesicles can provide significant insights into endothelial health and disease states.^81–83^ The LOQ of 0.37 ng mL^−1^ highlights the method's ability to detect deficient concentrations of CD144, which is crucial for early detection and monitoring of endothelial-related conditions. The method's sensitivity is further exemplified by its ability to quantify up to 1.11 pg of CD144-positive EV-Sal. This high sensitivity is critical for clinical applications where early and accurate detection of biomarkers can lead to better diagnosis and treatment outcomes.^84–87^

One notable advantage of this method is the minimal sample volume required. Only 3 μL of sample is needed for analysis, making this technique highly efficient and suitable for applications where sample availability is limited. This low volume requirement mainly benefits clinical settings and research involving rare or precious samples.

When compared to other existing methods for EV quantification, this method offers several distinct advantages: (1) enhanced sensitivity: traditional methods, such as enzyme-linked immunosorbent assay (ELISA) (see Table 2) or flow cytometry, often require larger sample volumes and may not achieve the same level of sensitivity.^88–91^ The ability to detect and quantify CD144-positive EVs at concentrations as low as 0.111 ng mL^−1^ and 0.37 ng mL^−1^, respectively, represents a significant improvement. (2) Reduced sample volume: techniques like western blotting and mass spectrometry generally require larger sample volumes and more extensive preparation steps.^92–95^ The minimal sample volume required for this calibration curve (3 μL) reduces sample preparation time and preserves more of the sample for other analyses. (3) Ease of use and scalability: the method's simplicity and scalability make it suitable for routine clinical diagnostics and large-scale research studies.^96–98^

**Table 2:** Comparison between methods

Detection method	Biomarkers	Sample volume	LOD
Biosensor (this work)	CD144 protein	3 μL	0.111 ± 0.01079 ng mL^−1^
ELISA	CD144 protein	50 μL	0.331 ng mL^−1^^a^****

a A *t*-test was performed to compare the detection limits of the biosensor with the detection limit provided by the ELISA kit vendor. Statistical significance: ****P value < 0.0001.

## Conclusions

The calibration curve for CD144-positive EV-Sal demonstrates a highly sensitive method for quantifying these endothelial biomarkers. With a limit of detecting of 0.111 ng mL^−1^, a limit of quantification of 0.37 ng mL^−1^, and the ability to quantify up to 1.11 pg, this method stands out for its sensitivity. Additionally, the minimal sample volume requirement of 3 μL enhances its practicality and efficiency compared to traditional methods such as the ELISA test, which has a limit of detection of 0.331 ng mL^−1^ and a limit of quantification of 0.63 ng mL^−1^. ELISA assay uses 50 μL samples for its tests. This approach offers significant potential for clinical diagnostics, therapeutic monitoring, and further biomarker research, making it a valuable tool in extracellular vesicle analysis. AFM confirmed the successful capture of CD144-positive EVs while preserving their morphology, establishing an efficient and reliable method for characterizing EVs in SAM systems on Au-SPEs.

## Data availability

The datasets supporting this article have been uploaded as part of the ESI.†

## Author contributions

Conceptualization, CM and LC; methodology, CM, LC, WMM; validation, CM, LL, LC, and WMM; formal analysis, CAM, LC, ES, DOF, DS, LL, and WMM; investigation, WMM; resources, CM; data curation, CM, LC, DOF, DS, LL and WMM; writing—original draft preparation, JJO, SAM, MJ, LL, ES, DOF, DS, CM and WMM; supervision, CM; project administration, CM; funding acquisition, CM.

## Conflicts of interest

There are no conflicts to declare.

## Supplementary Material

RA-015-D4RA08926J-s001
