# Supplementary material for: Functionalized screen-printed electrodes for non-invasive detection of vascular-endothelial cadherin in extracellular vesicles
Source: RSC Adv. 2025 Apr 22;15(16):12609–21. doi: 10.1039/d4ra08926j (PMC12012609; doi:10.1039/d4ra08926j)
Supplement: RA-015-D4RA08926J-s001 [file RA-015-D4RA08926J-s001.pdf]

Supplementary Information (SI) for RSC Advances.
This journal is © The Royal Society of Chemistry 2025

The supplementary information

Functionalized Screen-Printed Electrodes for Non-Invasive Detection of Vascular-Endothelial Cadherin in Extracellular Vesicles

William Meza-Morales[a], Sahimy Ayus-Martinez[a], Jesus Jimenez-Osorio[a], Maria Buendia-Otero[a], Luis López[b], David Suleiman[a], Edu Suarez[c], Donald O. Freytes[d], Lisandro Cunci[b], and Camilo Mora[a*]

a. Department of Chemical Engineering, University of Puerto Rico-Mayaguez, Route 108, Mayaguez, Puerto Rico, USA.
b. Department of Chemistry, University of Puerto Rico-Rio Piedras, 601 Av. Universidad, San Juan, Puerto Rico, USA.
c. Department of Biology, University of Puerto Rico-Ponce, Av. Santiago de los Caballeros, Ponce, Puerto Rico, USA.
d. The Joint Department of Biomedical Engineering, North Carolina State University/University of North Carolina at Chapel Hill, 4130 Engineering Building III, Campus Box 7115, Raleigh, NC 27695, USA.

* Corresponding author: camilo.mora@upr.edu

Methodology for isolating EVs from saliva provided by the supplier

To isolate EVs from saliva (EV-Sal) using the ExoQuick® system, first, collect saliva samples and centrifuge them at 2,000 × g for 10 minutes at 4°C to remove cells and debris. Transfer the supernatant to a new tube and add ExoQuick solution at the manufacturer-recommended volume (typically a 1:5 ratio of ExoQuick to saliva). Mix the solution by inverting the tube several times and incubate it at 4°C for 30 minutes to allow EV precipitation. Next, centrifuge at 1,500 × g for 30 minutes at 4°C, carefully remove the supernatant, and resuspend the EV pellet in PBS or an appropriate buffer.

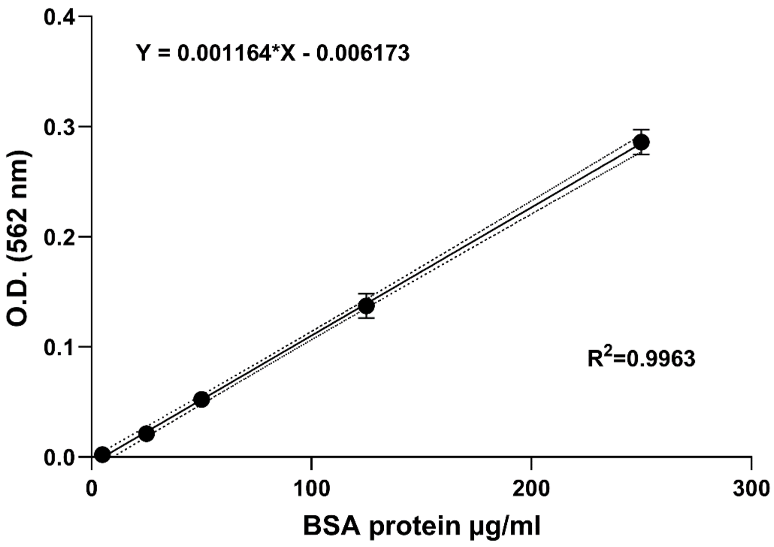

Figure S1. Calibration curve of protein quantification.

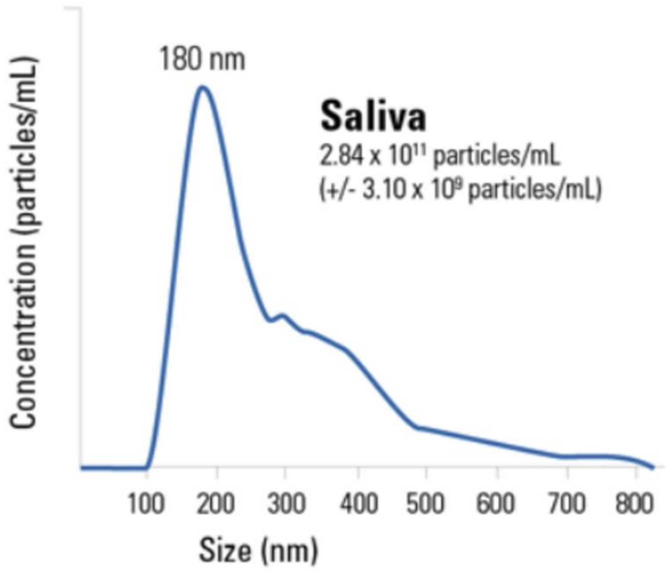

Figure S2. EV-Sal NTA provided by SBI supplier.

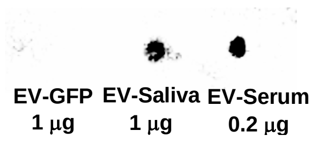

Figure S3. Dot Blot tests of CD144 proteins for EV-GFP, EV-Sal, and EV-Serum.

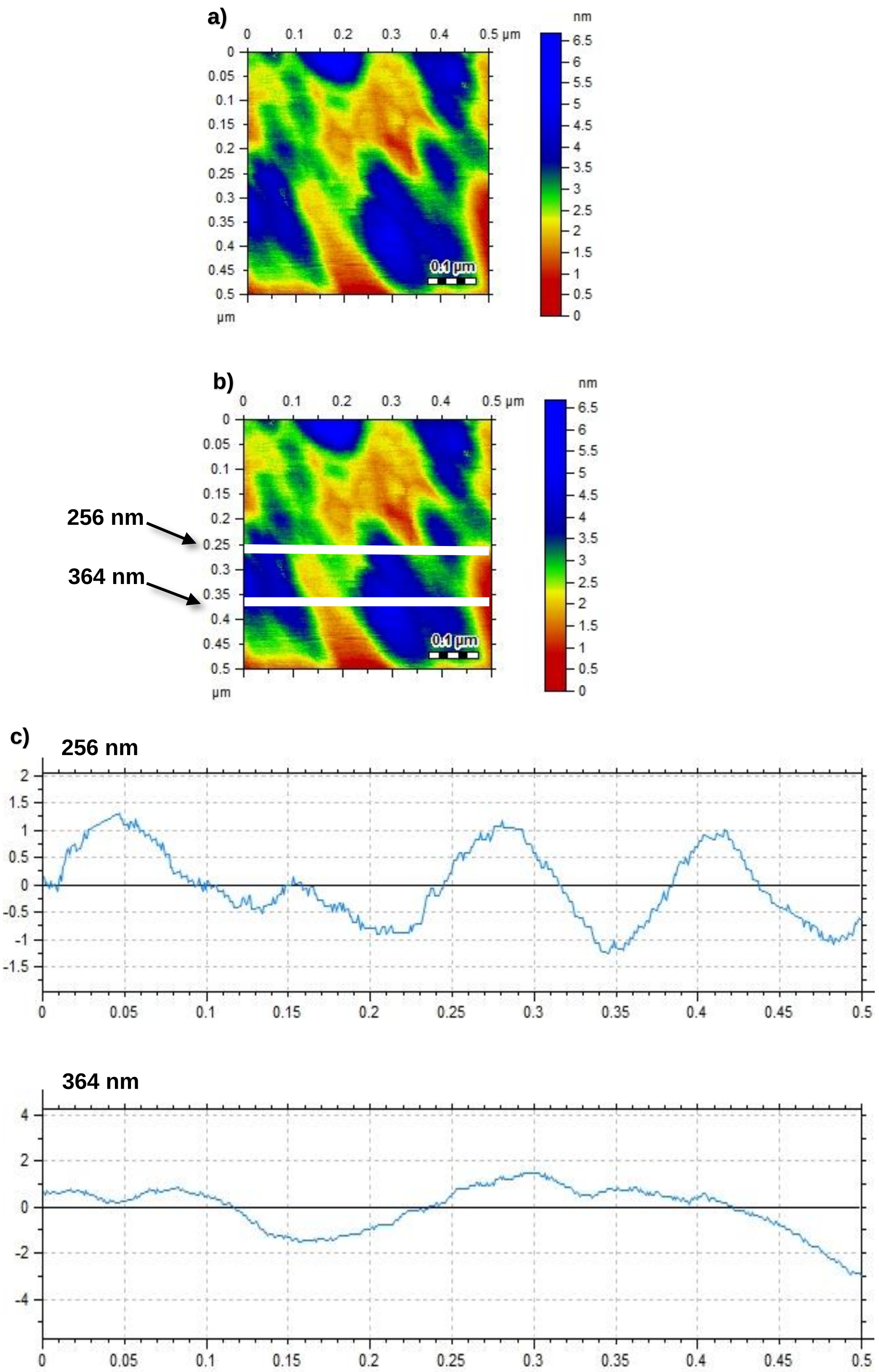

Figure S4. **A**. AFM topography image of the biosensor with CD144 antibody. **B**. Site where the height analyses were carried out. **C**. Height measurement analysis of biosensor with CD144 antibody on AFM topography image.

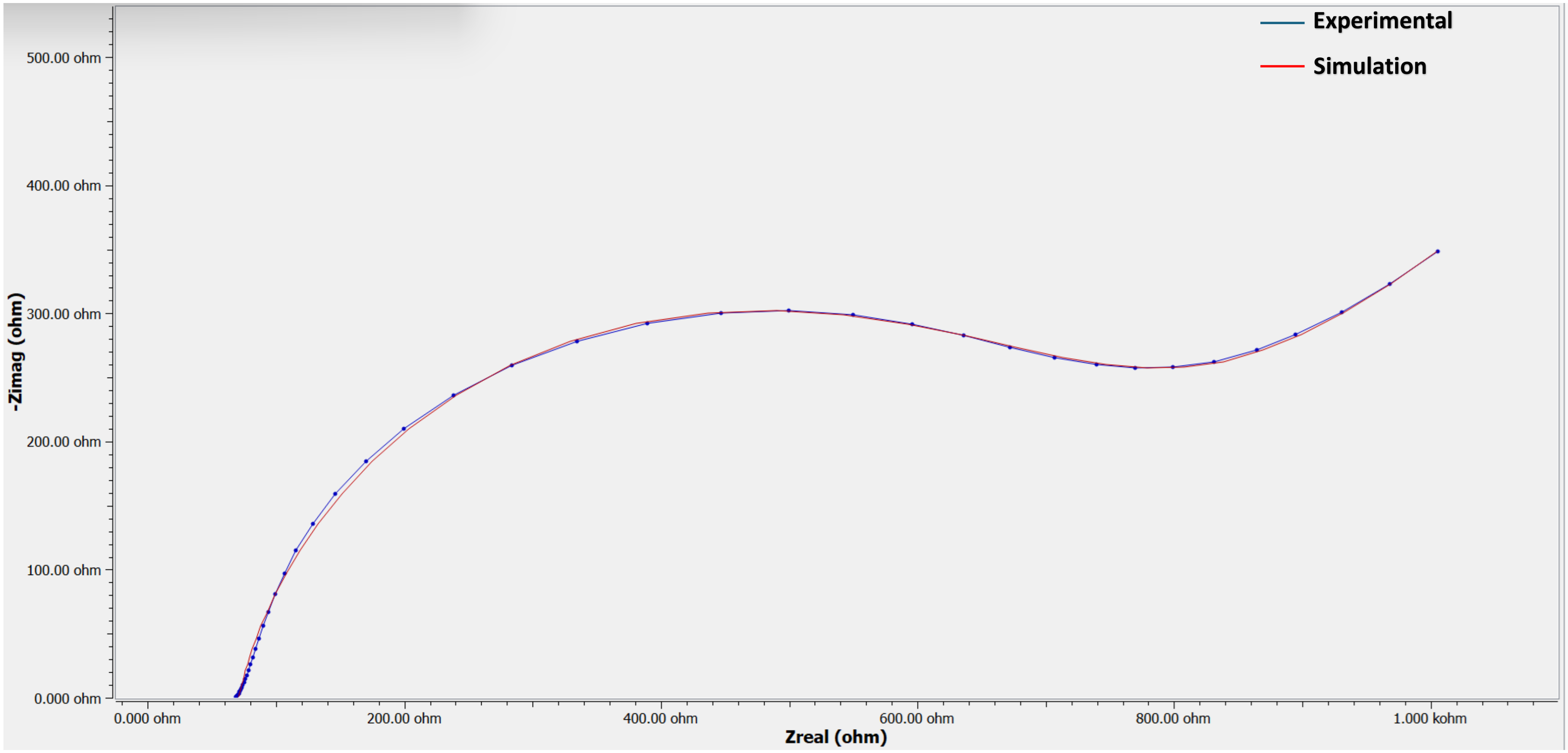

Figure S5. EIS of gold bare E1.

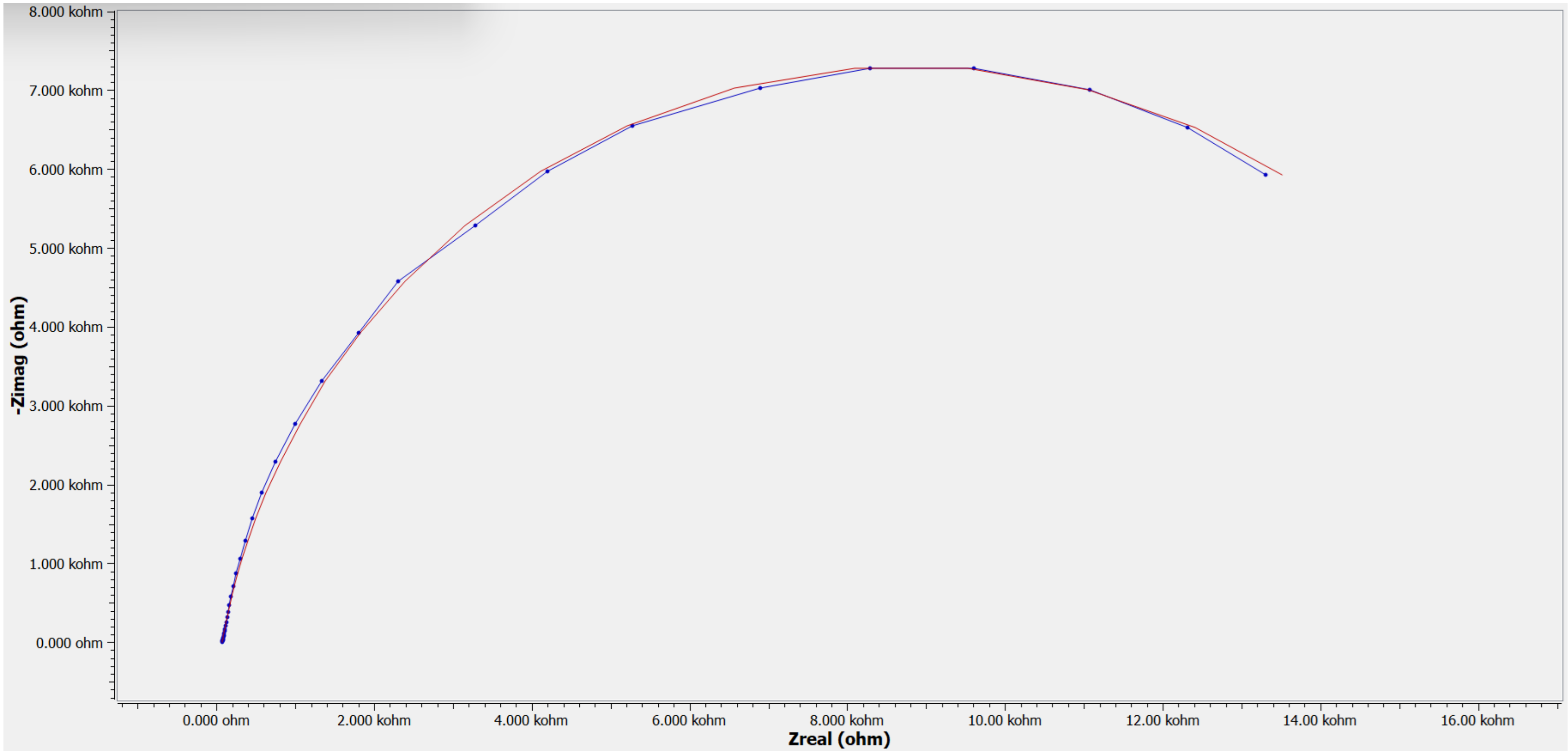

Figure S6. EIS of gold-MUA E1.

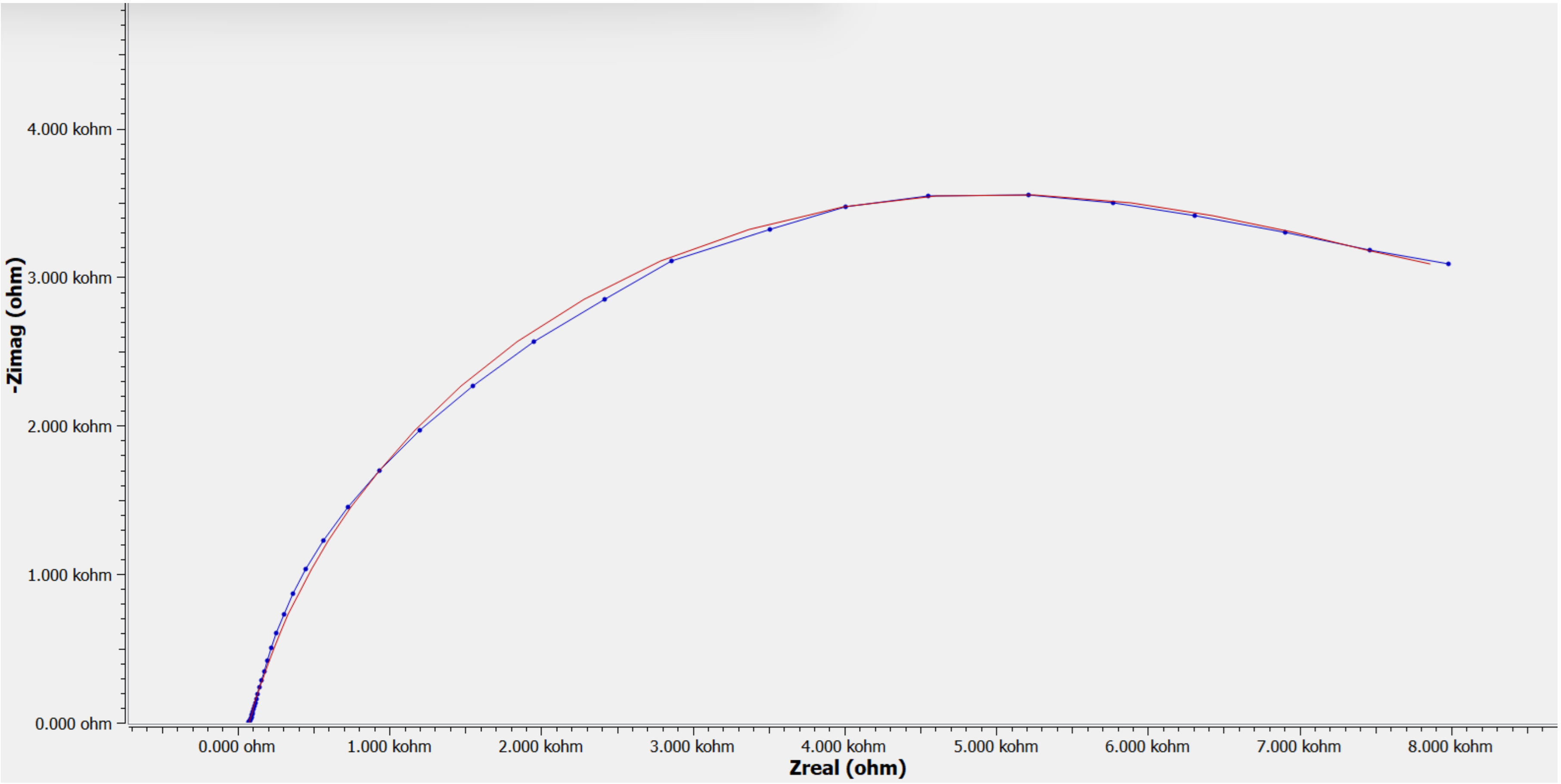

Figure S7. EIS of gold-MUA-CD144AB E1.

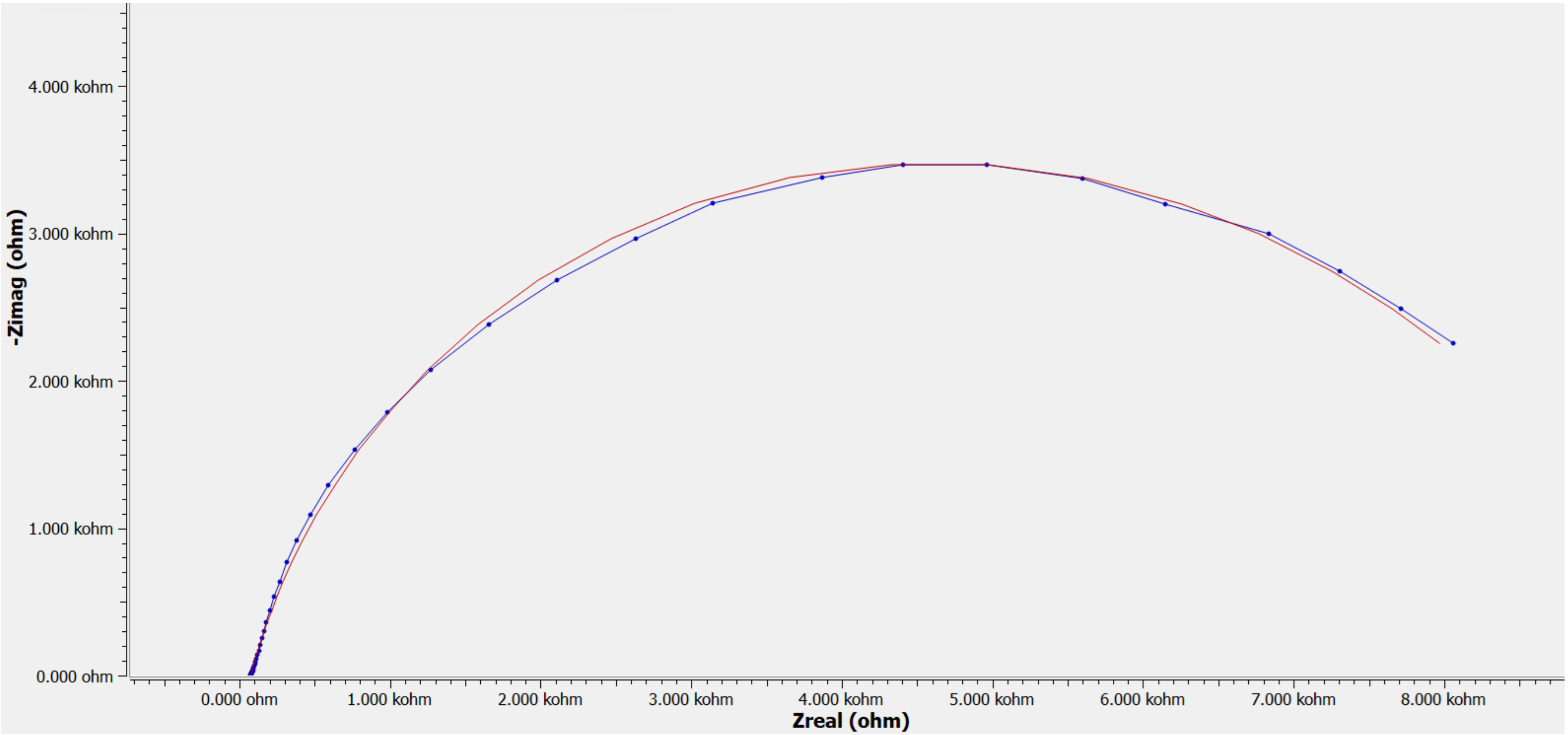

Figure S8. EIS of gold-MUA-CD144AB-EV(std. 1) E1.

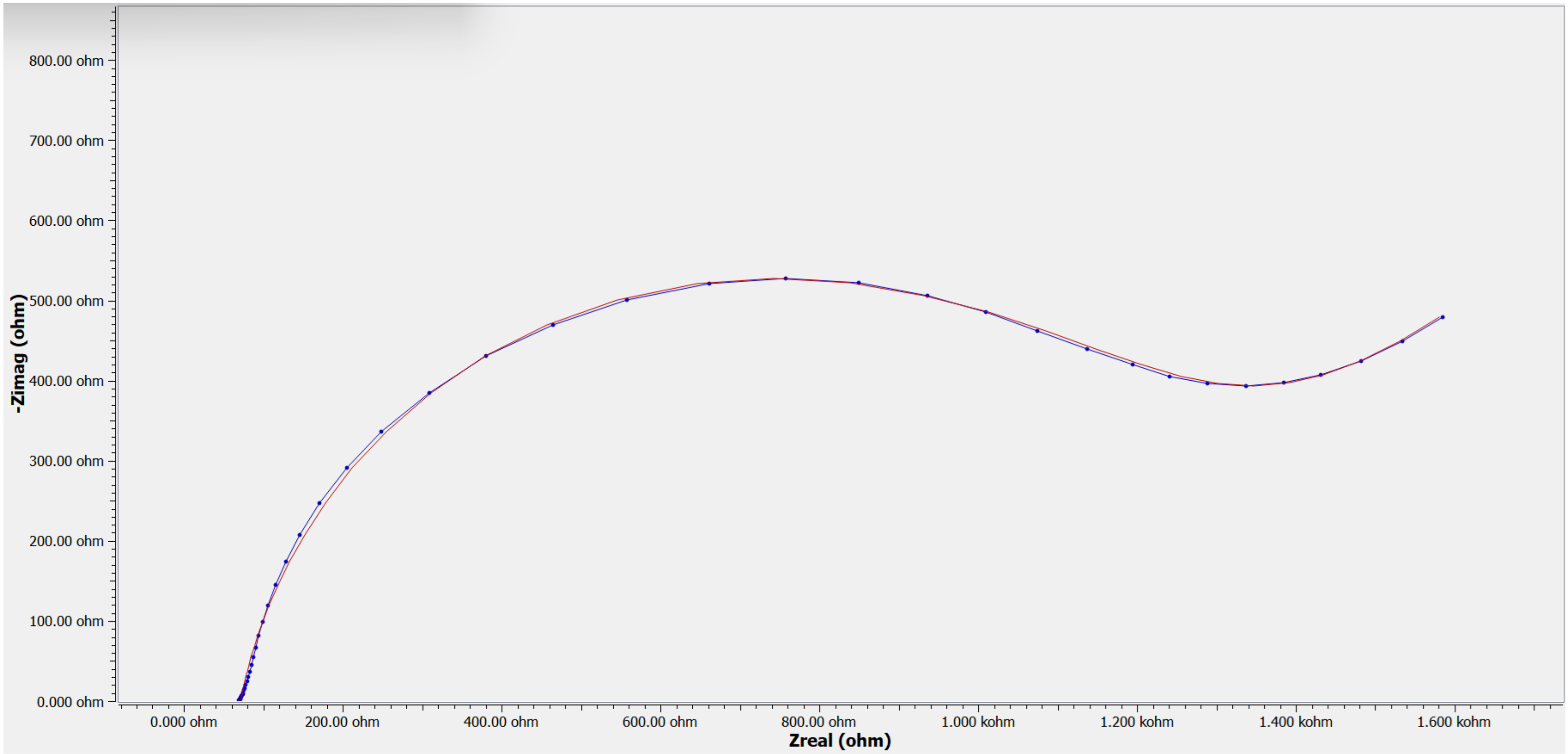

Figure S9. EIS of gold bare E2.

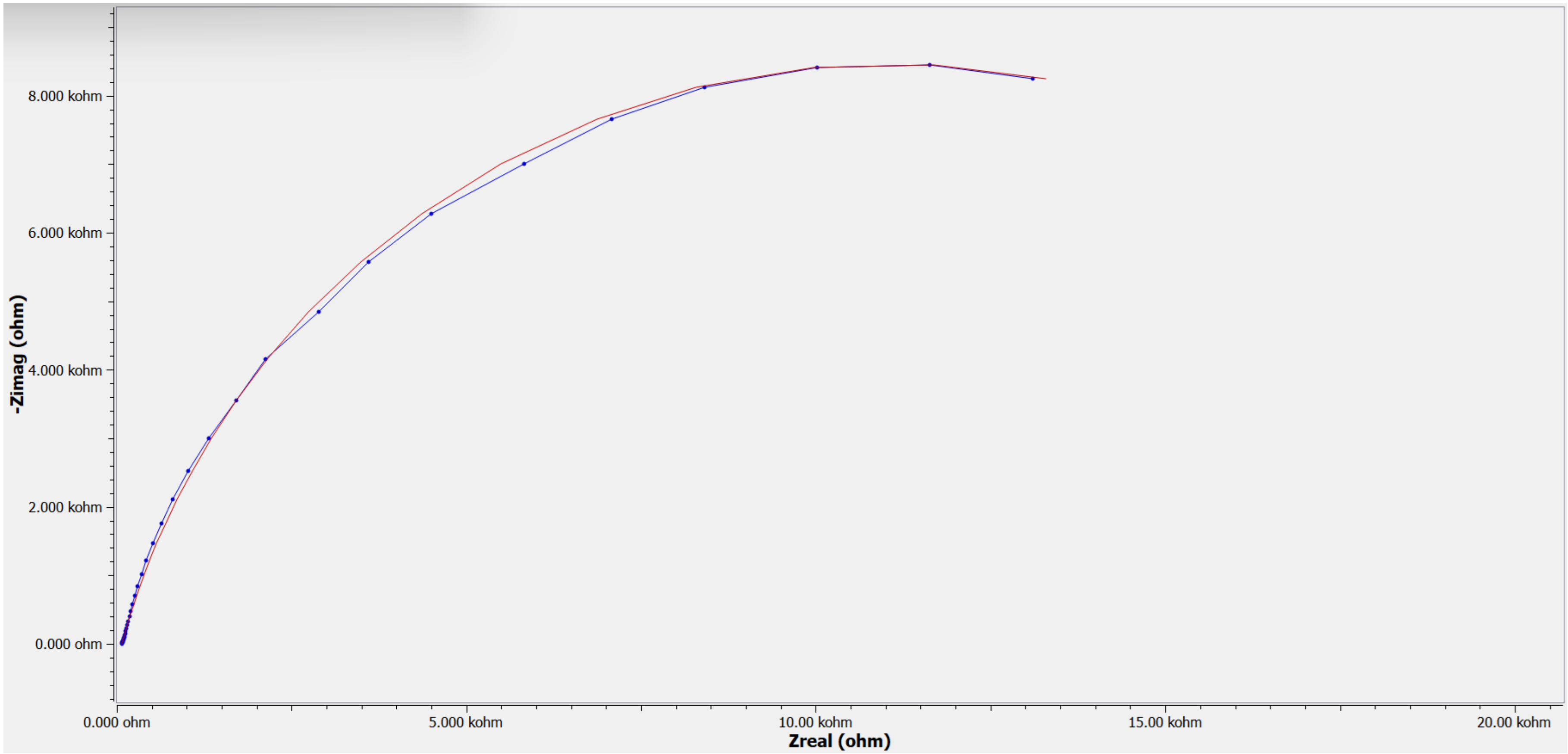

Figure S10. EIS of gold-MUA E2.

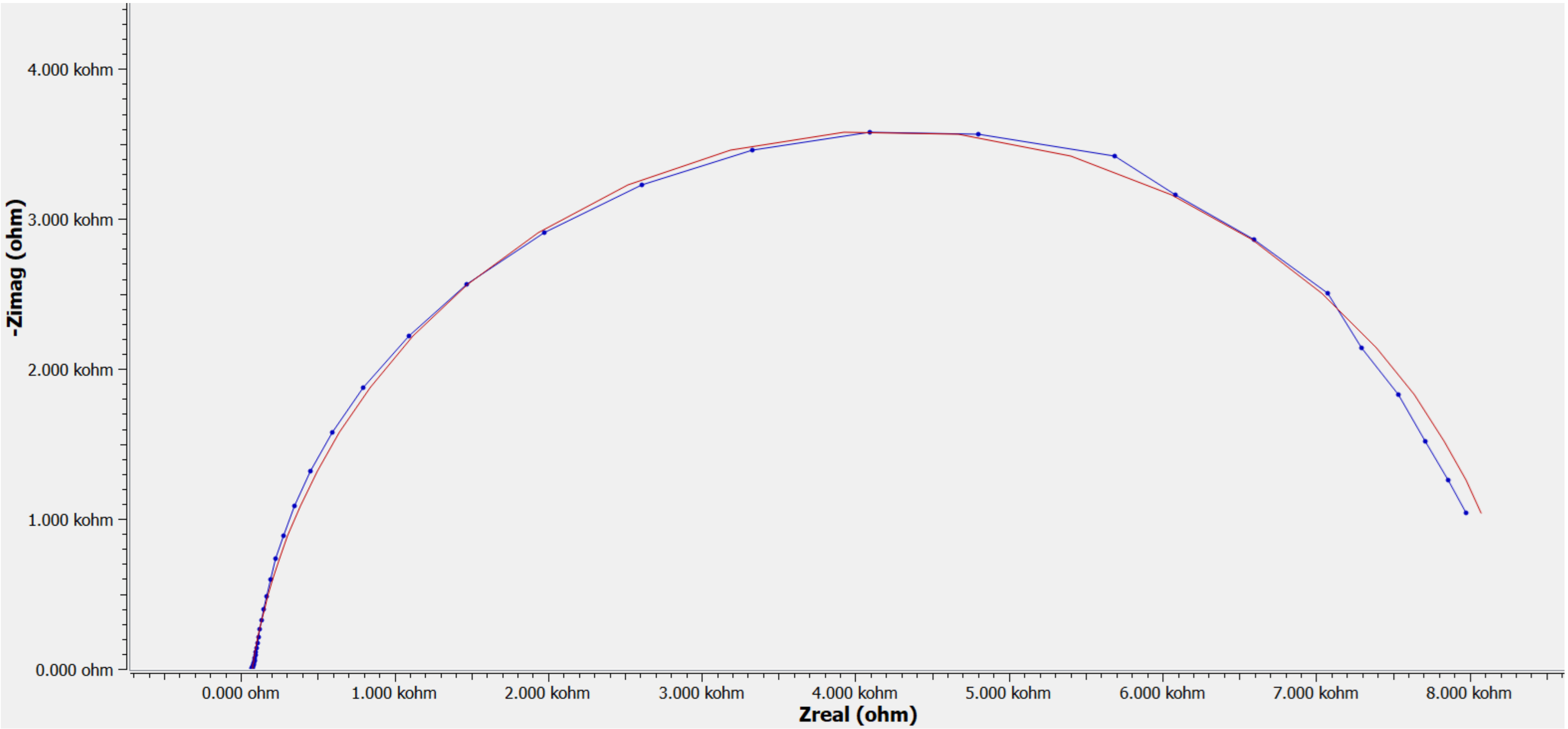

Figure S11. EIS of gold-MUA-CD144AB E2.

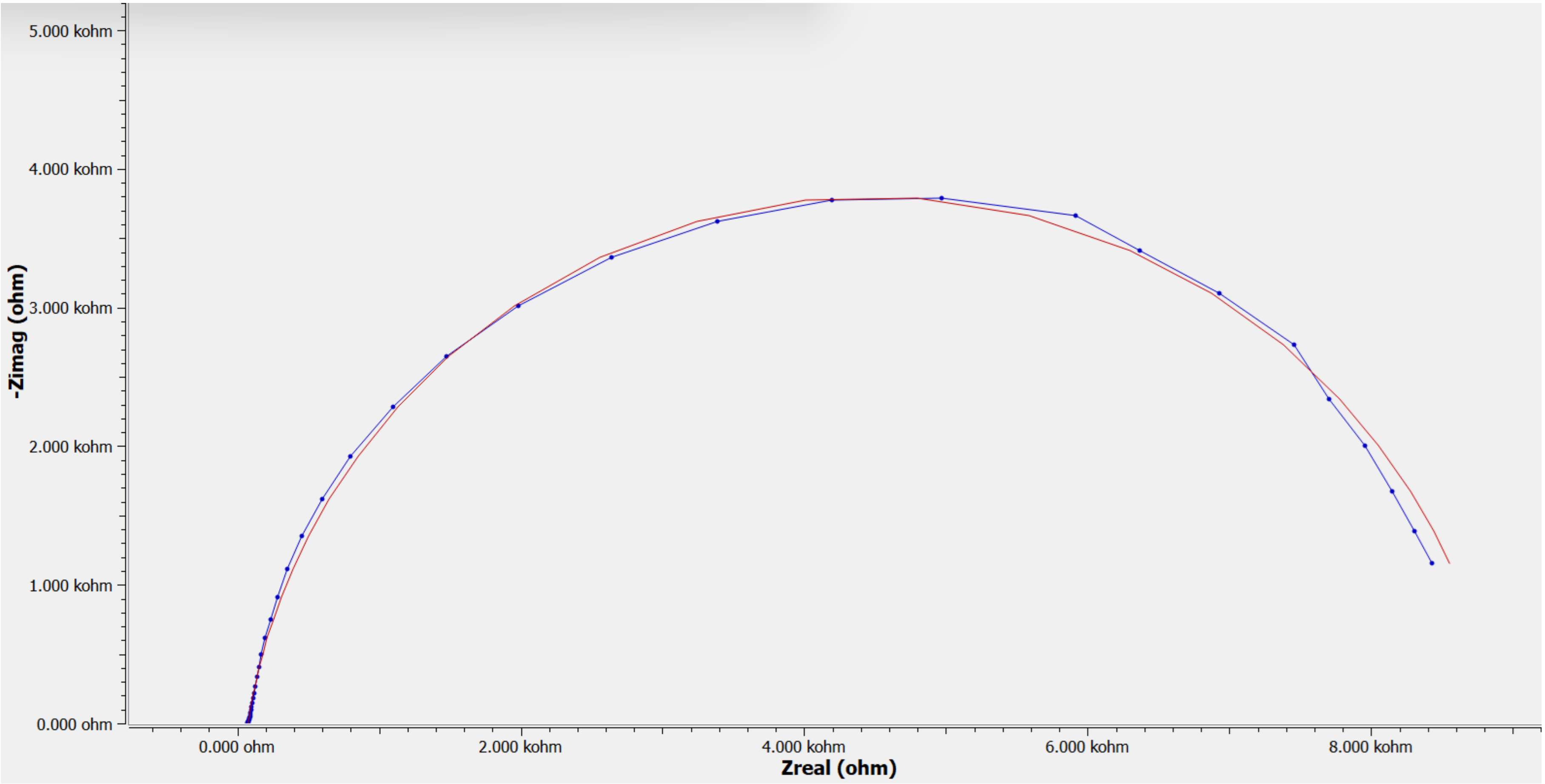

Figure S12. EIS of gold-MUA-CD144AB-EV(std. 1) E2.

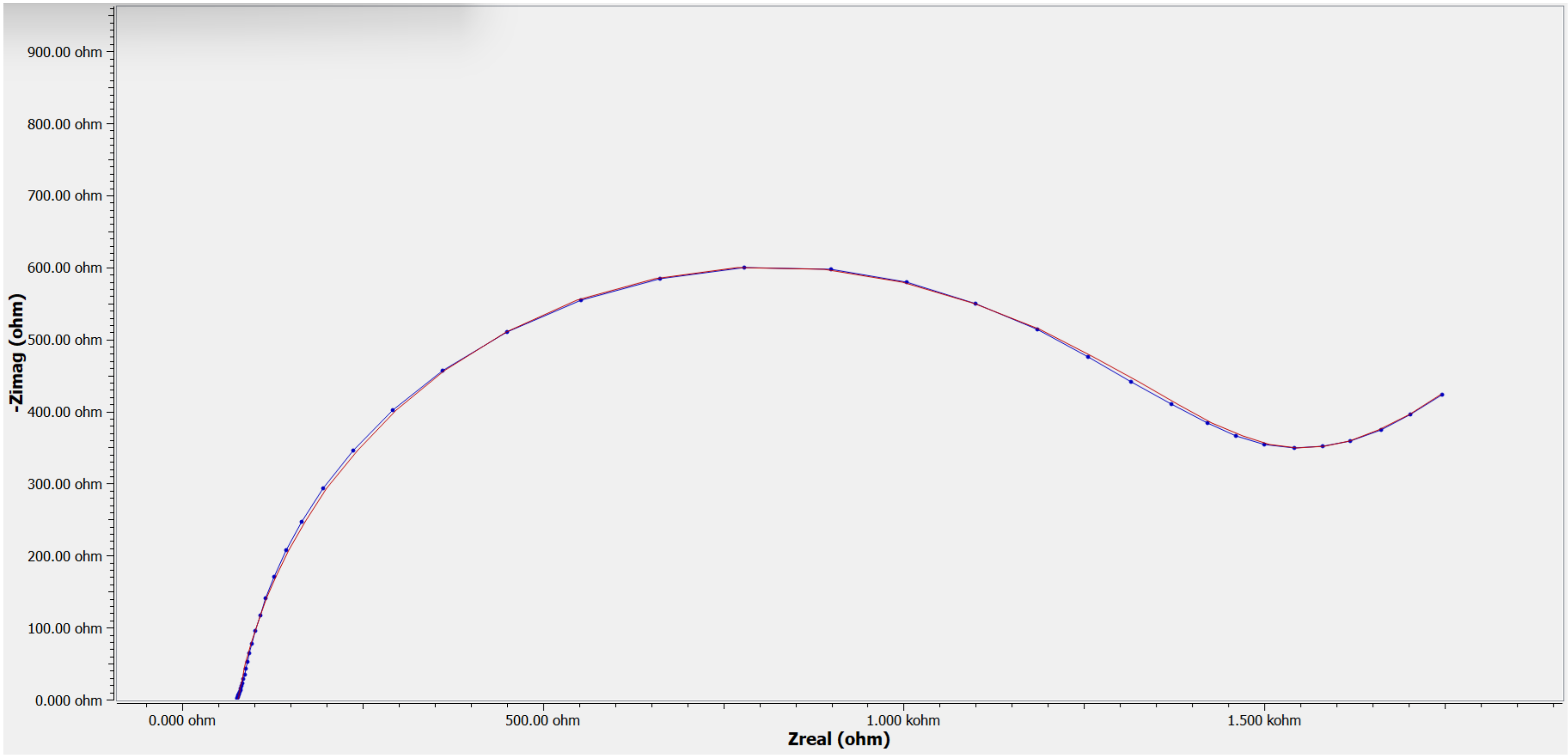

Figure S13. EIS of gold bare E3.

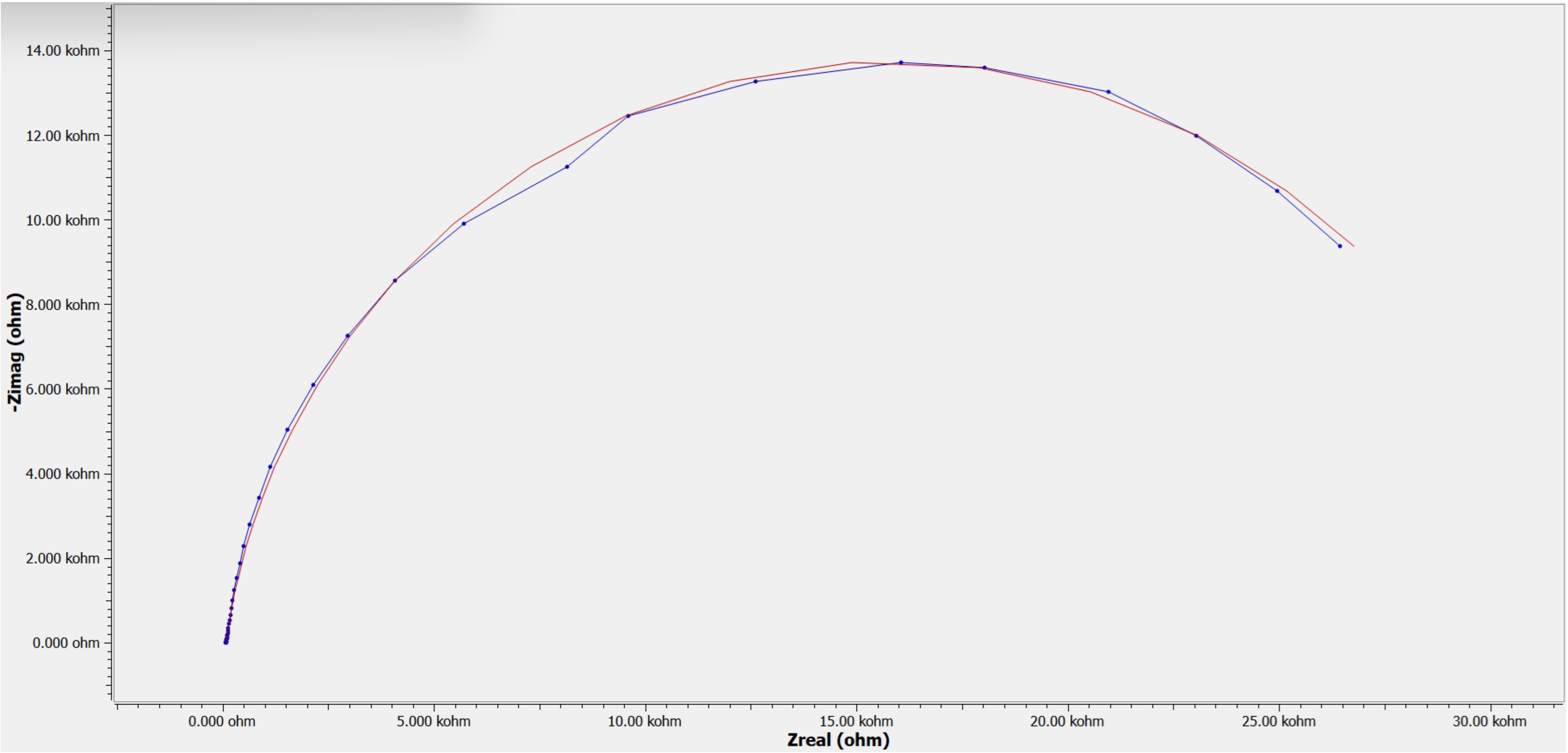

Figure S14. EIS of gold-MUA E3.

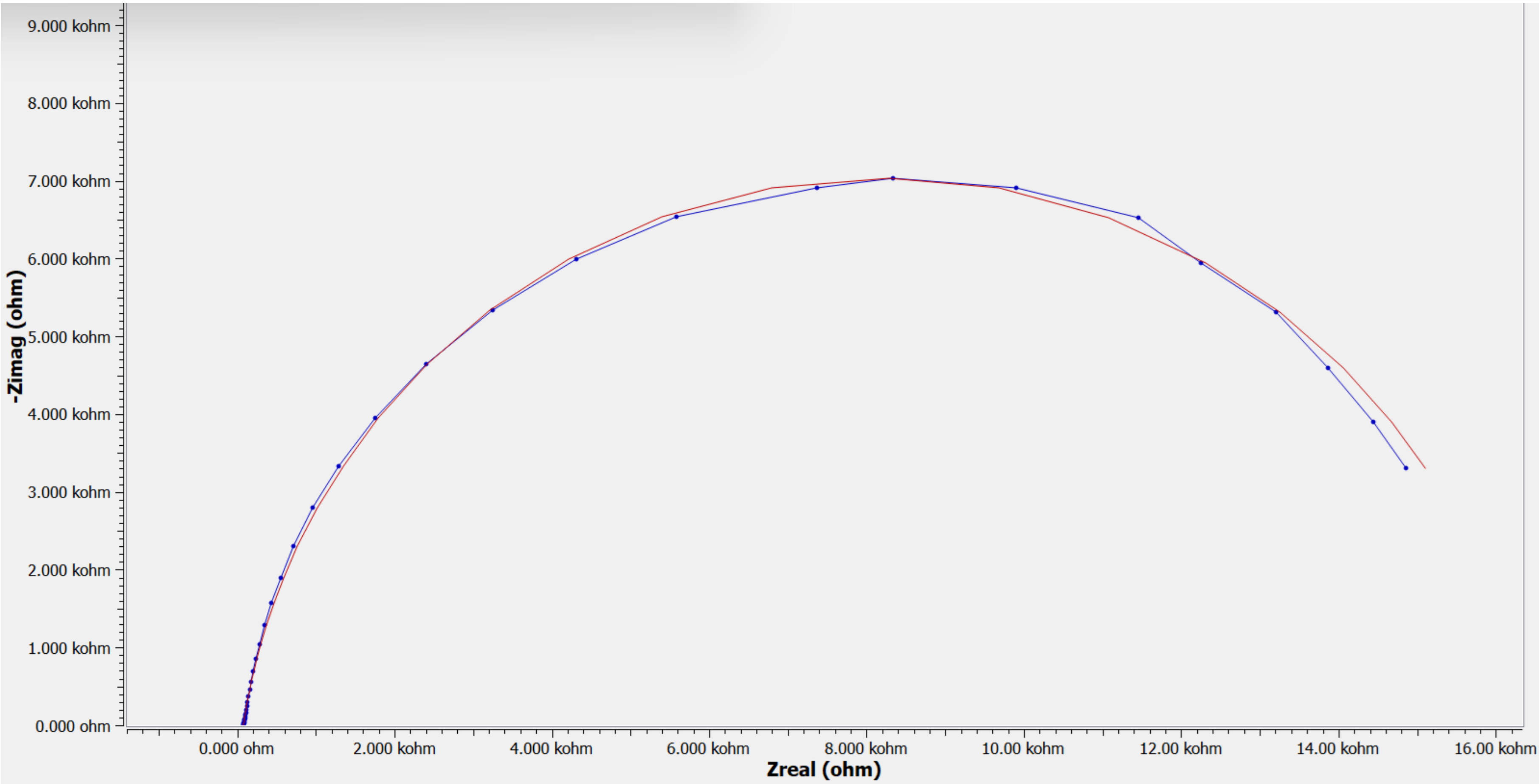

Figure S15. EIS of gold-MUA-CD144AB E3.

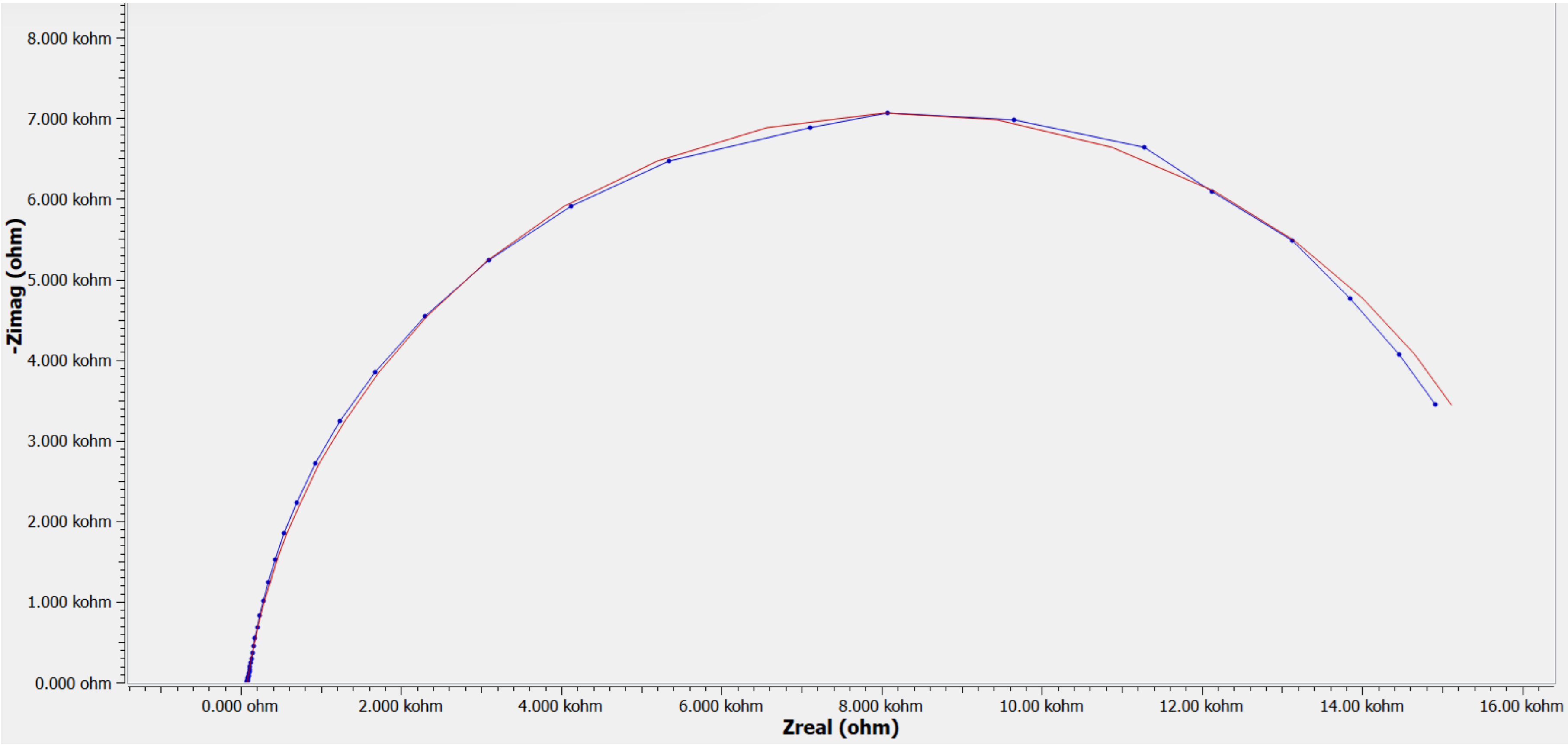

Figure S16. EIS of gold-MUA-CD144AB-EV(std. 1) E3.

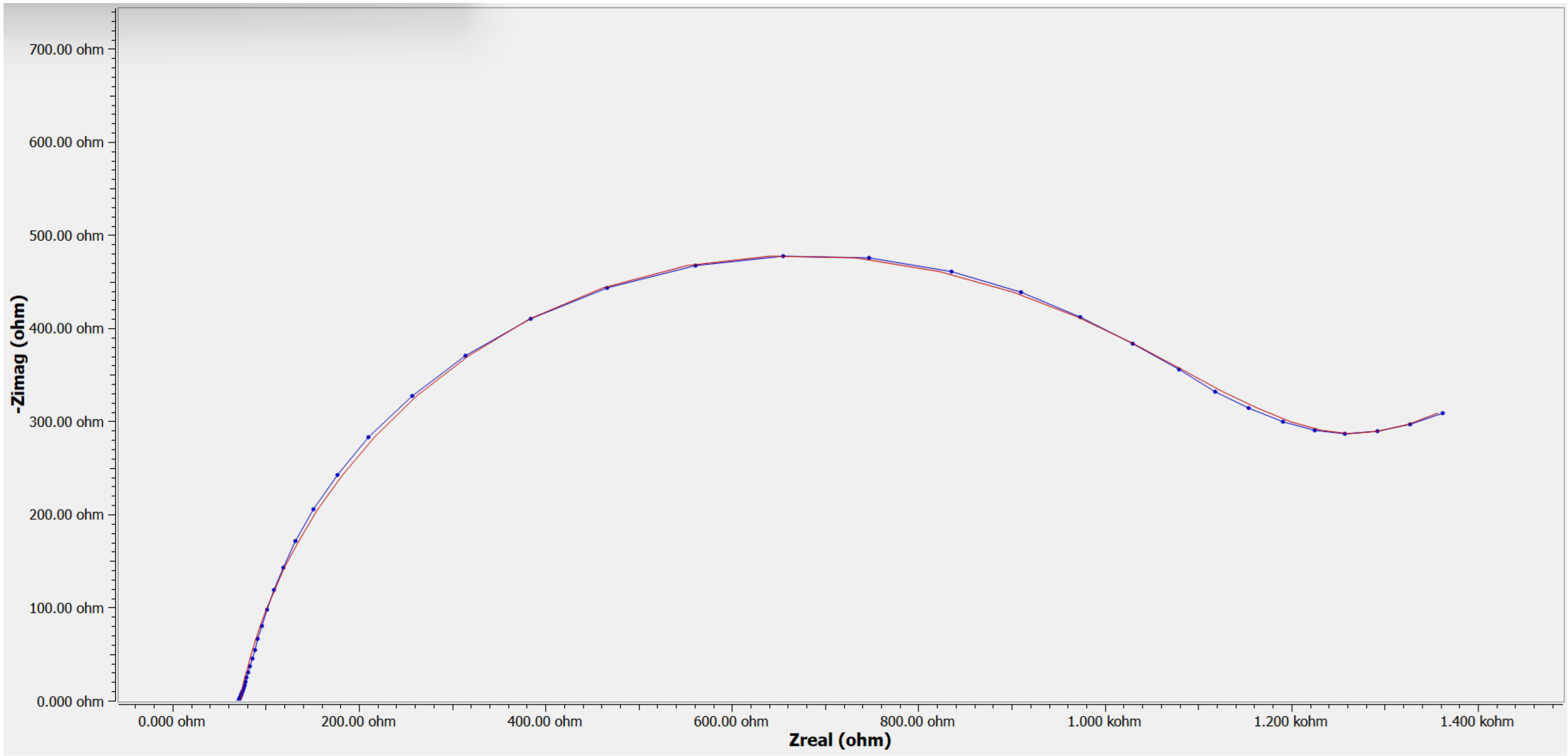

Figure S17. EIS of gold bare E4.

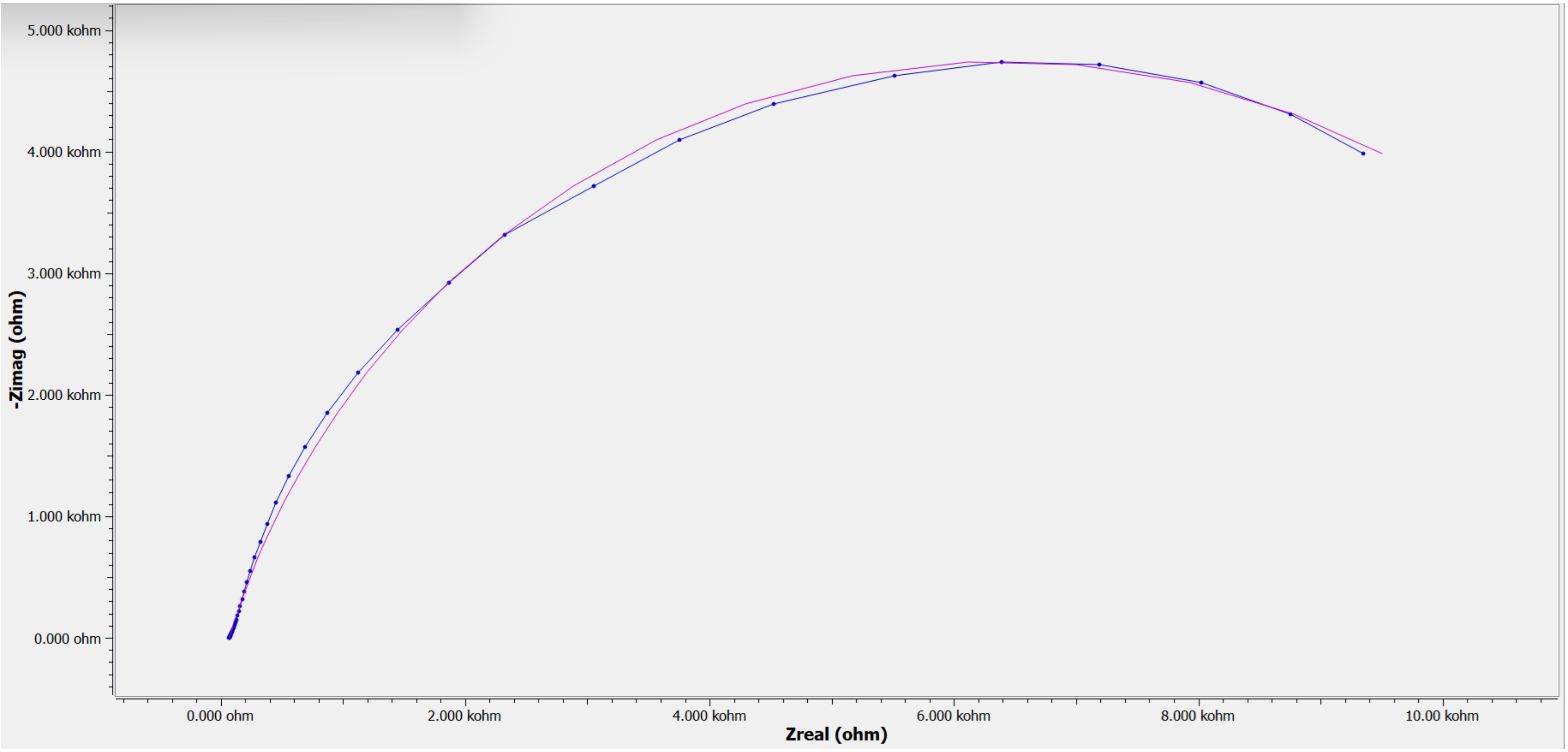

Figure S18. EIS of gold-MUA E4.

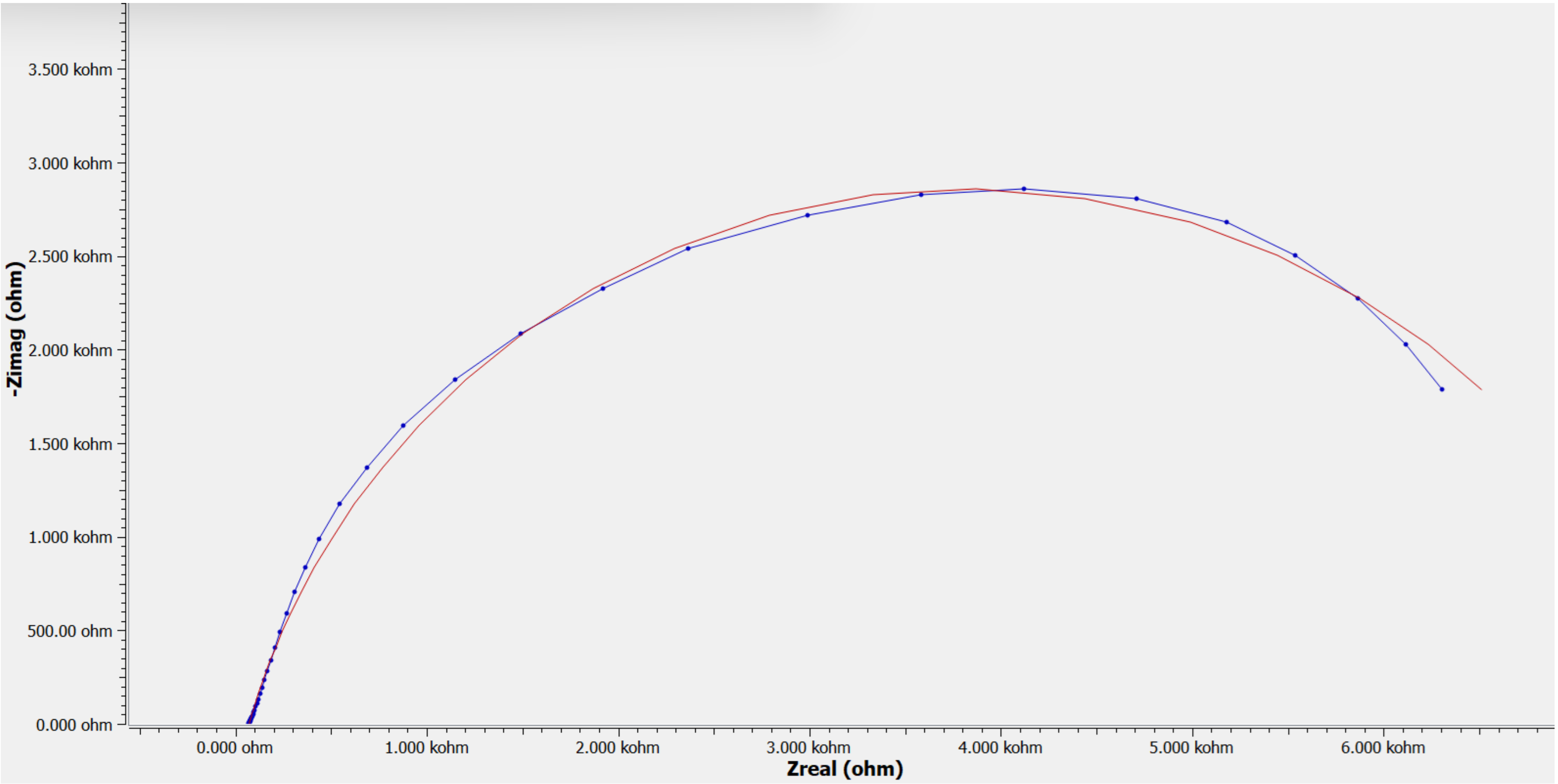

Figure S19. EIS of gold-MUA-CD144AB E4.

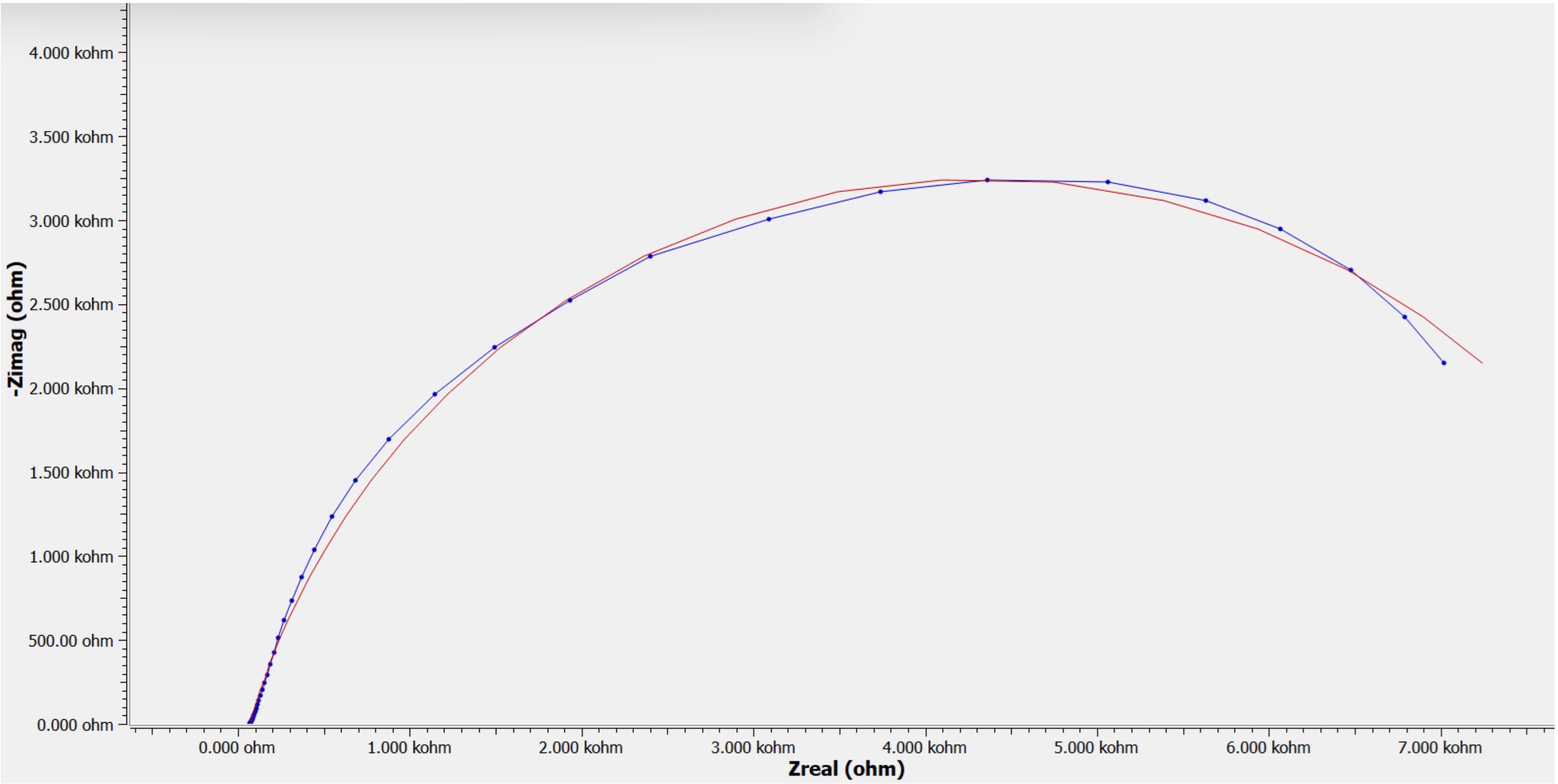

Figure S20. EIS of gold-MUA-CD144AB-EV(std. 2) E4.

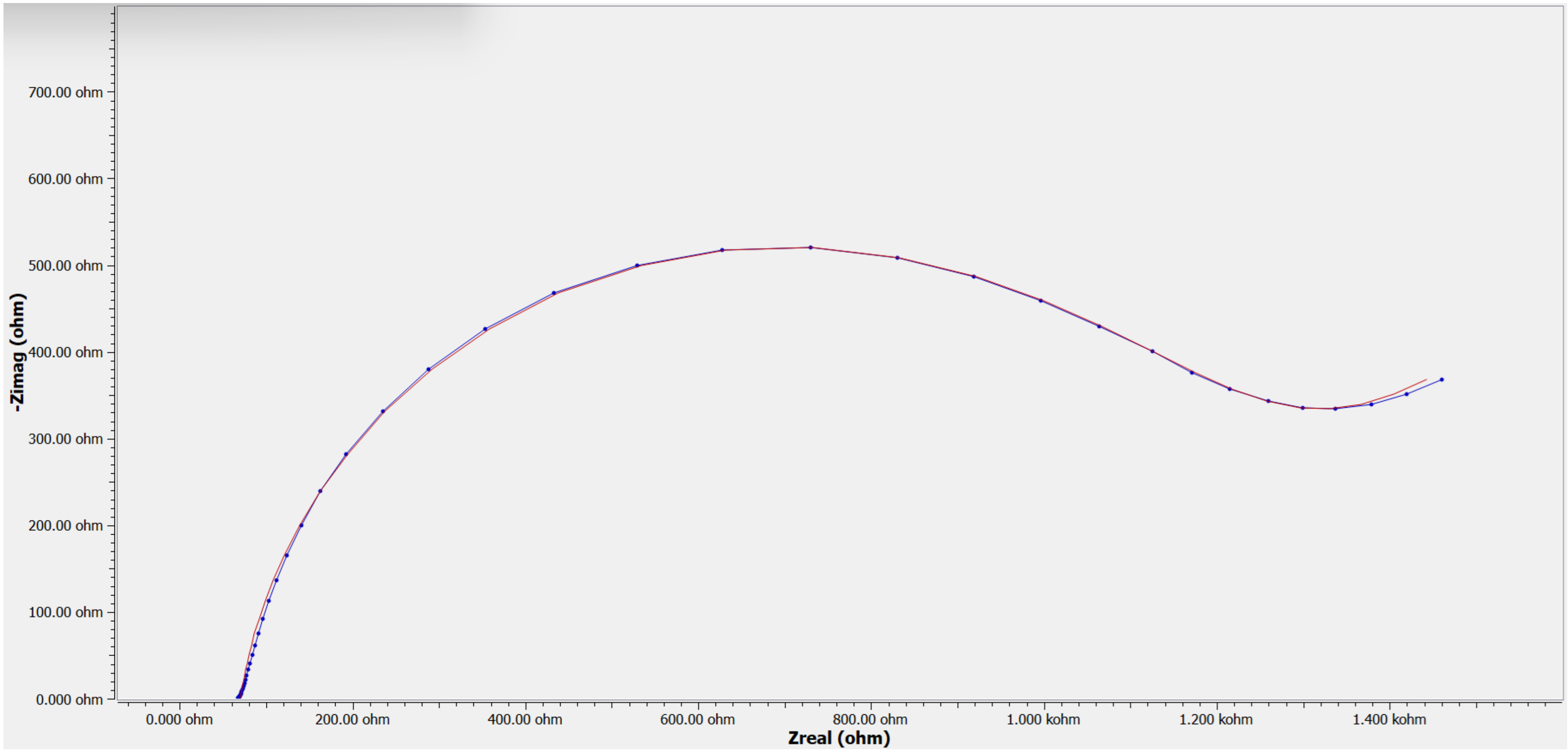

Figure S21. EIS of gold bare E5.

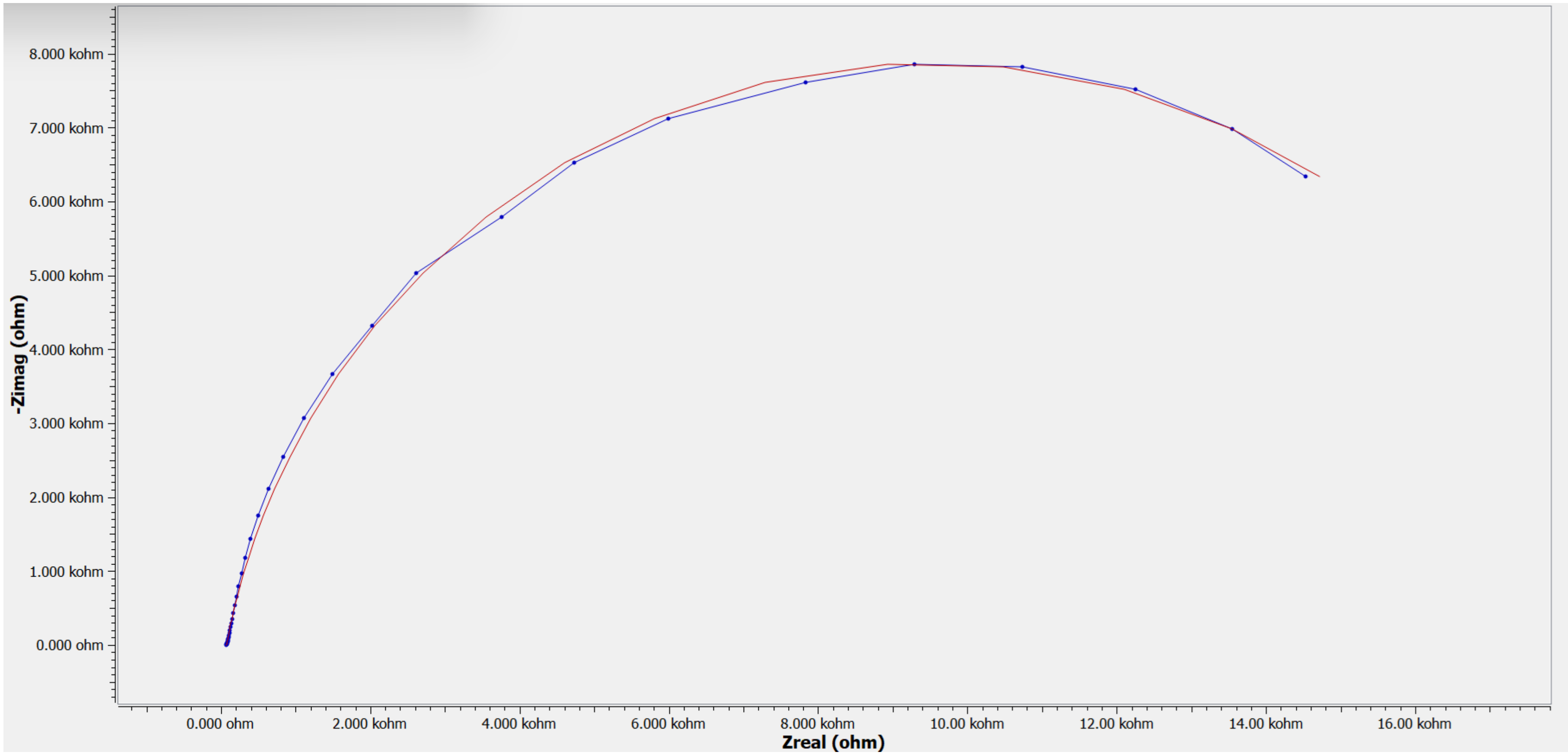

Figure S22. EIS of gold-MUA E5.

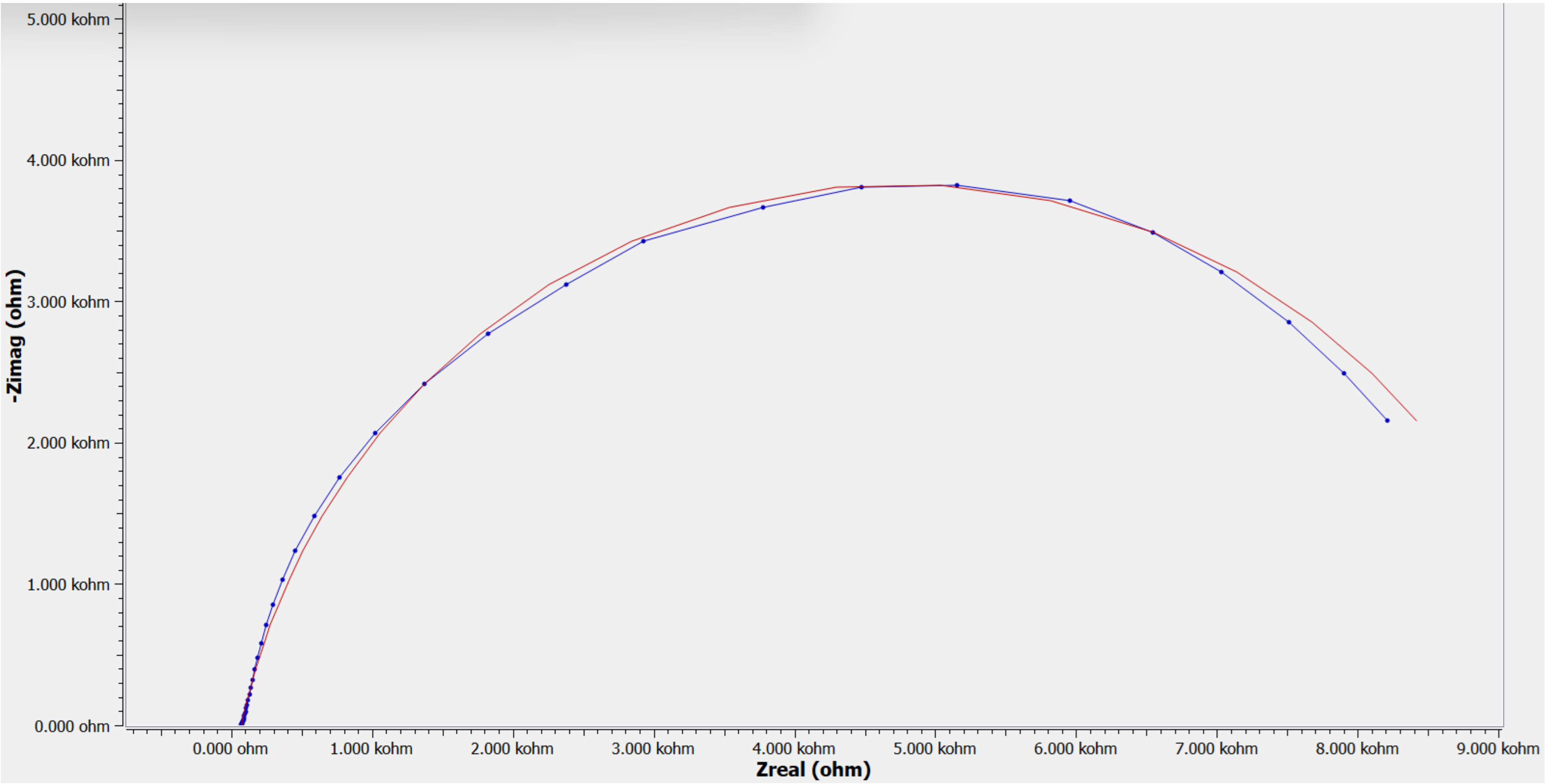

Figure S23. EIS of gold-MUA-CD144AB E5.

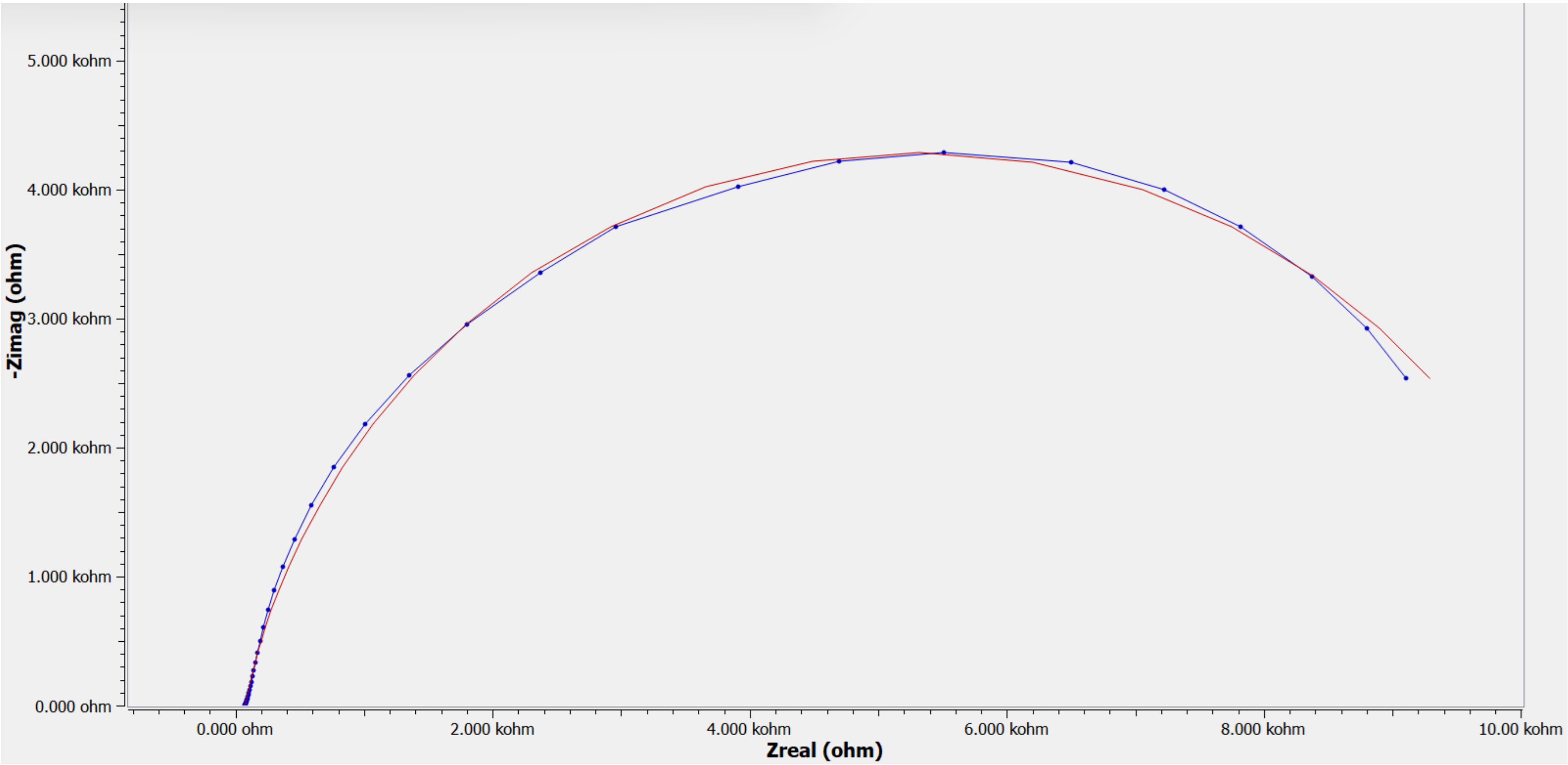

Figure S24. EIS of gold-MUA-CD144AB-EV(std. 2) E5.

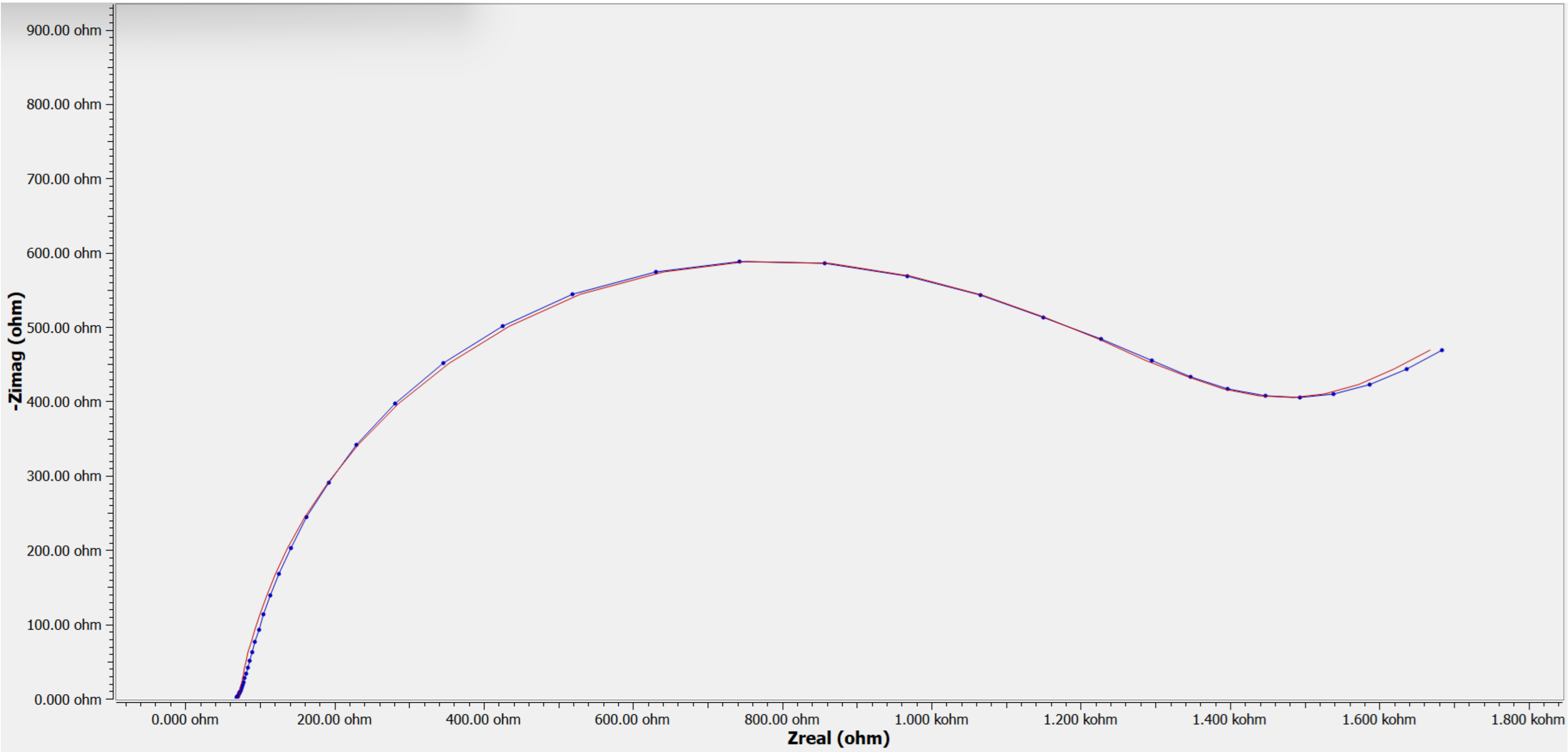

Figure S25. EIS of gold bare E6.

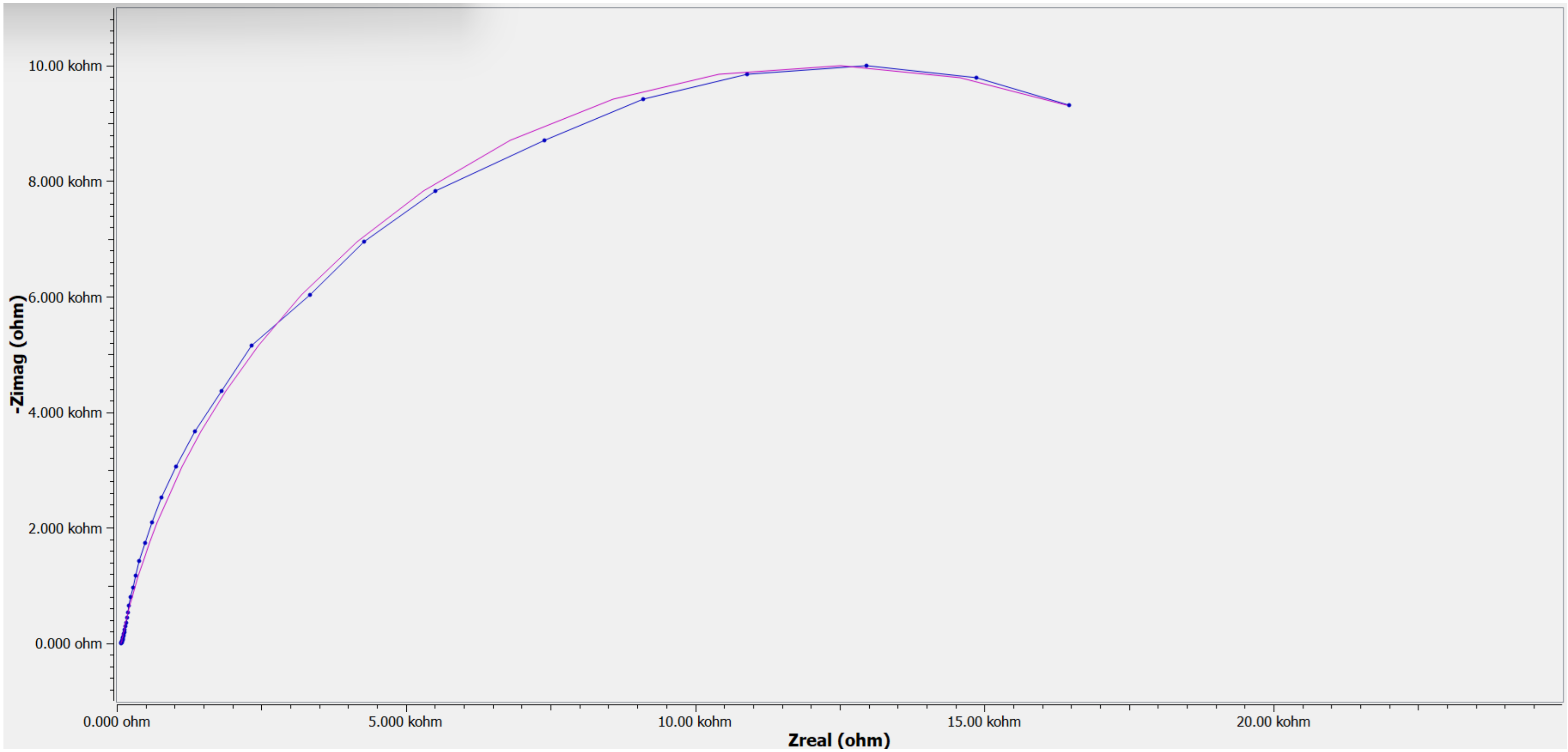

Figure S26. EIS of gold-MUA E6.

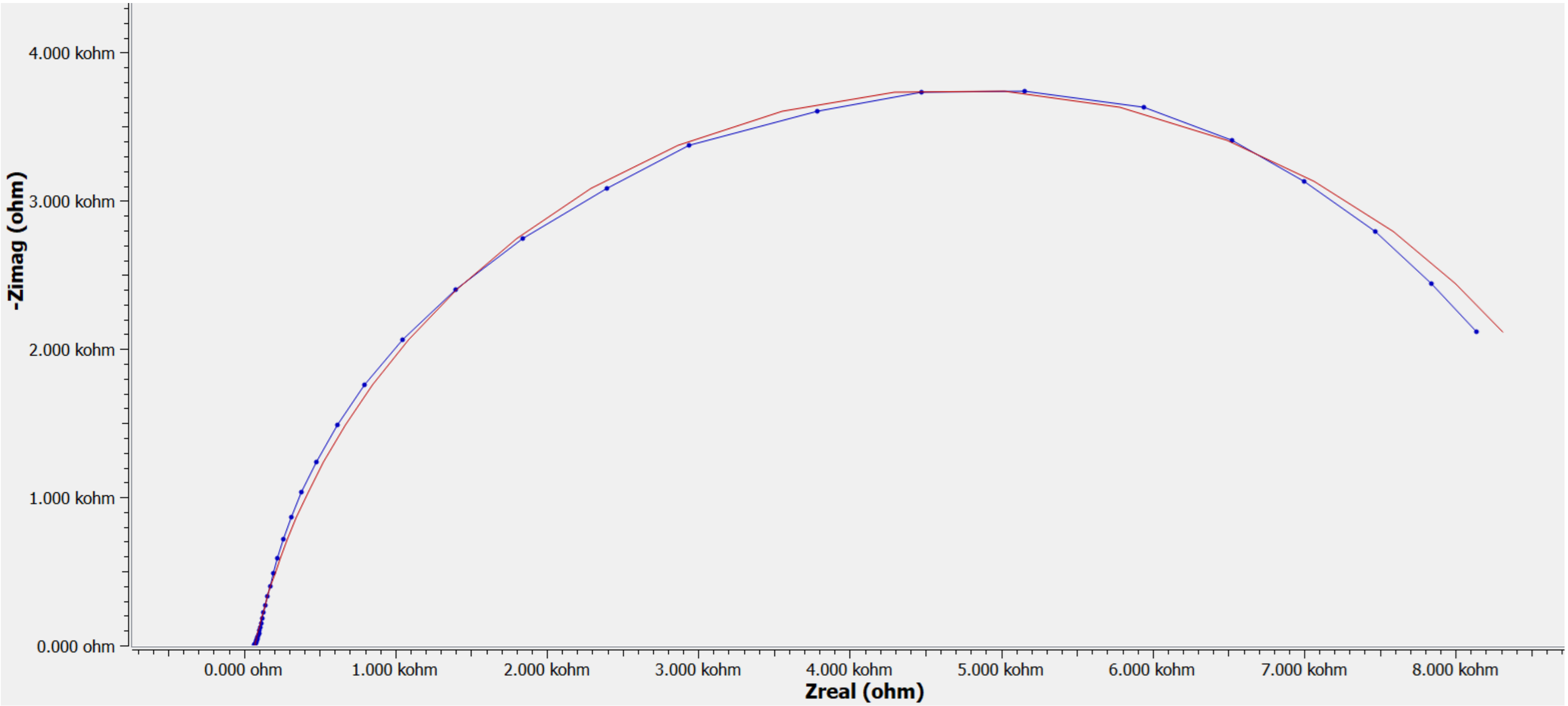

Figure S27. EIS of gold-MUA-CD144AB E6.

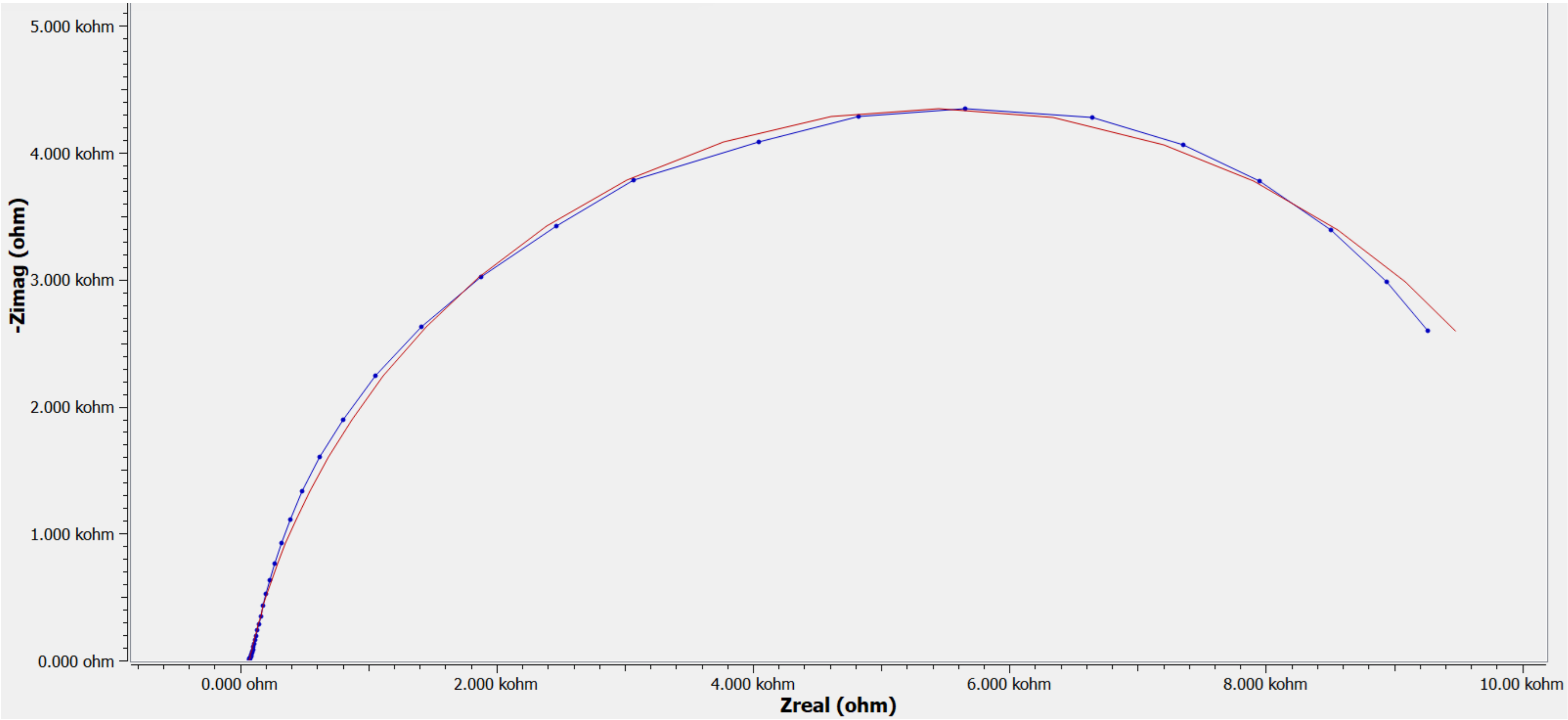

Figure S28. EIS of gold-MUA-CD144AB-EV(std. 2) E6.

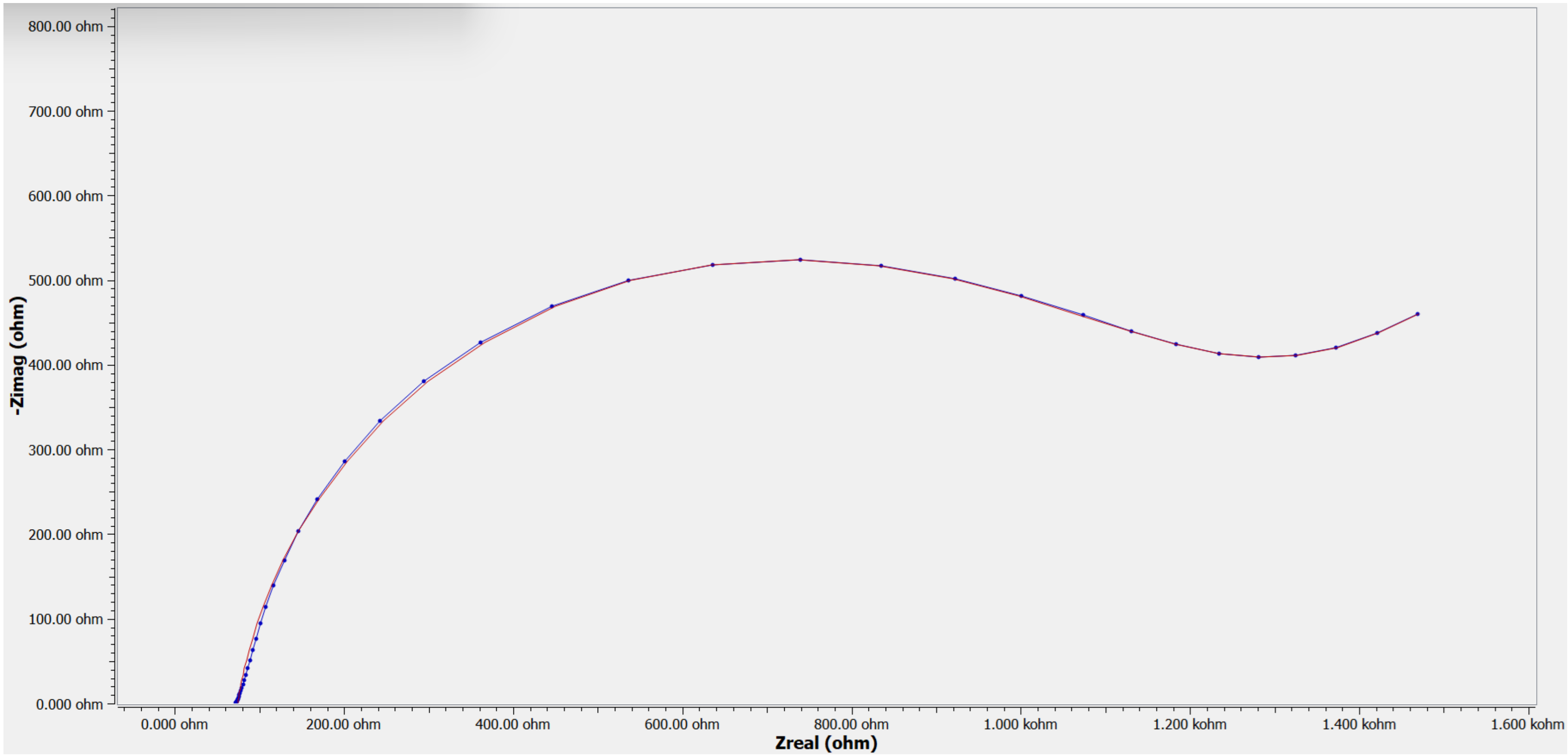

Figure S29. EIS of gold bare E7.

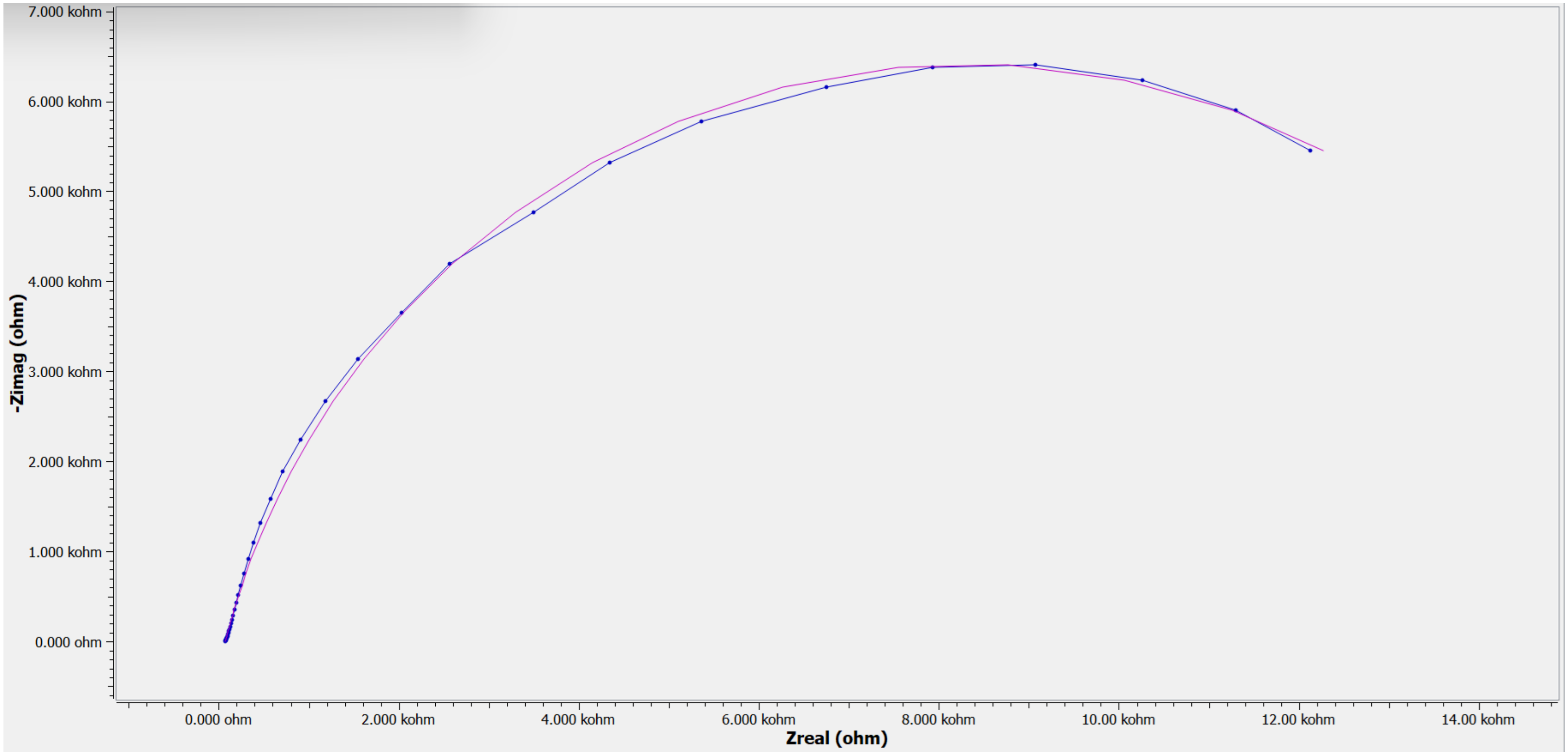

Figure S30. EIS of gold-MUA E7.

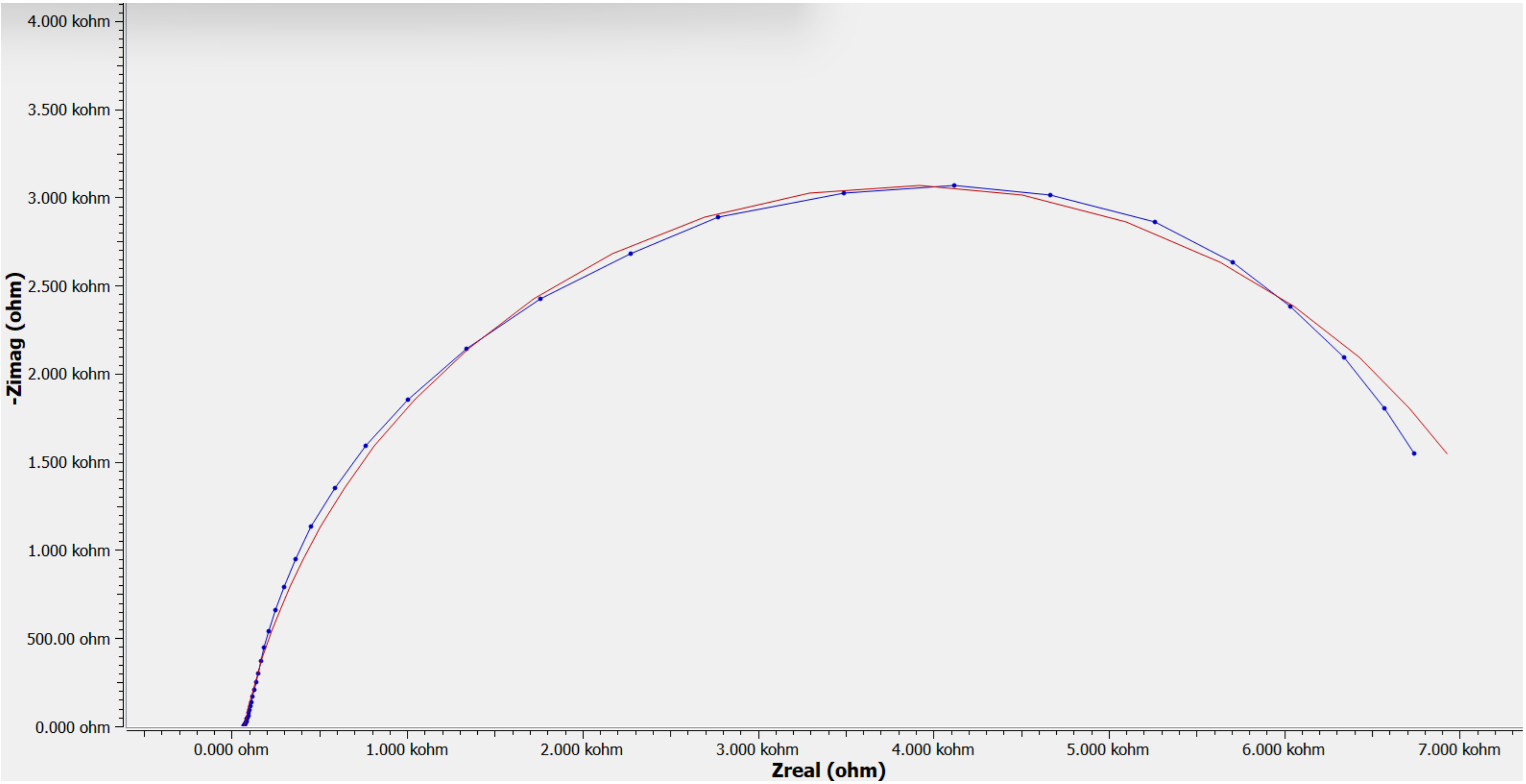

Figure S31. EIS of gold-MUA-CD144AB E7.

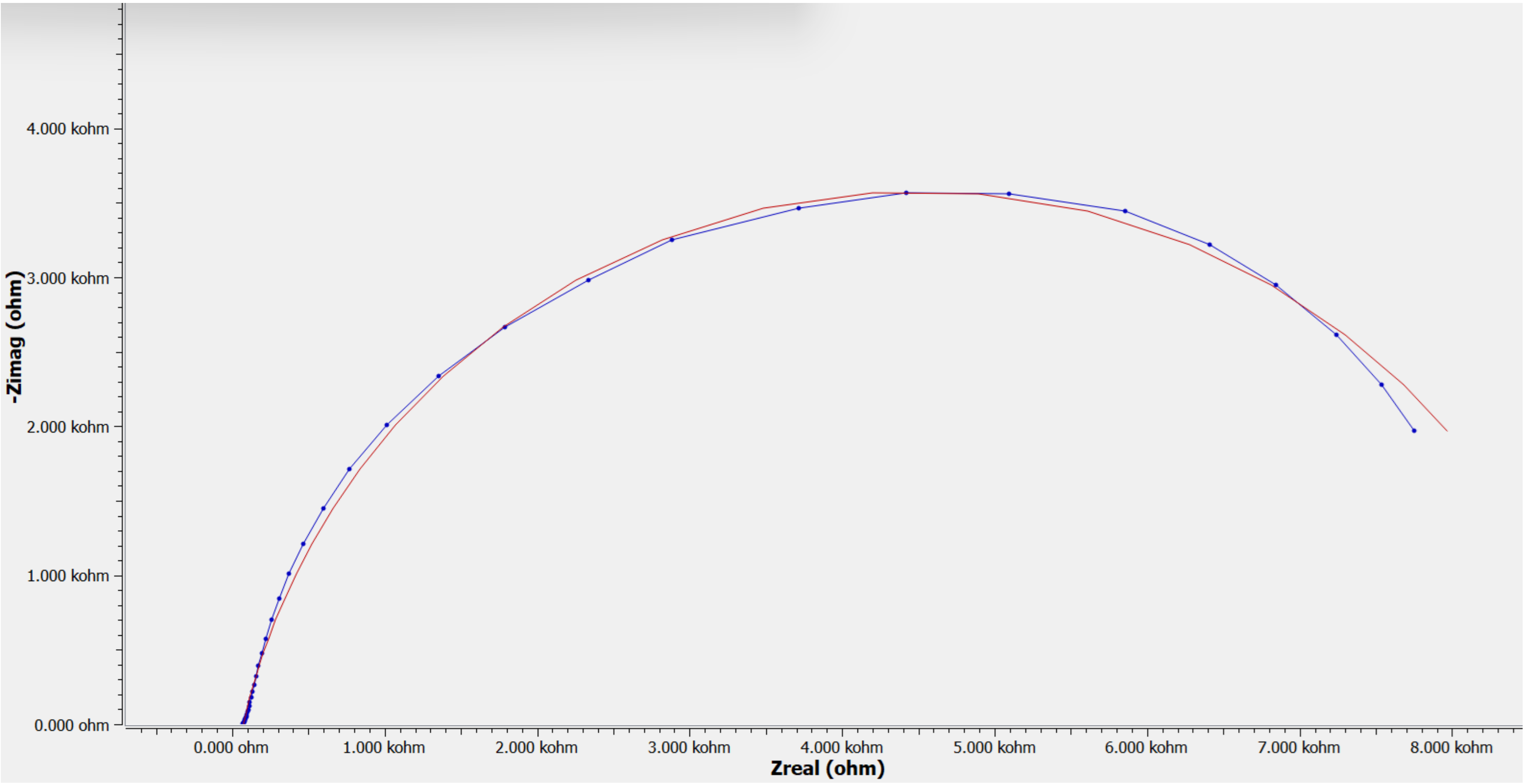

Figure S32. EIS of gold-MUA-CD144AB-EV(std. 3) E7.

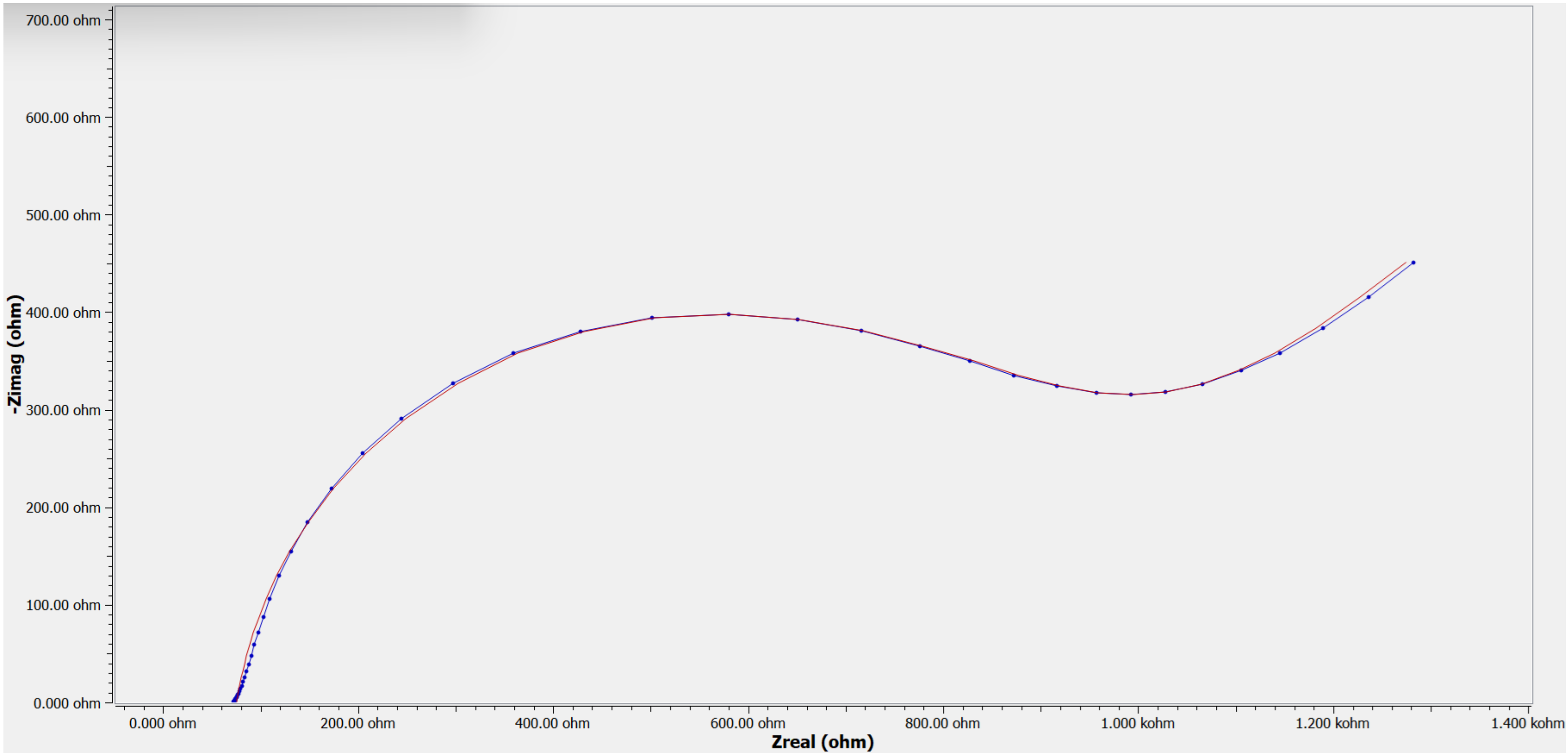

Figure S33. EIS of gold bare E8.

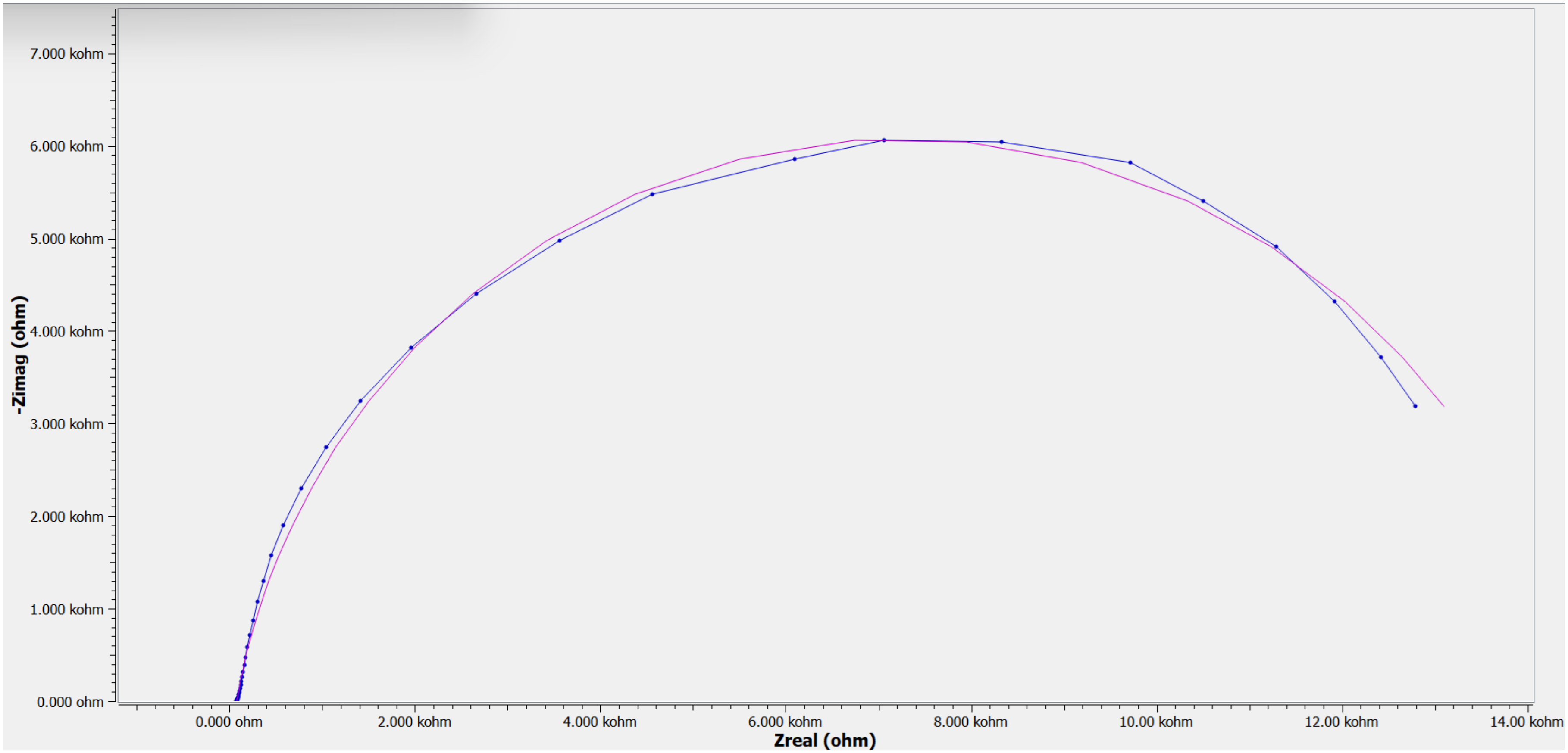

Figure S34. EIS of gold-MUA E8.

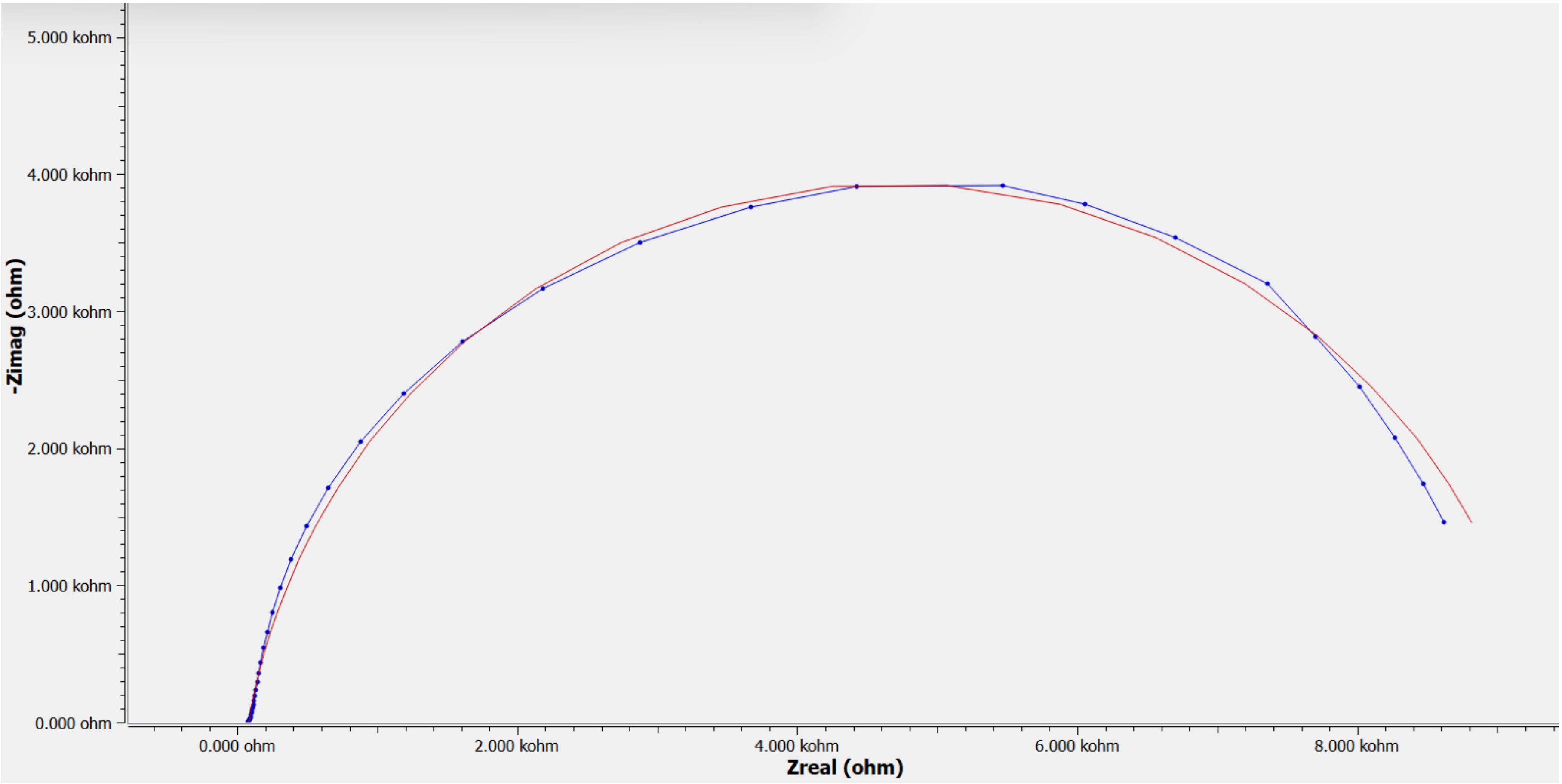

Figure S35. EIS of gold-MUA-CD144AB E8.

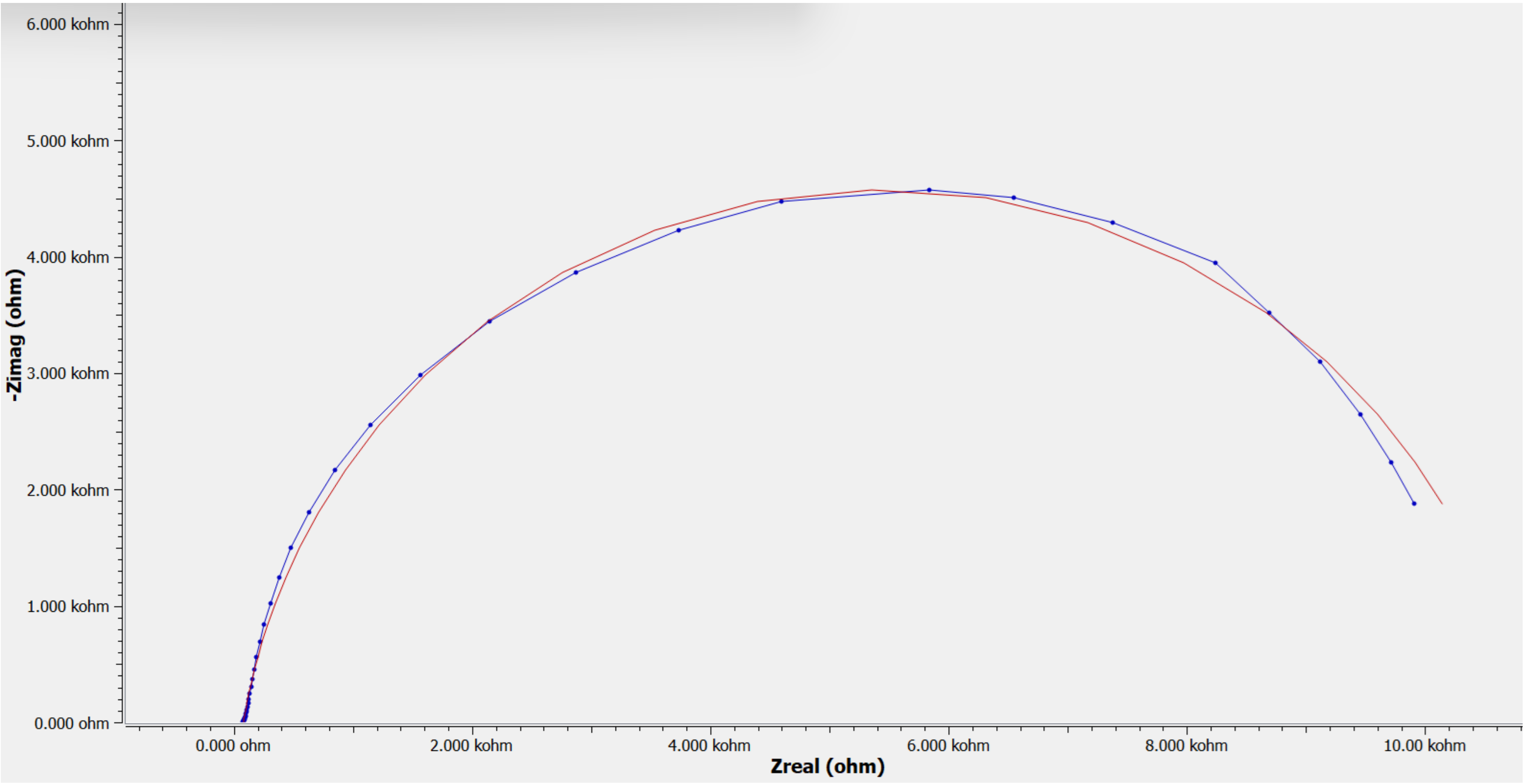

Figure S36. EIS of gold-MUA-CD144AB-EV(std. 3) E8.

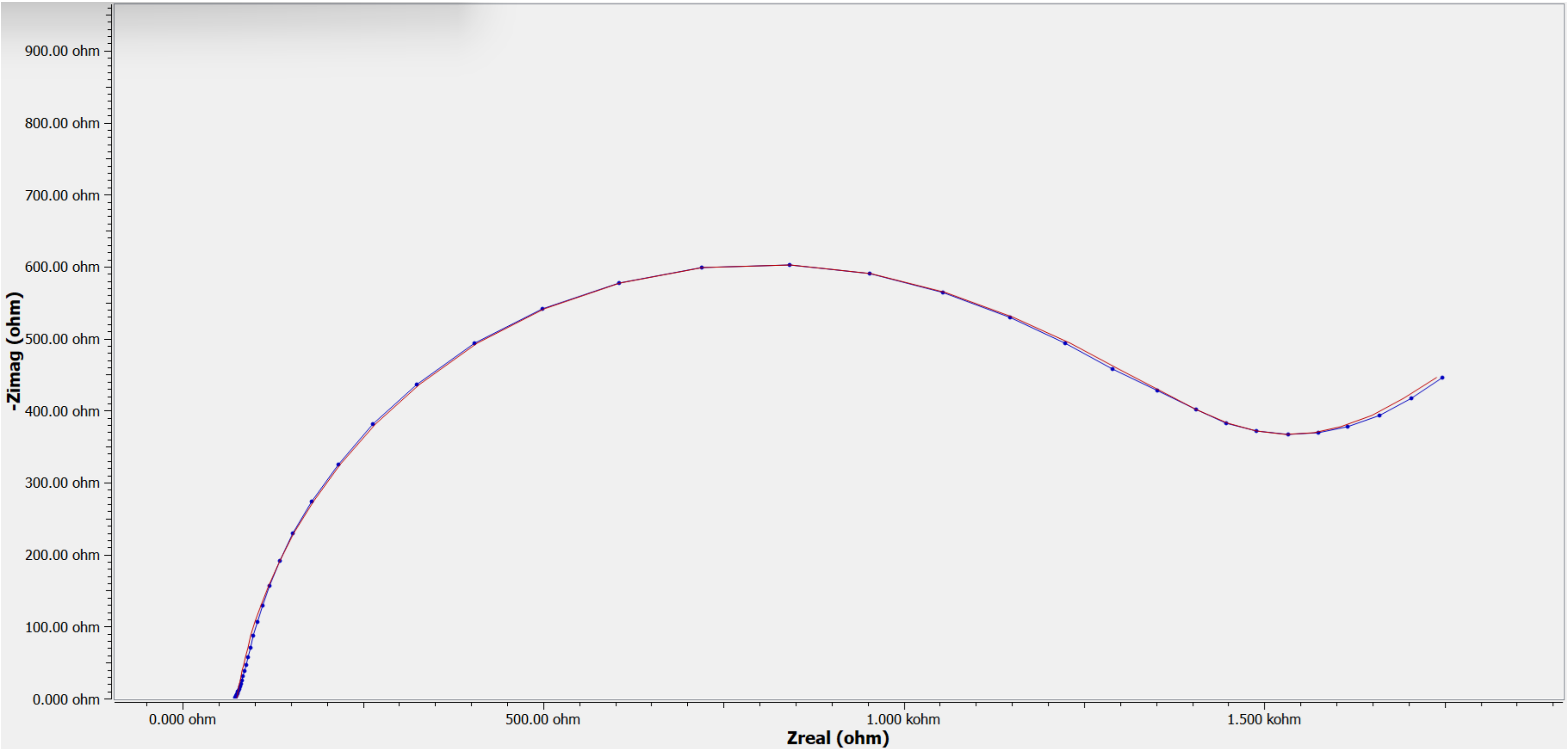

Figure S37. EIS of gold bare E9.

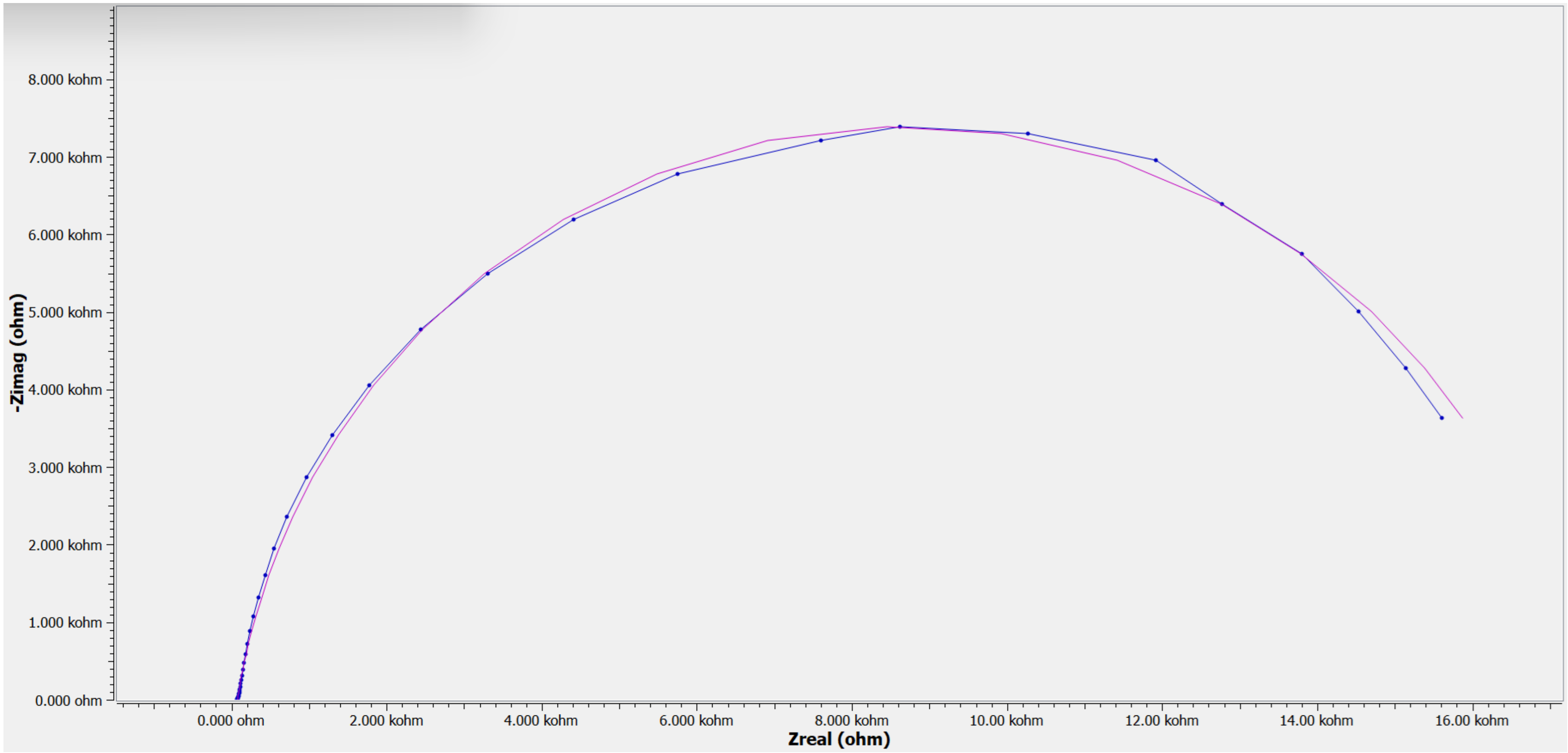

Figure S38. EIS of gold-MUA E9.

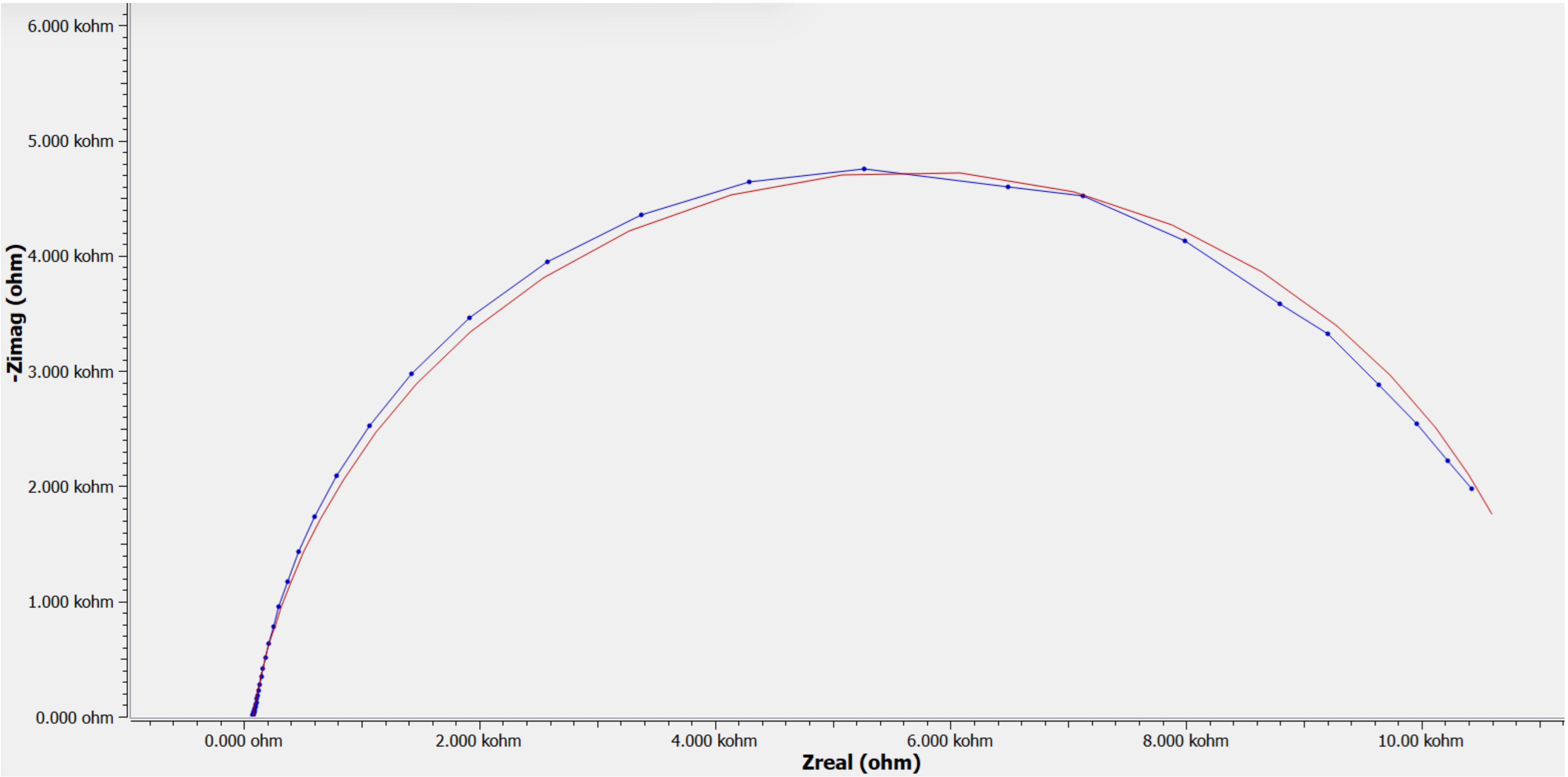

Figure S39. EIS of gold-MUA-CD144AB E9.

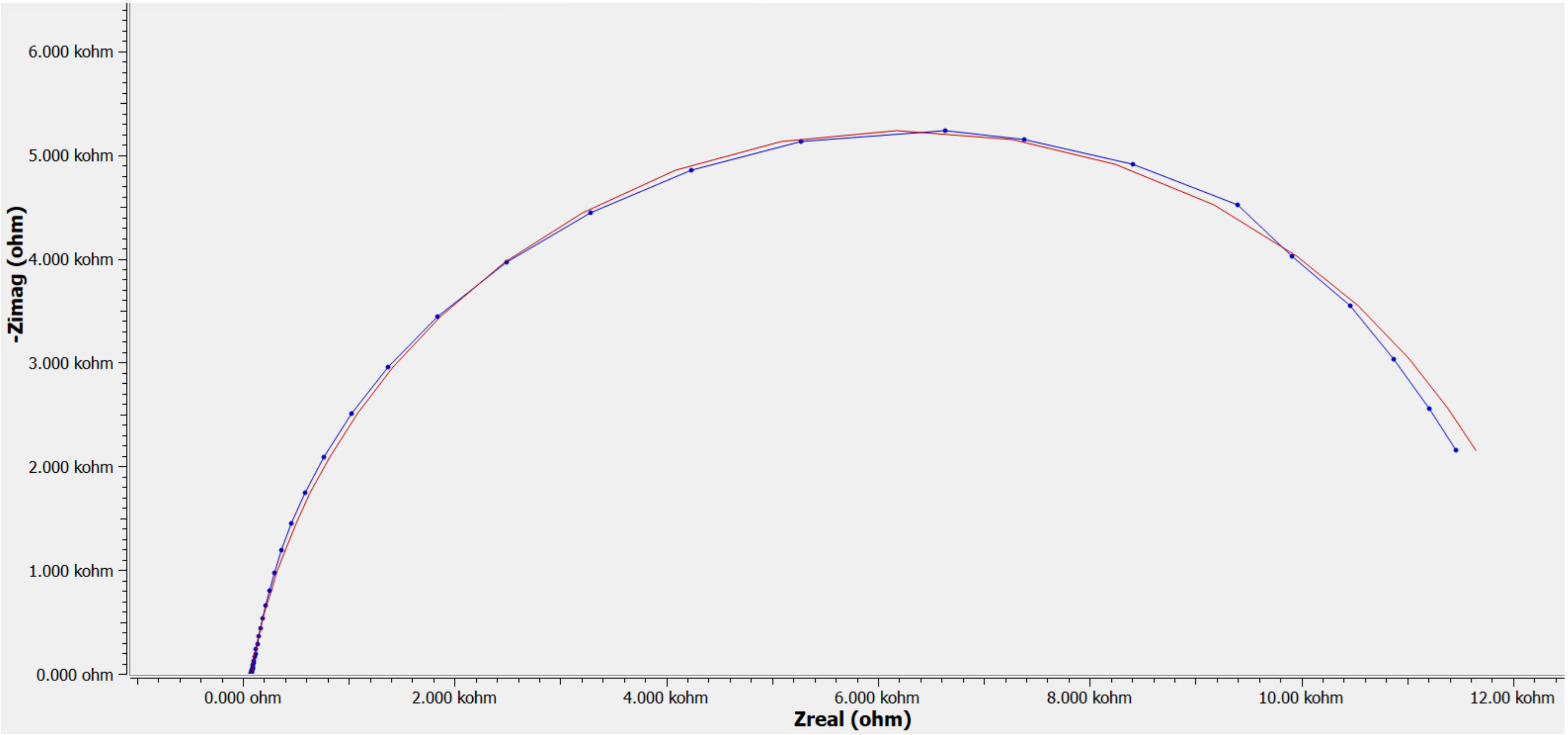

Figure S40. EIS of gold-MUA-CD144AB-EV(std. 3) E9.

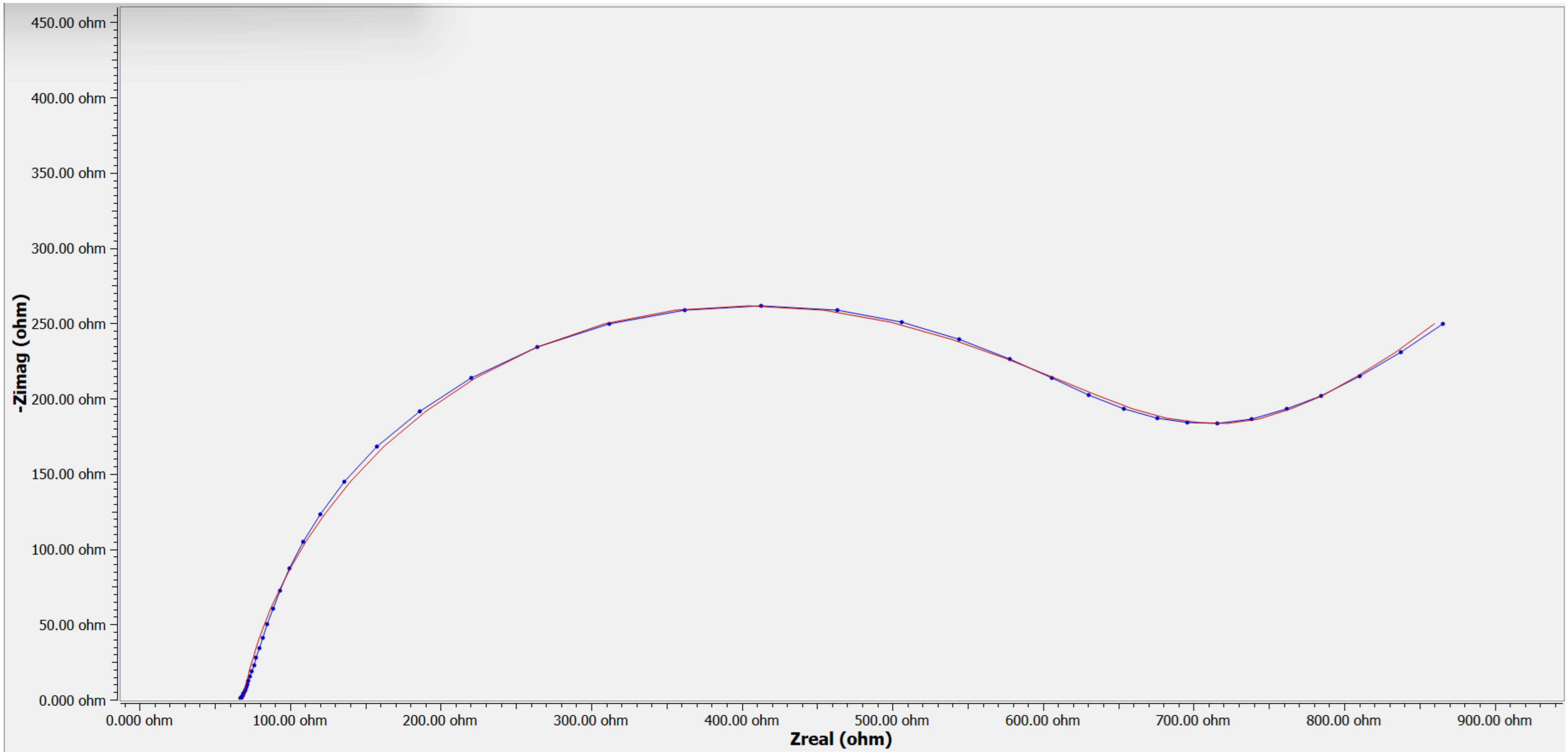

Figure S41. EIS of gold bare E10.

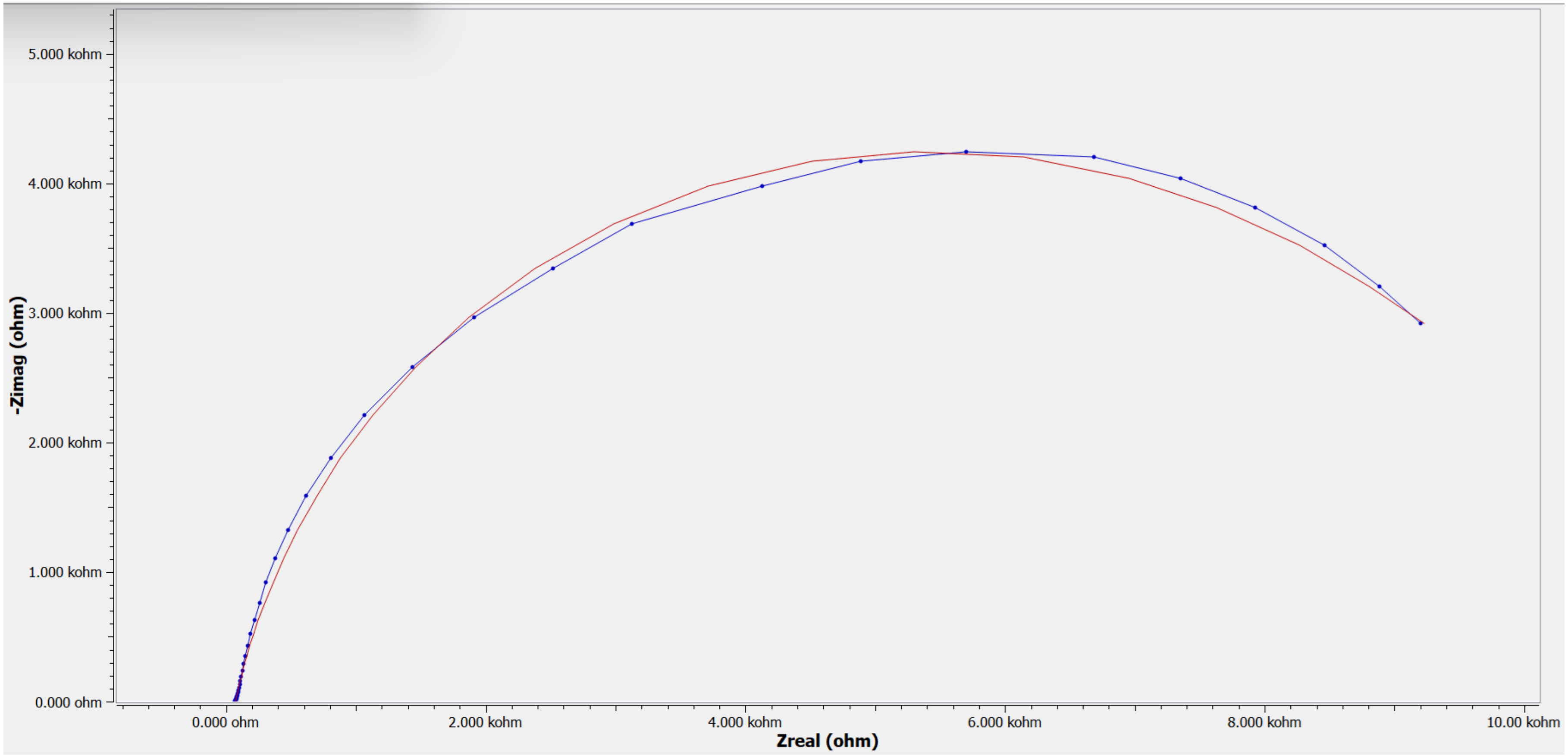

Figure S42. EIS of gold-MUA E10.

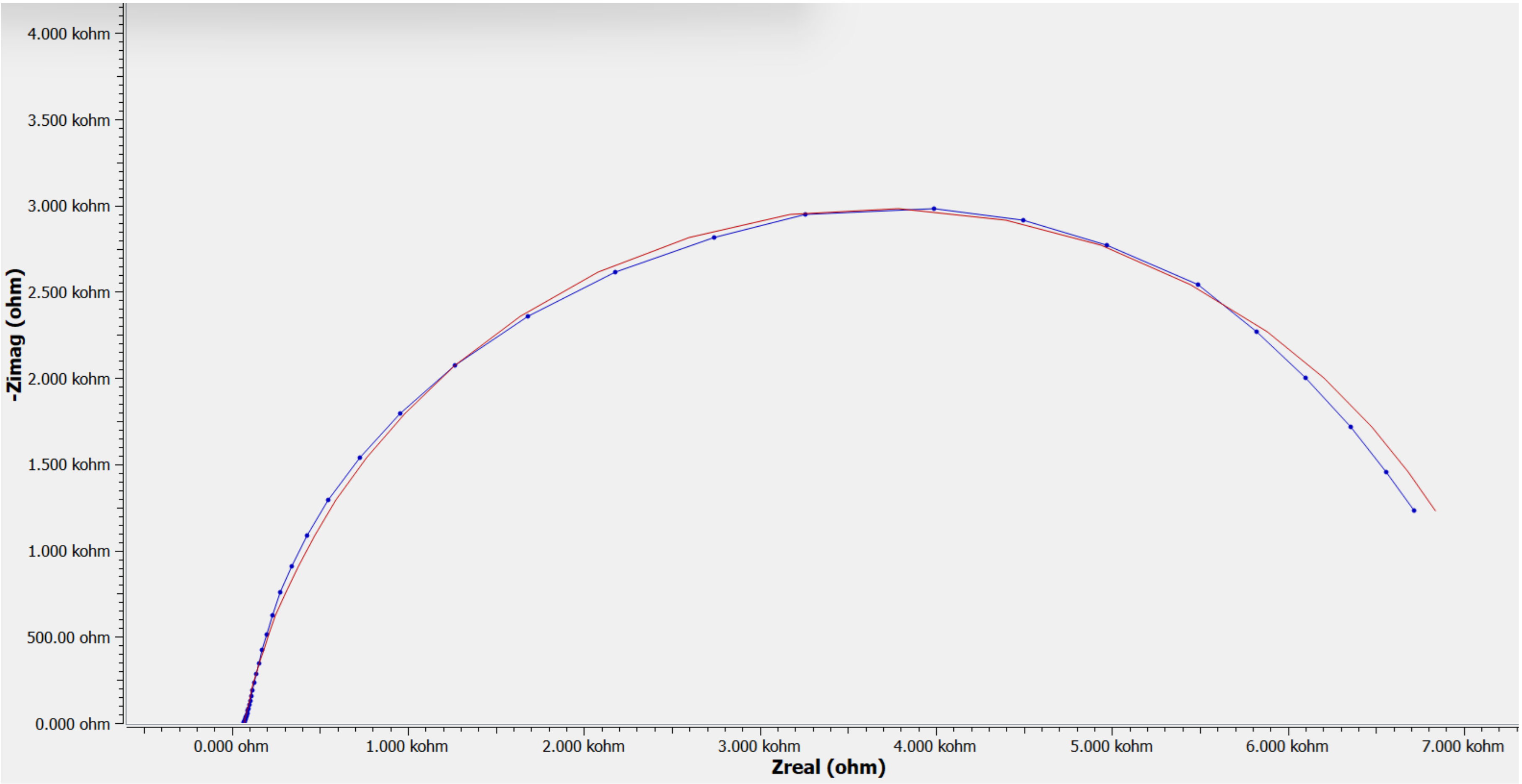

Figure S43. EIS of gold-MUA-CD144AB E10.

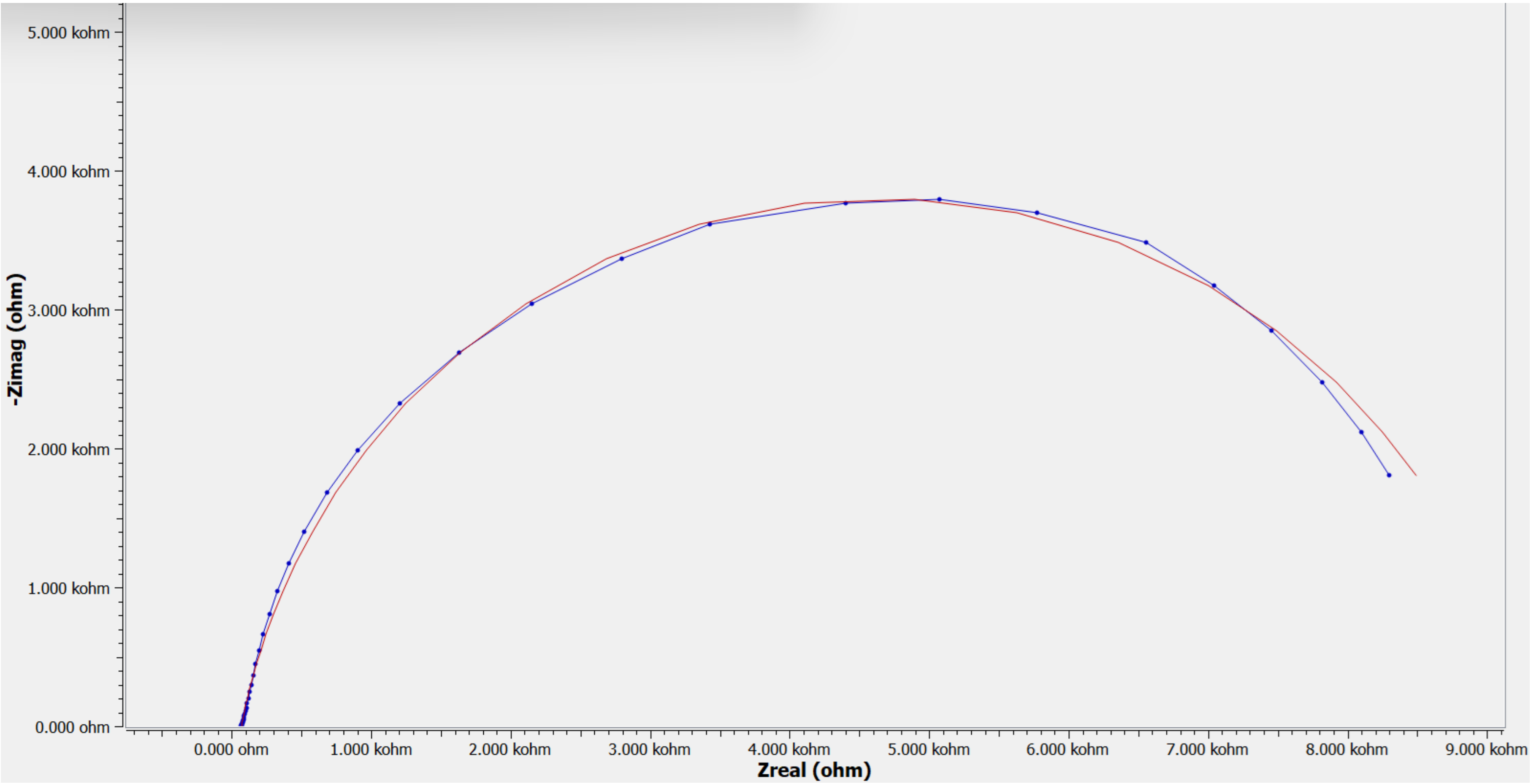

Figure S44. EIS of gold-MUA-CD144AB-EV(std. 4) E10.

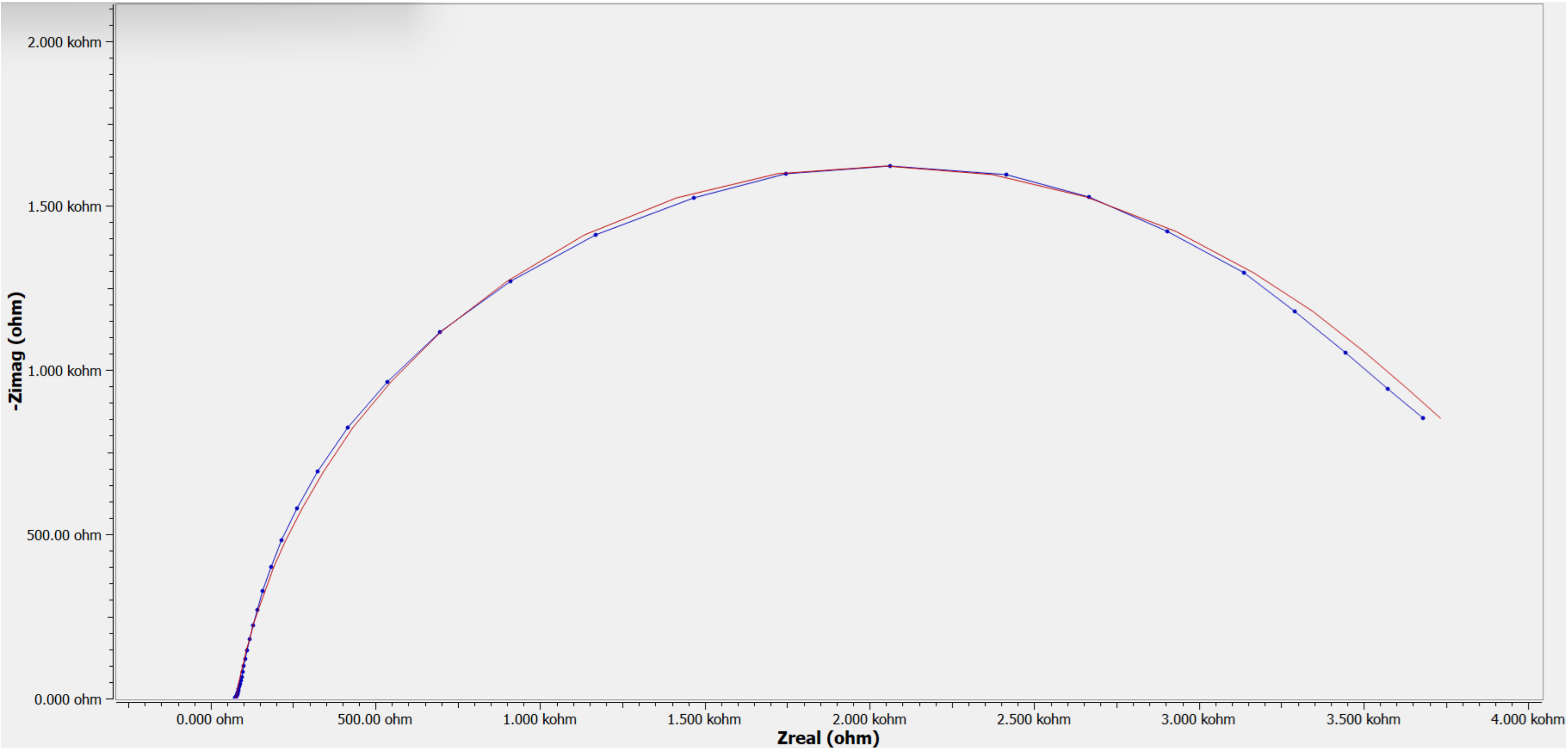

Figure S45. EIS of gold bare E11.

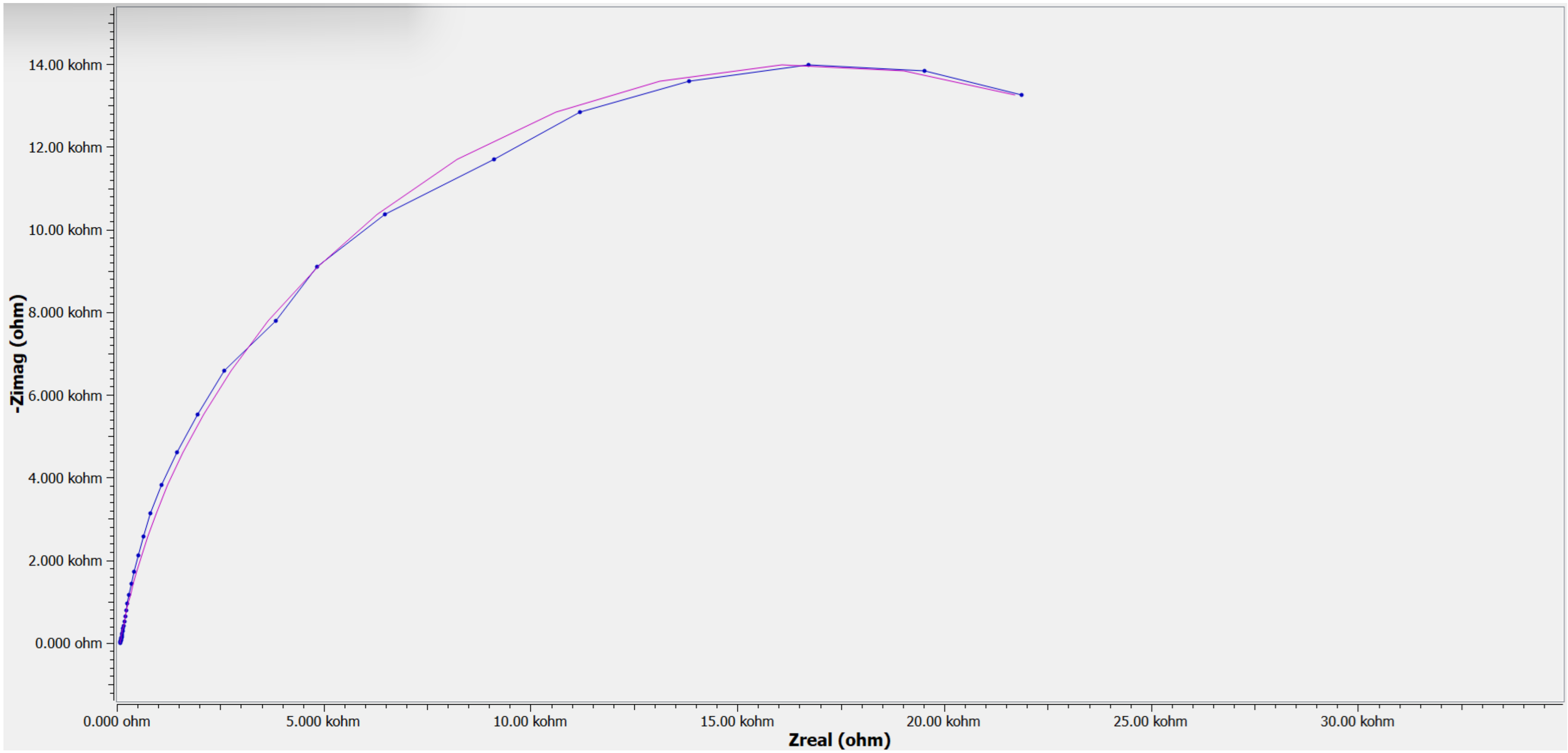

Figure S46. EIS of gold-MUA bare E11.

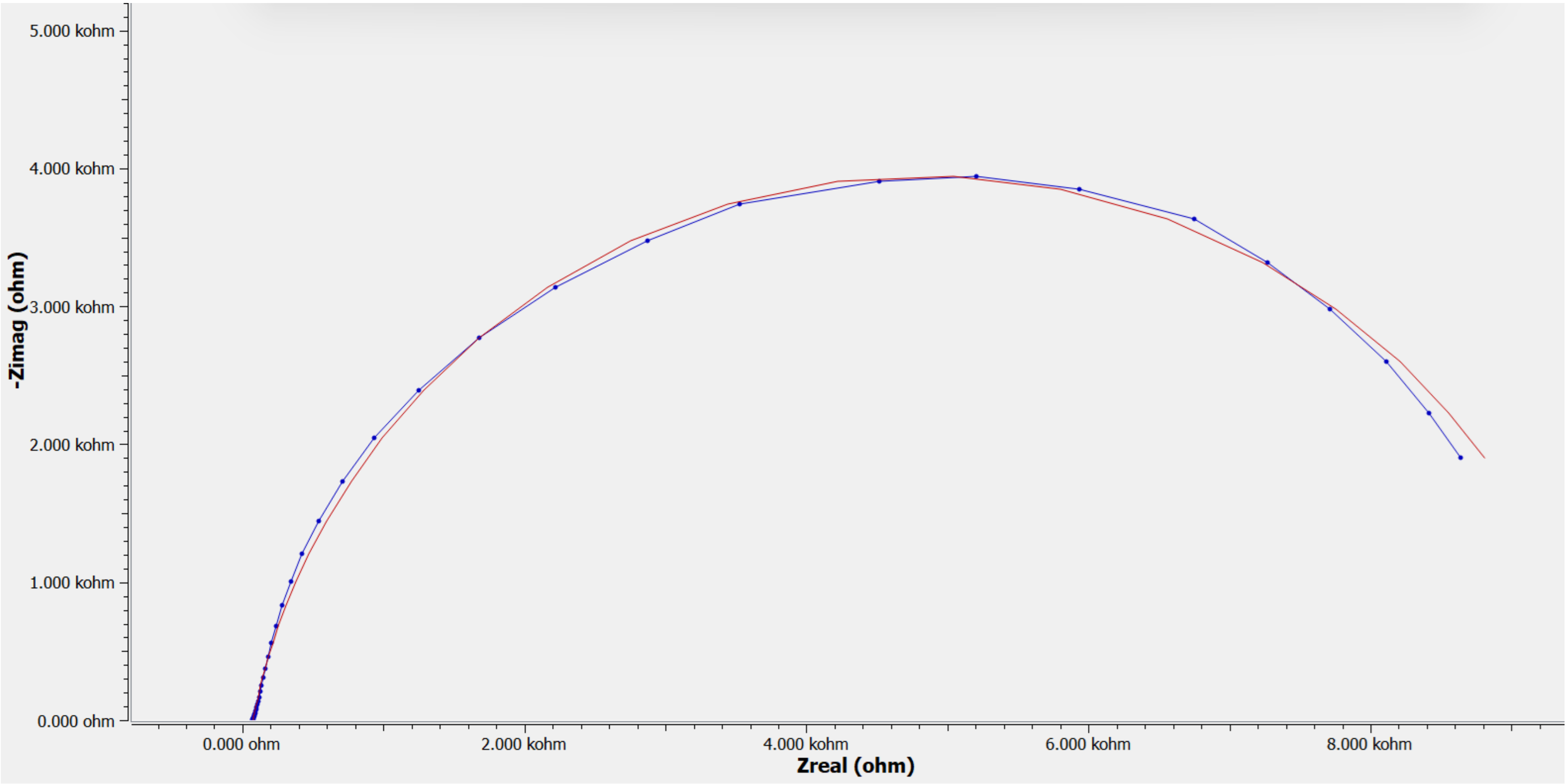

Figure S47. EIS of gold-MUA-CD144AB E11.

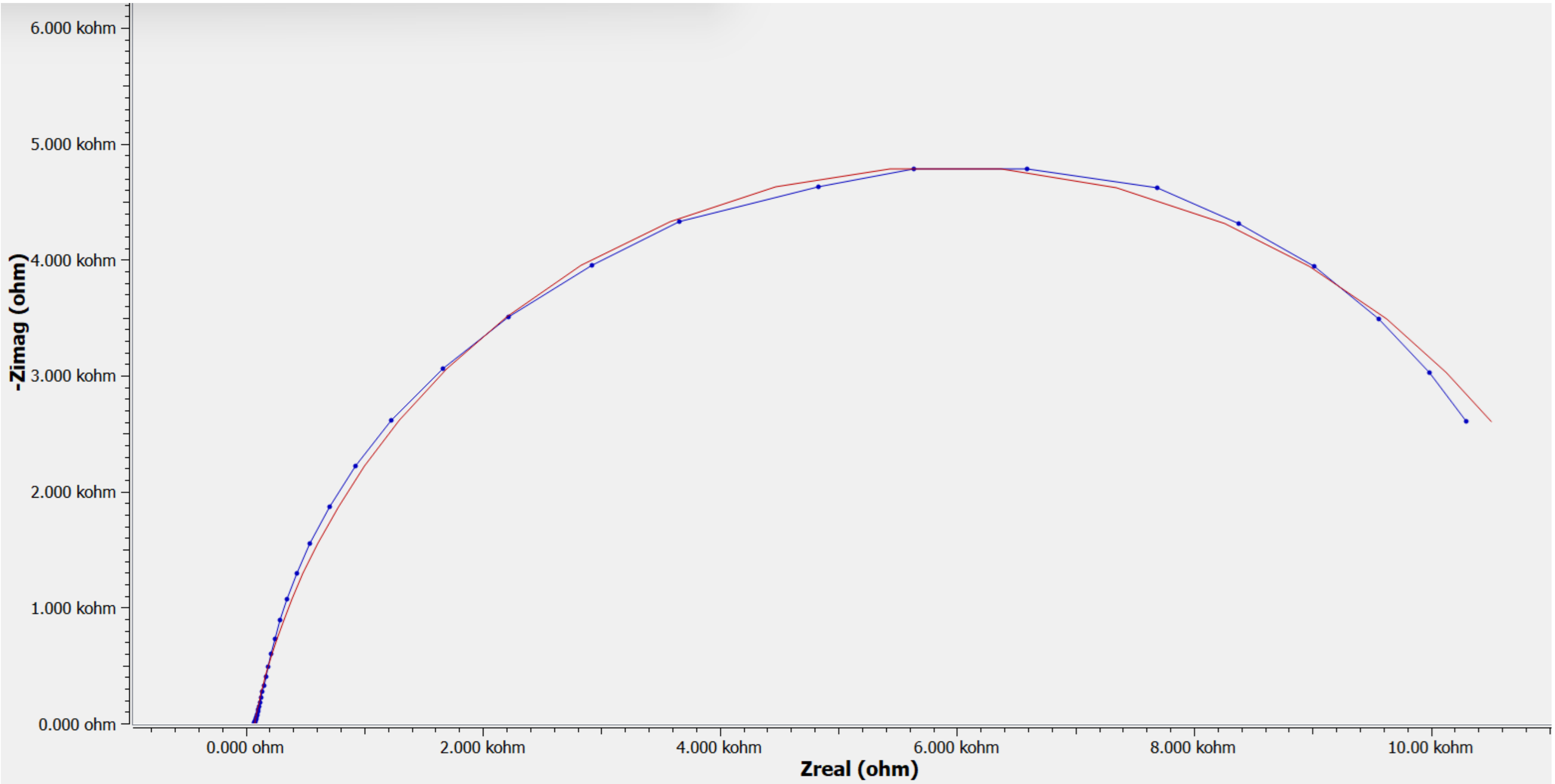

Figure S48. EIS of gold-MUA-CD144AB-EV(std. 4) E11.

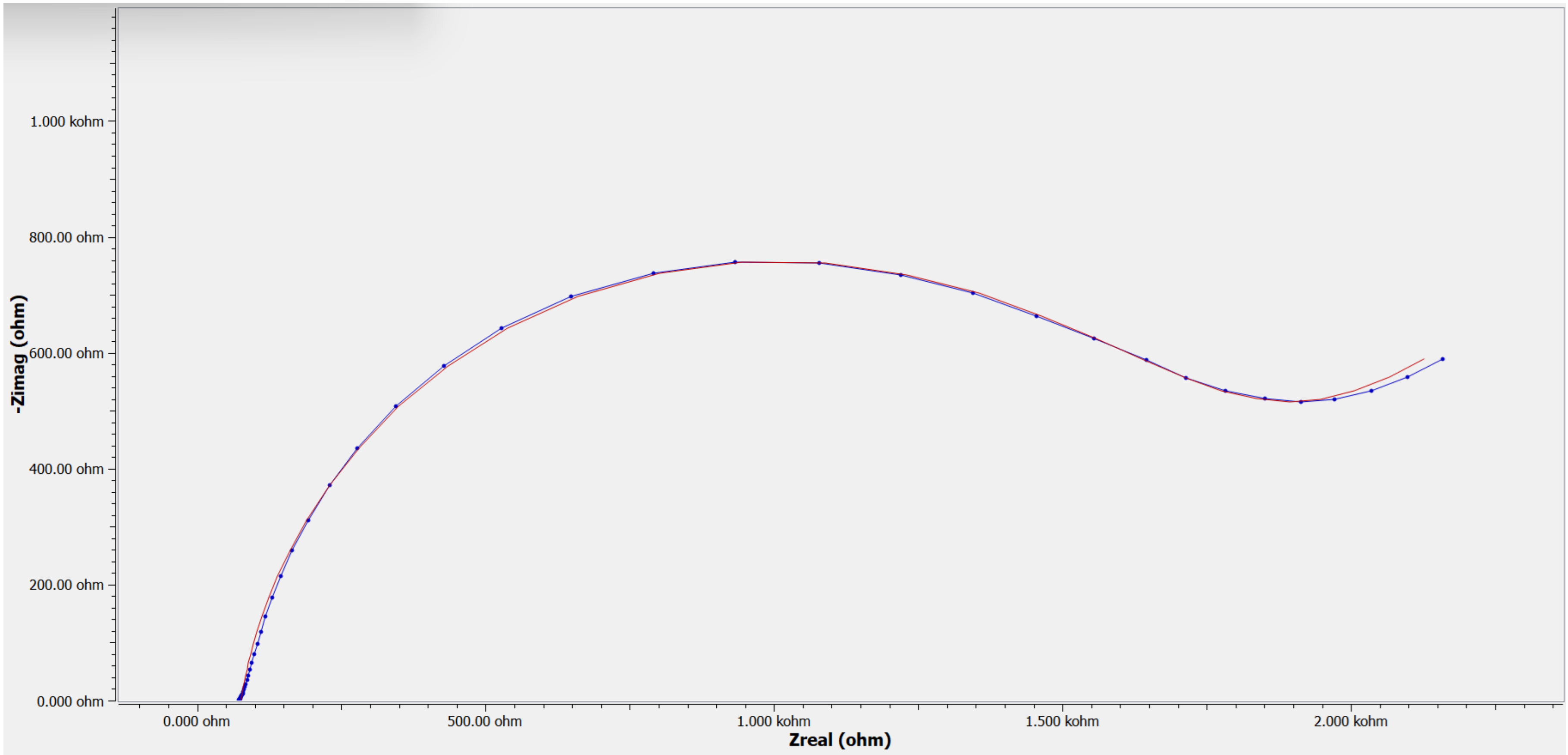

Figure S49. EIS of gold bare E12.

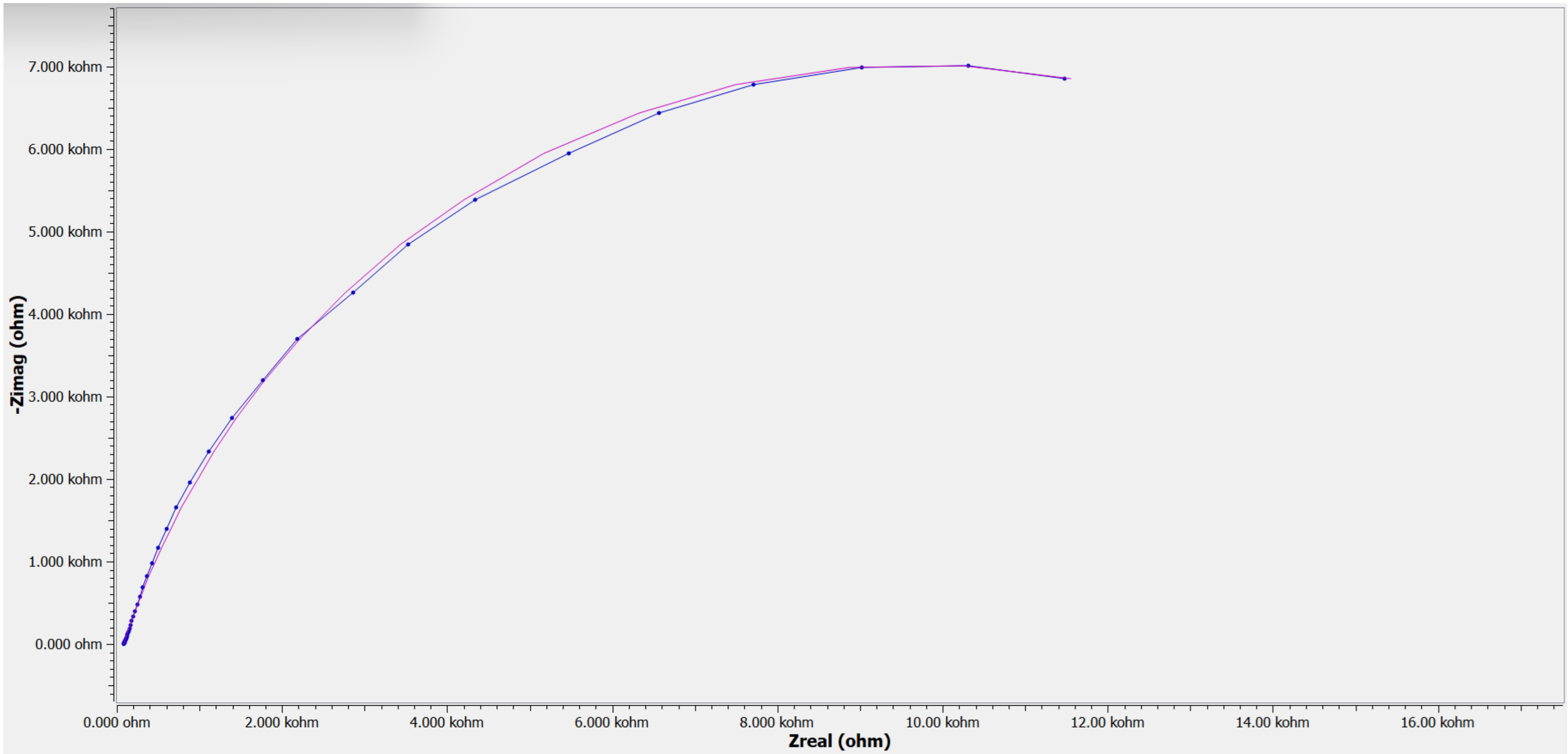

Figure S50. EIS of gold-MUA E12.

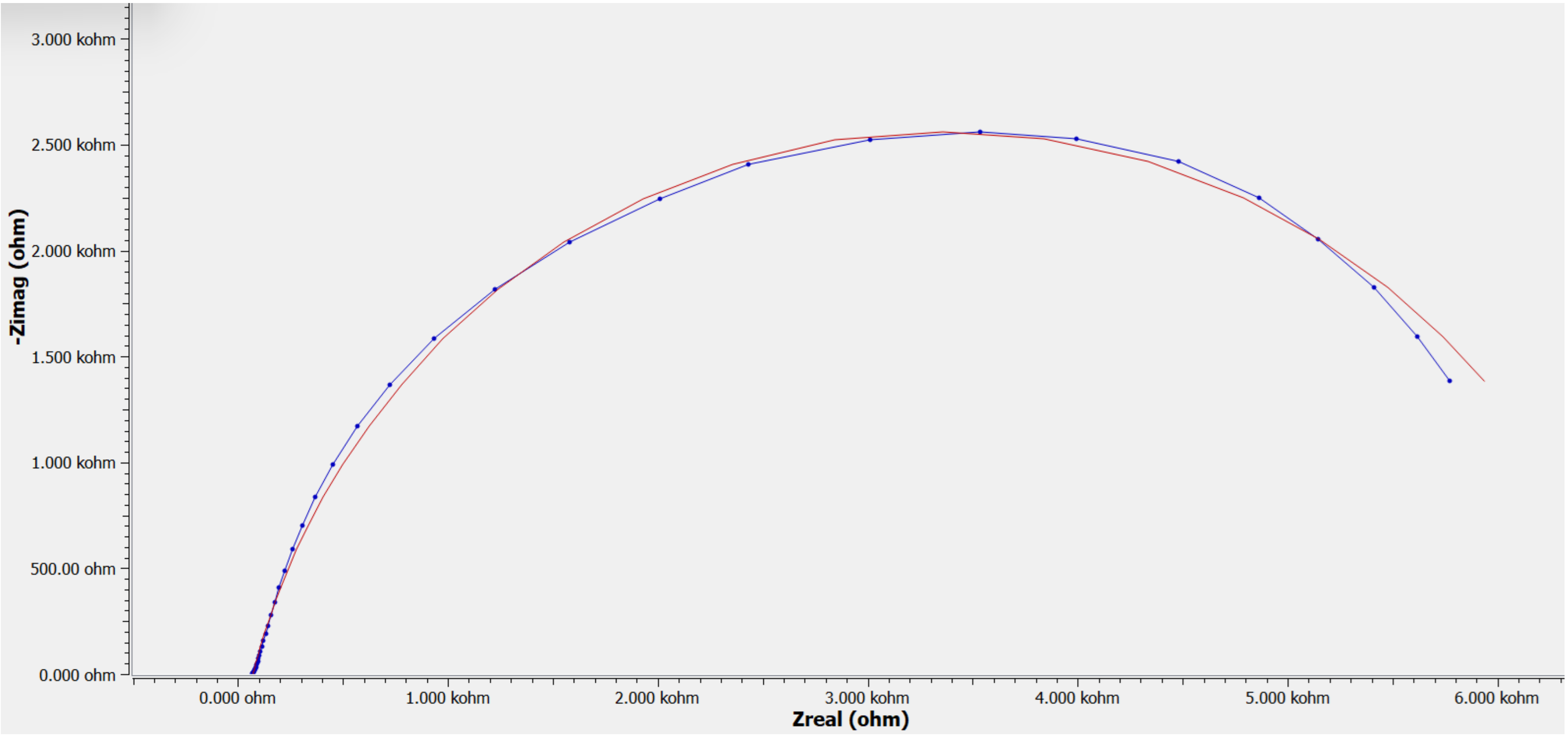

Figure S51. EIS of gold-MUA-CD144AB E12.

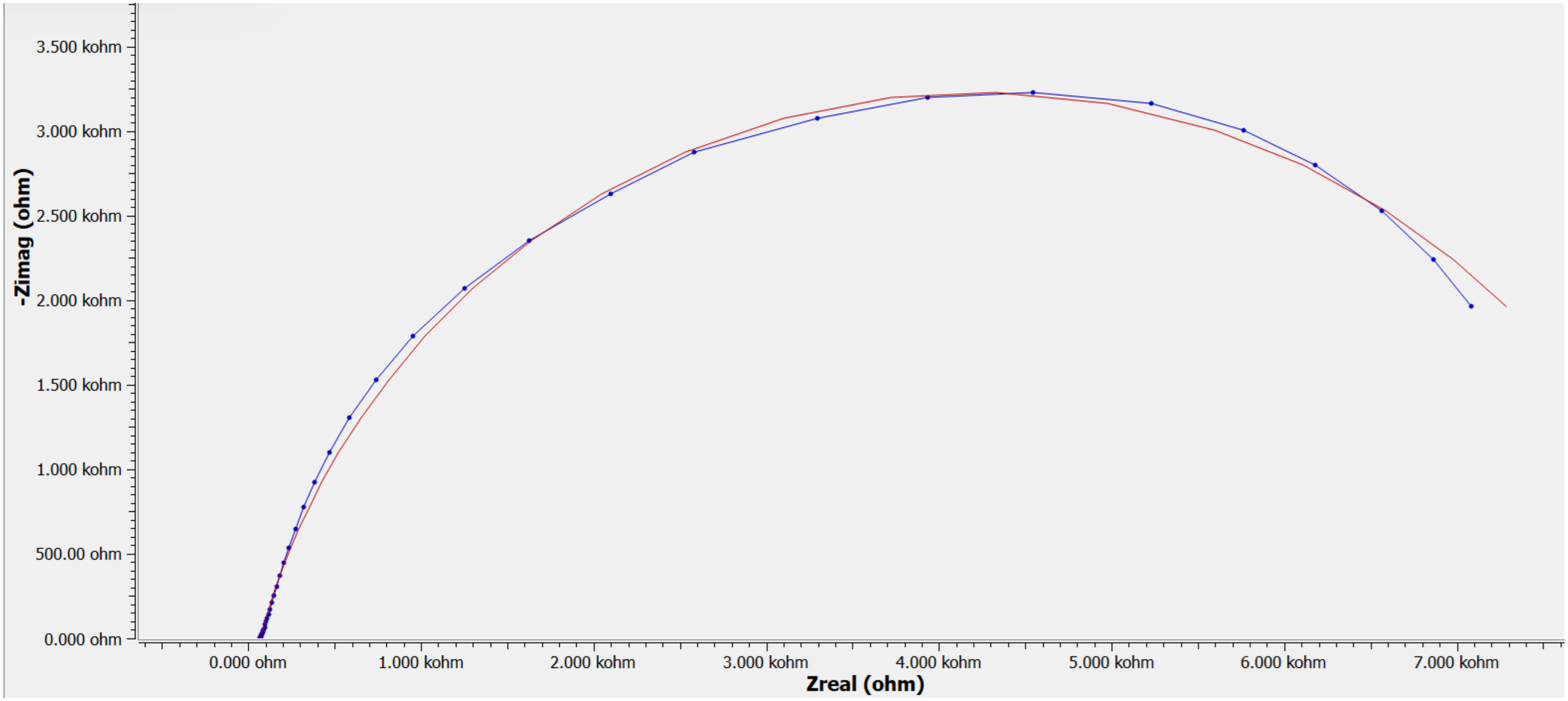

Figure S52. EIS of gold-MUA-CD144AB-EV(std. 4) E12.

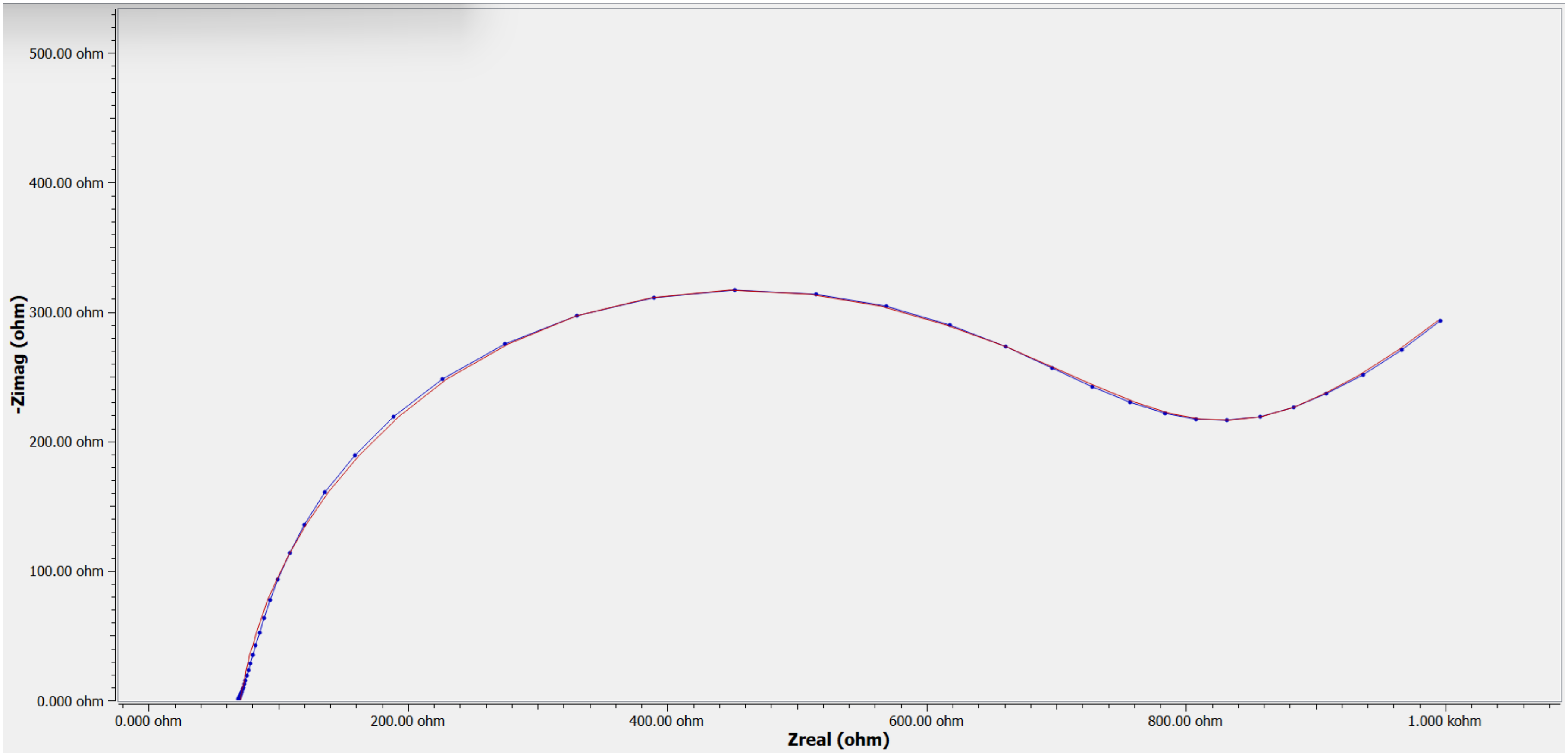

Figure S53. EIS of gold bare E13.

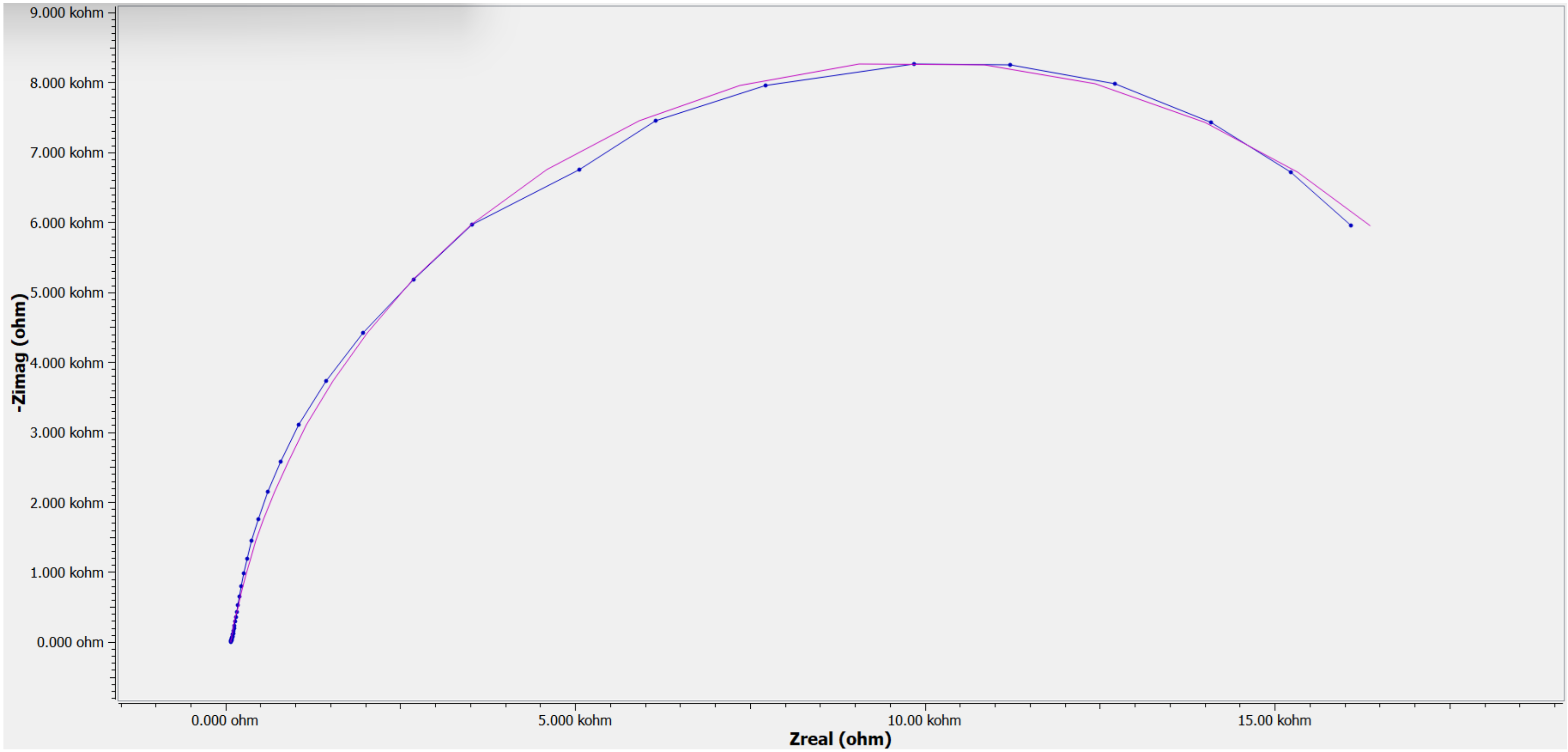

Figure S54. EIS of gold-MUA E13.

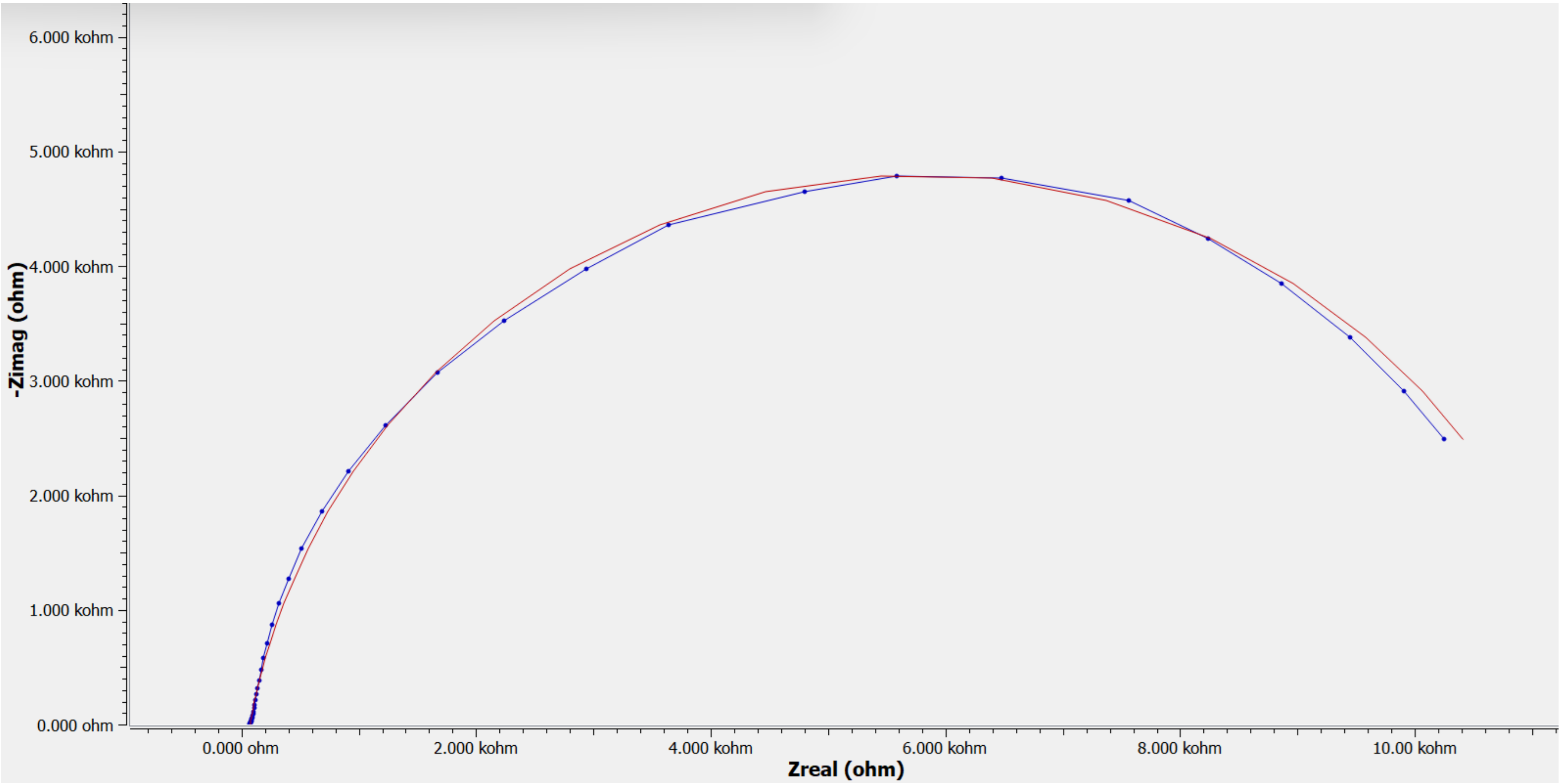

Figure S55. EIS of gold-MUA-CD144AB E13.

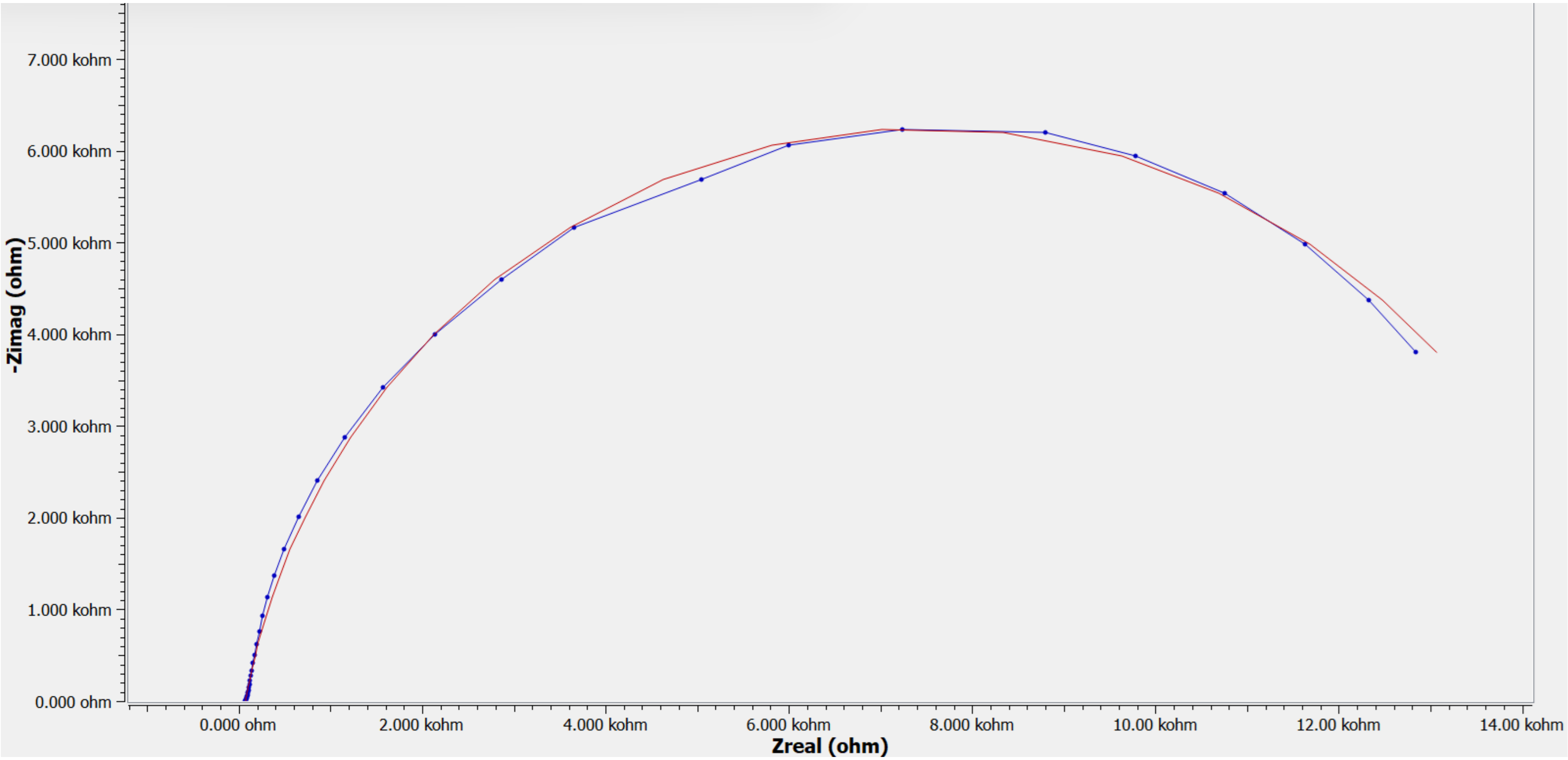

Figure S56. EIS of gold-MUA-CD144AB-EV(std. 5) E13.

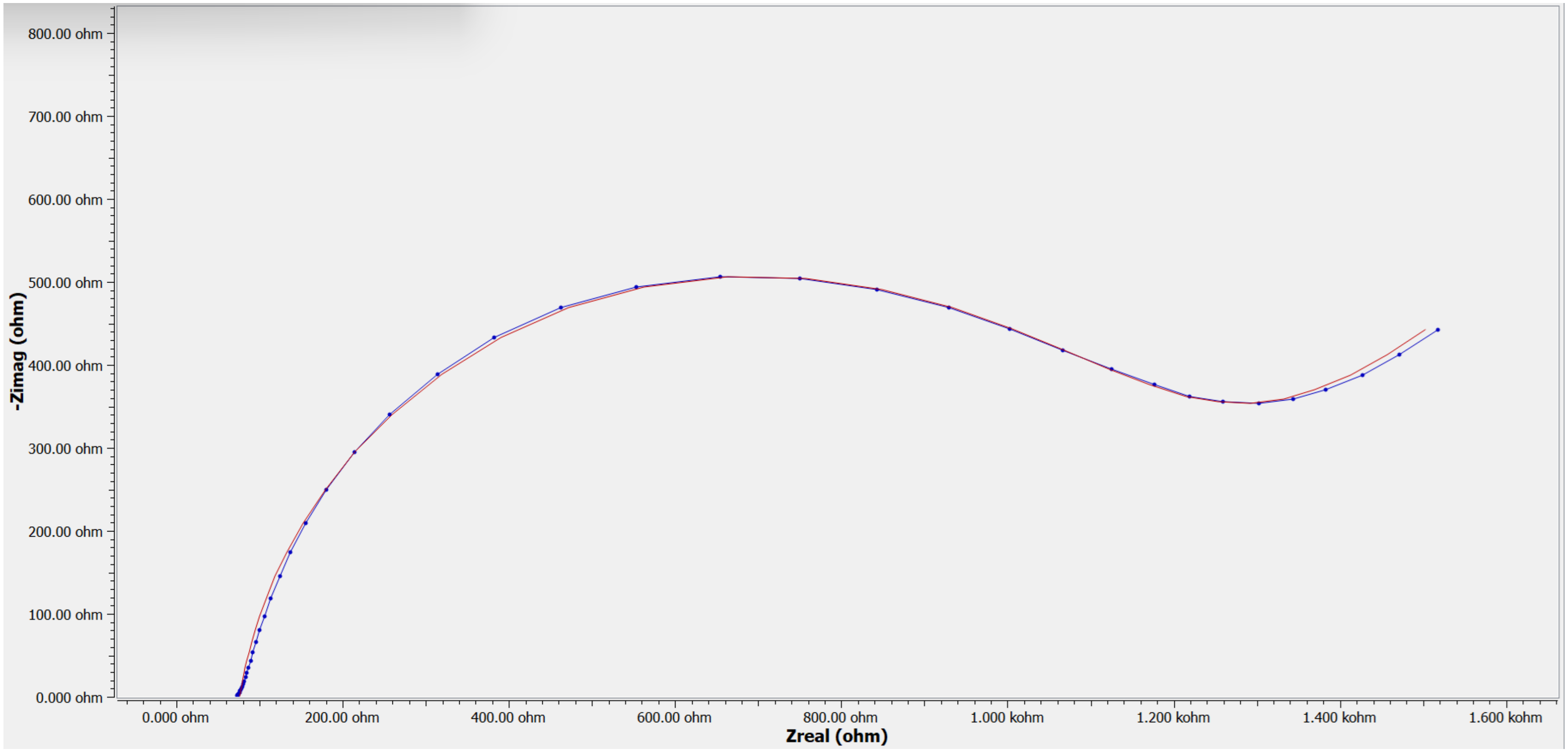

Figure S57. EIS of gold bare E14.

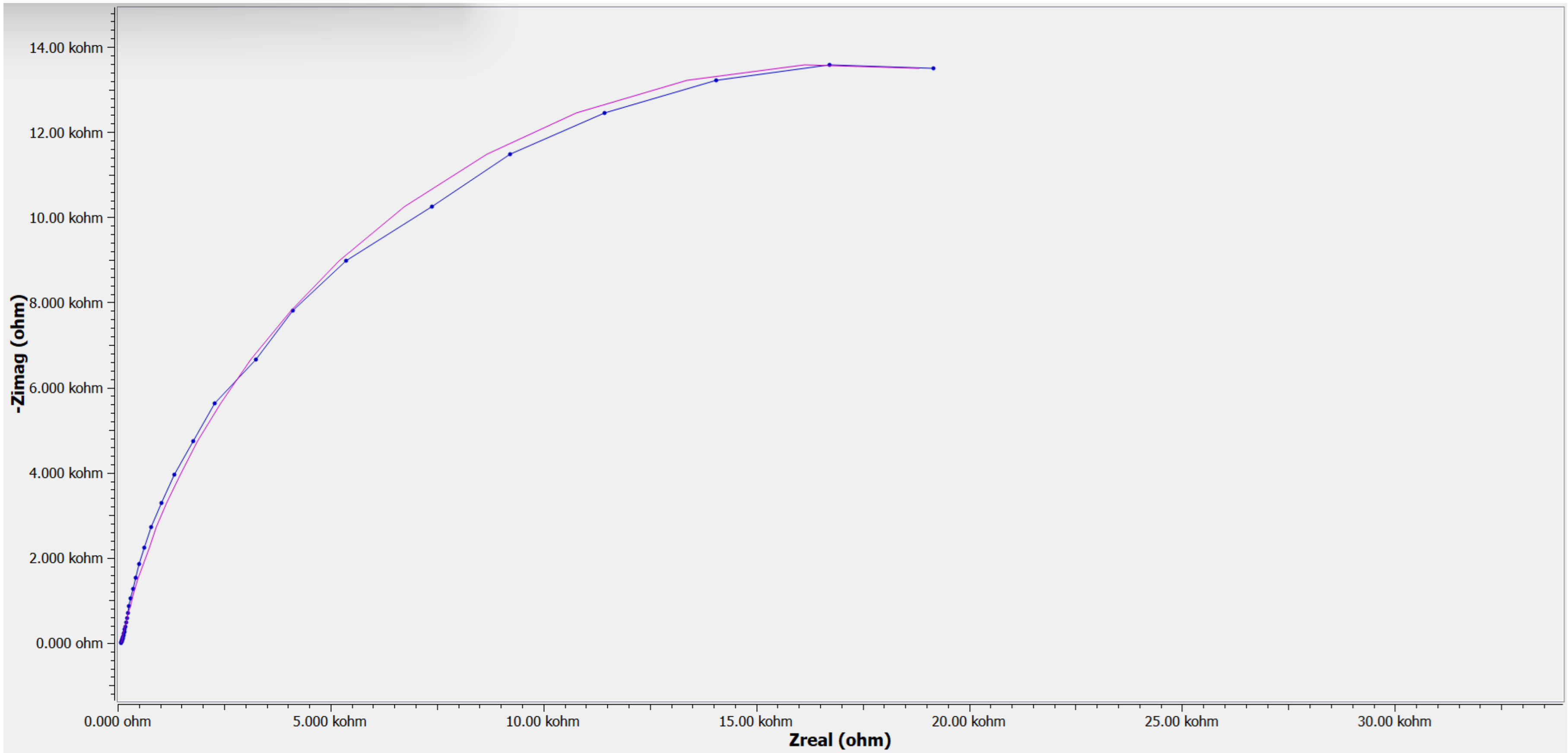

Figure S58. EIS of gold-MUA E14.

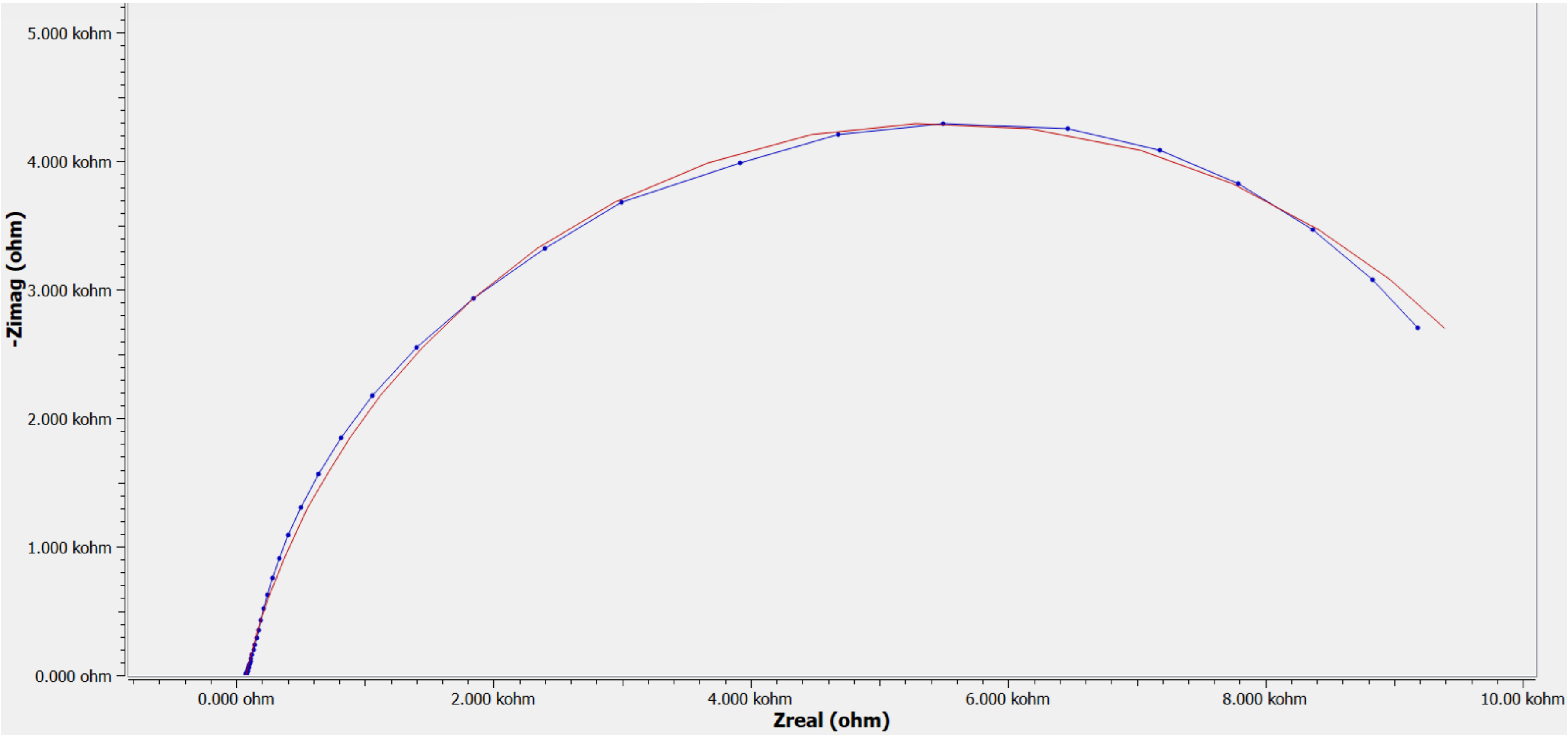

Figure S59. EIS of gold-MUA-CD144AB E14.

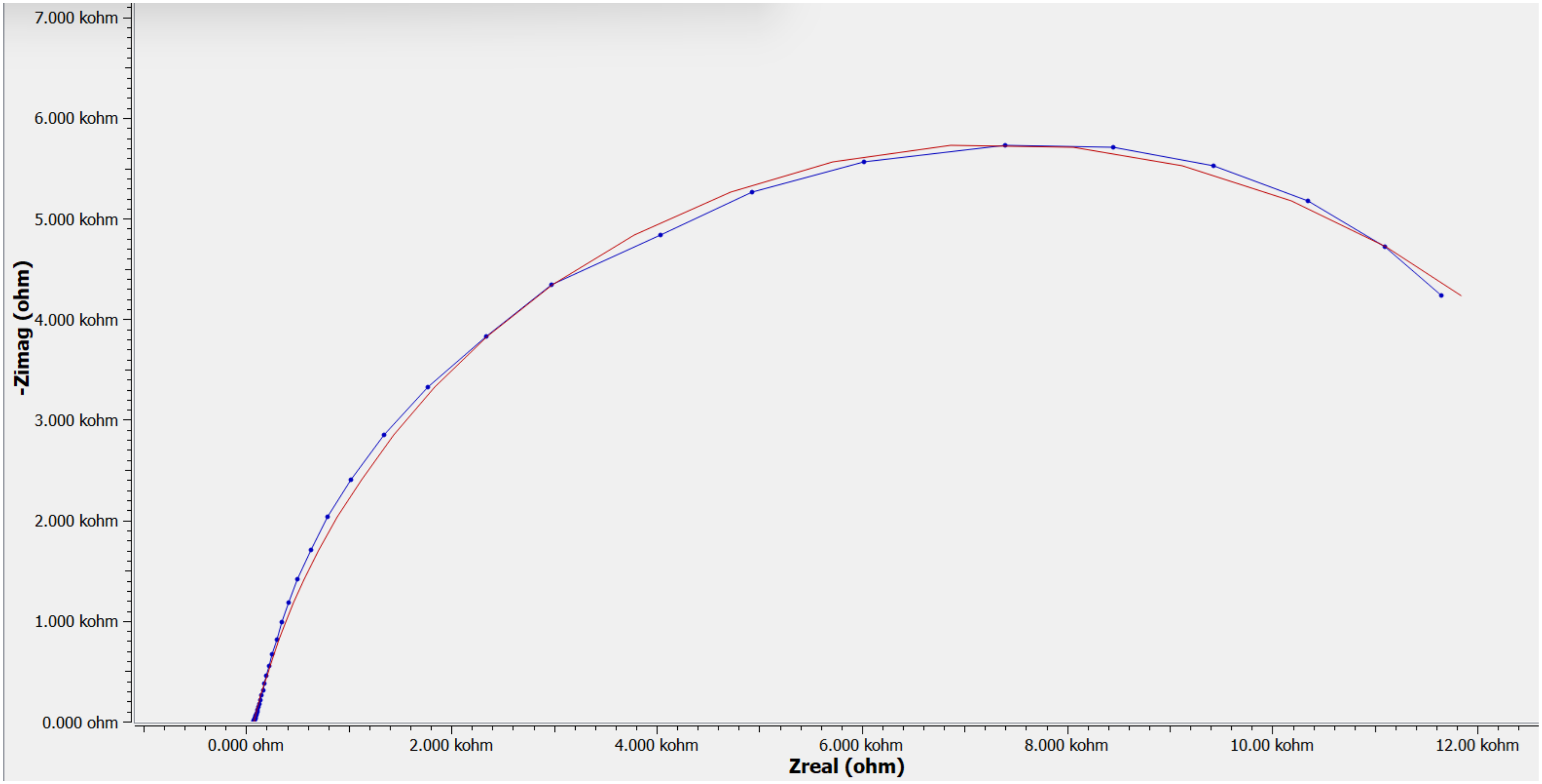

Figure S60. EIS of gold-MUA-CD144AB-EV(std. 5) E14.

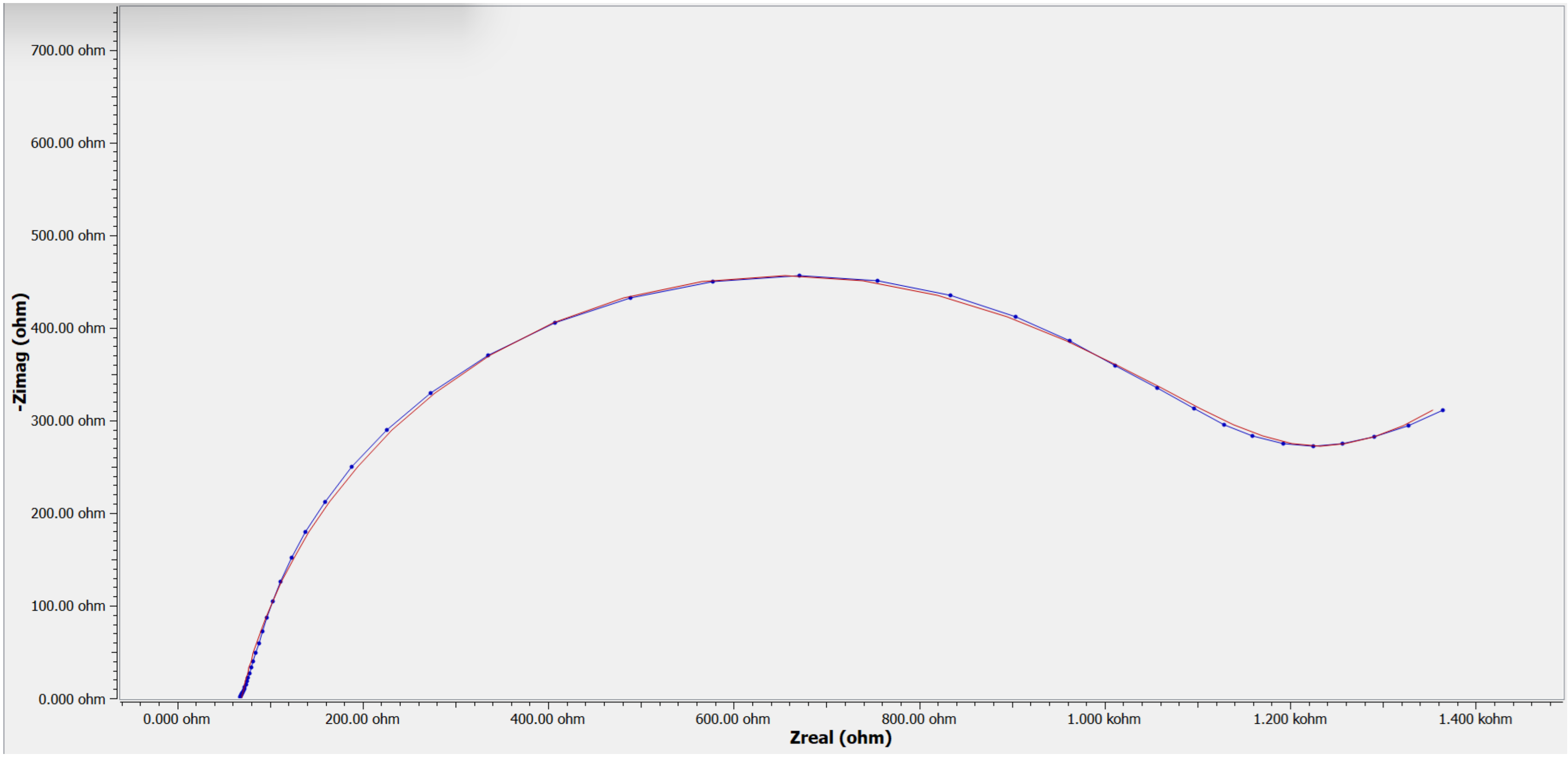

Figure S61. EIS of gold bare E15.

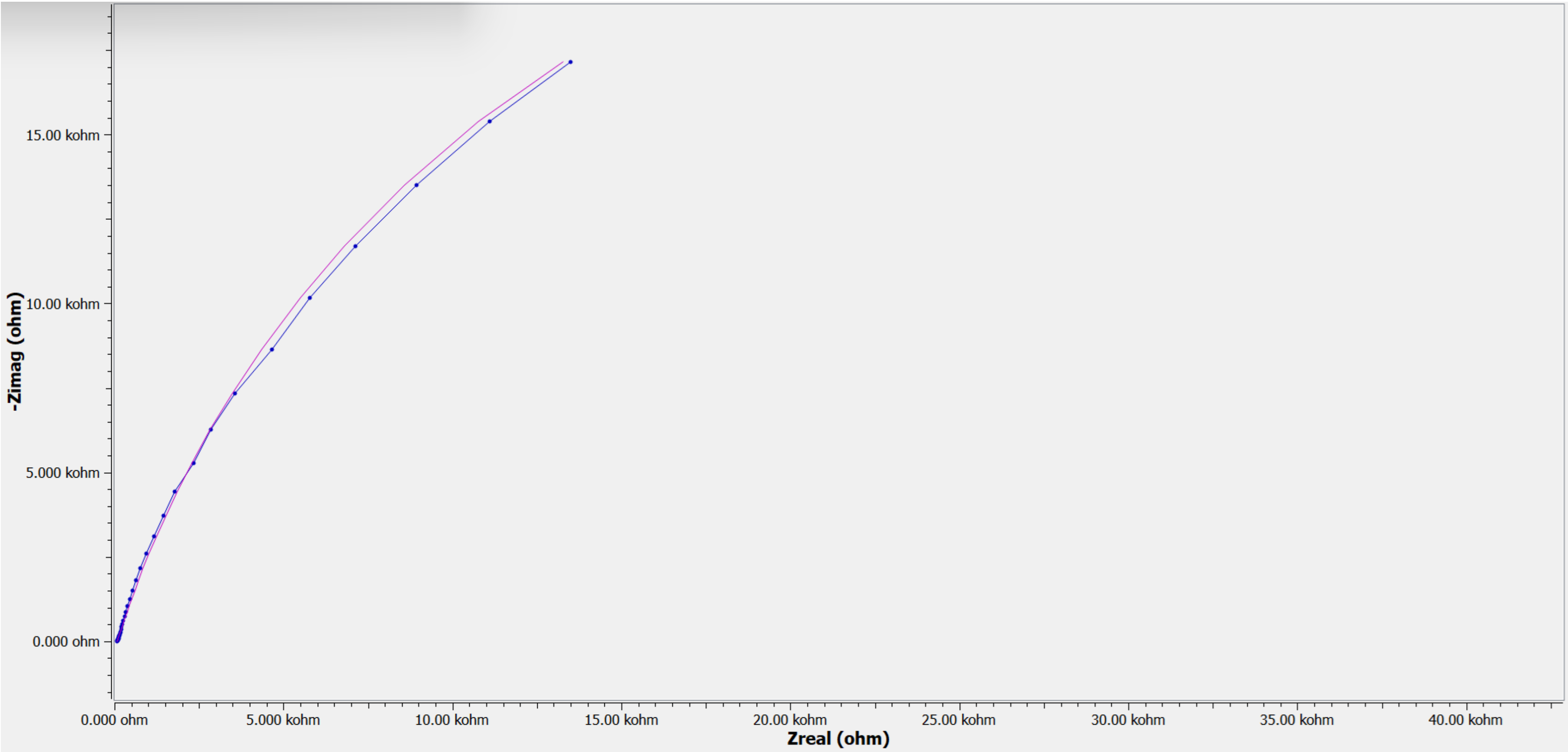

Figure S62. EIS of gold-MUA E15.

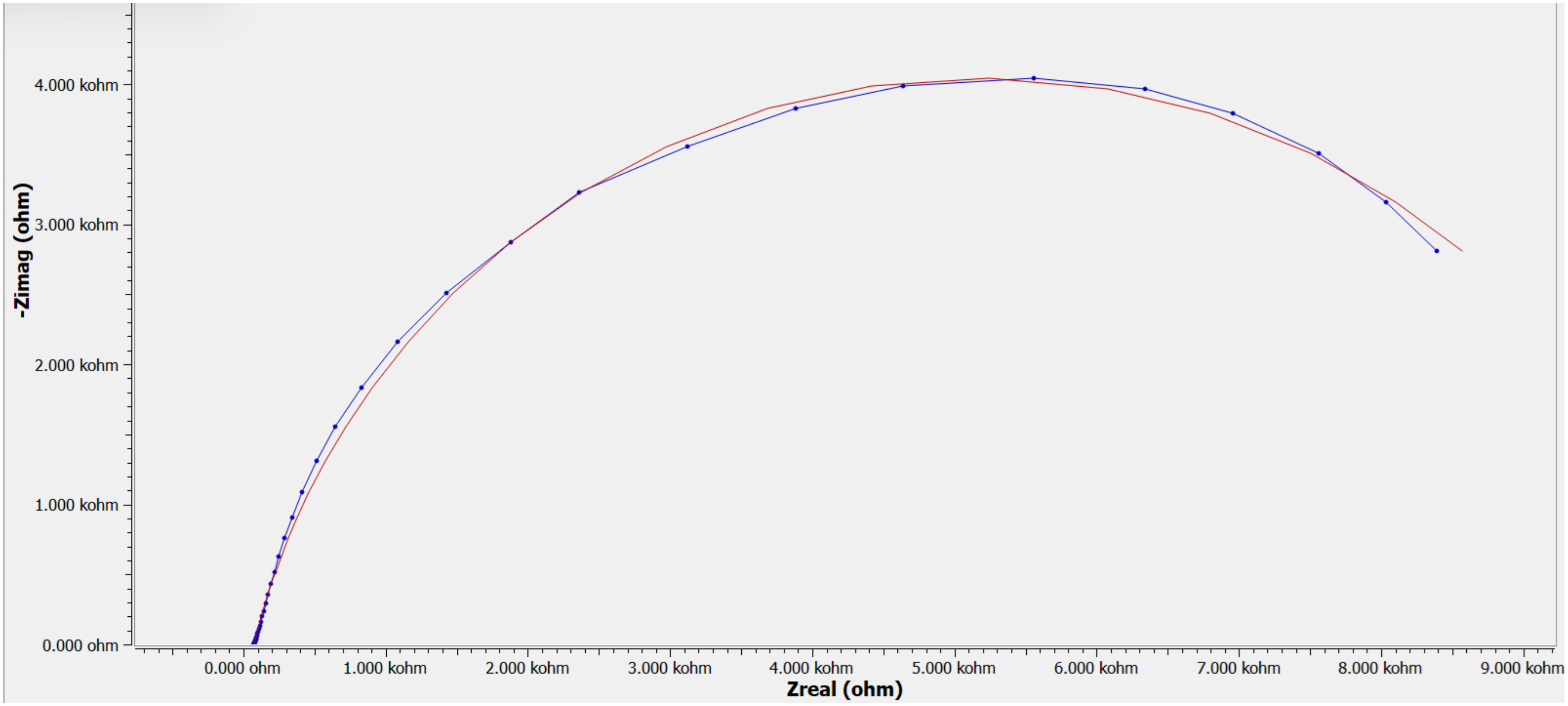

Figure S63. EIS of gold-MUA-CD144AB E15.

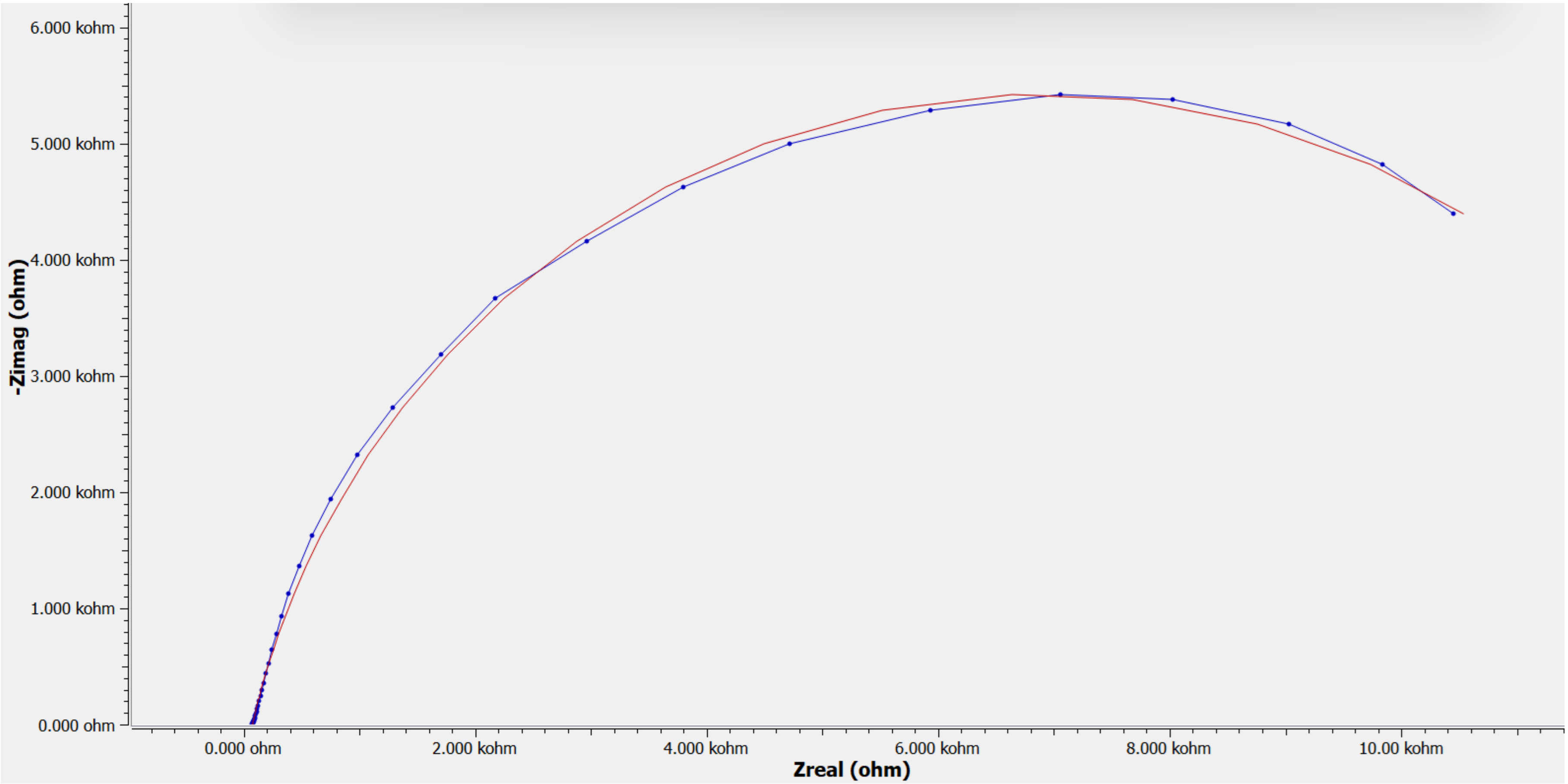

Figure S64. EIS of gold-MUA-CD144AB-EV(std. 5) E15.
